# Aldehyde-mediated inhibition of asparagine biosynthesis has implications for diabetes and alcoholism†

**DOI:** 10.1039/d3sc06551k

**Published:** 2024-01-15

**Authors:** Tobias John, Nadia Saffoon, John Walsby-Tickle, Svenja S. Hester, Felix A. Dingler, Christopher L. Millington, James S. O. McCullagh, Ketan J. Patel, Richard J. Hopkinson, Christopher J. Schofield

**Affiliations:** a Chemistry Research Laboratory, Department of Chemistry and the Ineos Oxford Institute for Antimicrobial Research, University of Oxford 12 Mansfield Road Oxford OX1 3TA UK christopher.schofield@chem.ox.ac.uk; b Nuffield Department of Medicine, Target Discovery Institute, University of Oxford Oxford UK; c MRC Weatherall Institute of Molecular Medicine, John Radcliffe Hospital/Headley Way Oxford OX3 9DS UK; d Leicester Institute for Structural and Chemical Biology and School of Chemistry, University of Leicester, Henry Wellcome Building Lancaster Road Leicester LE1 7RH UK richard.hopkinson@leicester.ac.uk

## Abstract

Patients with alcoholism and type 2 diabetes manifest altered metabolism, including elevated aldehyde levels and unusually low asparagine levels. We show that asparagine synthetase B (ASNS), the only human asparagine-forming enzyme, is inhibited by disease-relevant reactive aldehydes, including formaldehyde and acetaldehyde. Cellular studies show non-cytotoxic amounts of reactive aldehydes induce a decrease in asparagine levels. Biochemical analyses reveal inhibition results from reaction of the aldehydes with the catalytically important N-terminal cysteine of ASNS. The combined cellular and biochemical results suggest a possible mechanism underlying the low asparagine levels in alcoholism and diabetes. The results will stimulate research on the biological consequences of the reactions of aldehydes with nucleophilic residues.

## Abstract 2

Asparagine levels are reduced in patients with diabetes and alcoholism. A plausible mechanism is that aldehydes elevated in these diseases react with the N-terminal cysteine of asparagine synthetase B, so inhibiting asparagine production.

## Graphical abstract

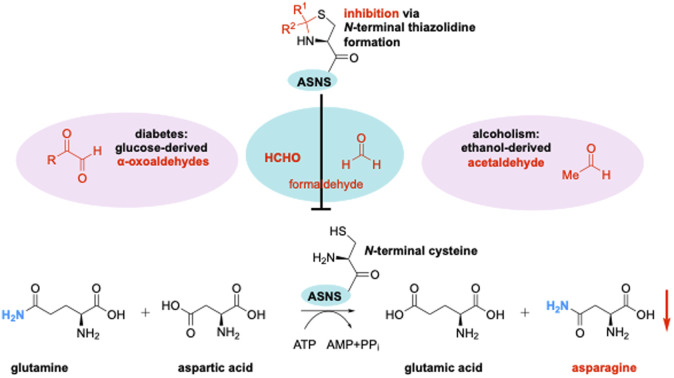

## Introduction

Above threshold levels, reactive aldehydes including formaldehyde (HCHO) and acetaldehyde (AcH) are harmful to animals.^1^ Certain diseases also result in increased levels of reactive aldehydes. Patients with alcoholism have increased AcH levels, while elevated glucose oxidation in type 2 diabetes patients produces membrane-permeable α-oxoaldehydes such as methylglyoxal (MGO), glyoxal, and glyoxylic acid.^2^ HCHO, which is used as a fumigant and preservative, is also produced naturally in cells, including by the action of *N*-methyl demethylases acting on histones and nucleic acids.^3,4^

There are multiple links between aldehyde detoxification and reactions with cysteinyl thiols.^9–13^ Of the common amino acids, HCHO reacts most efficiently with cysteine, forming thioproline;^9–11^ incorporation of thioproline instead of proline into proteins is proposed to contribute to HCHO's toxicity.^11^ In animals, HCHO detoxification involves its reaction with the cysteinyl thiol of the tripeptide glutathione (GSH), giving a substrate for alcohol dehydrogenase 5 (ADH5).^5,6^ Mice deficient in ADH5 are particularly sensitive to exogenous HCHO.^1,7^ Biological HCHO sensors employ conserved cysteines that are crucial for enabling detoxification responses.^8^ Like HCHO, MGO is metabolised by the GSH-dependent glyoxalase pathway, which involves initial reaction with GSH to give a hemithioacteal that undergoes glyoxalase 1-catalysed isomerisation.^12^ Aldehyde dehydrogenases (ALDHs), which are the primary AcH-metabolising enzymes, also employ a nucleophilic cysteine in catalysis.^13^

Asparagine levels are decreased in patients suffering from alcoholism, type 2 diabetes and obesity, but the molecular mechanisms responsible for these observations have been unknown.^14–20^ Prokaryotes have two types of asparagine synthetase (ASNS): ASNS A uses ammonia as a nitrogen source, whereas ASNS B employs an N-terminal nucleophilic cysteine to release ammonia from glutamine (Fig. S1†).^21^ Mammals only have the type B ASNS.^21^ Here we show that ASNS B catalysis is inhibited by reaction of its N-terminal cysteine with HCHO and other disease-relevant reactive aldehydes, an observation that is consistent with clinically observed asparagine deficiencies.

## Results

### Aldehydes decrease cellular asparagine levels by regulating asparagine synthetase

**Fig. 1:**
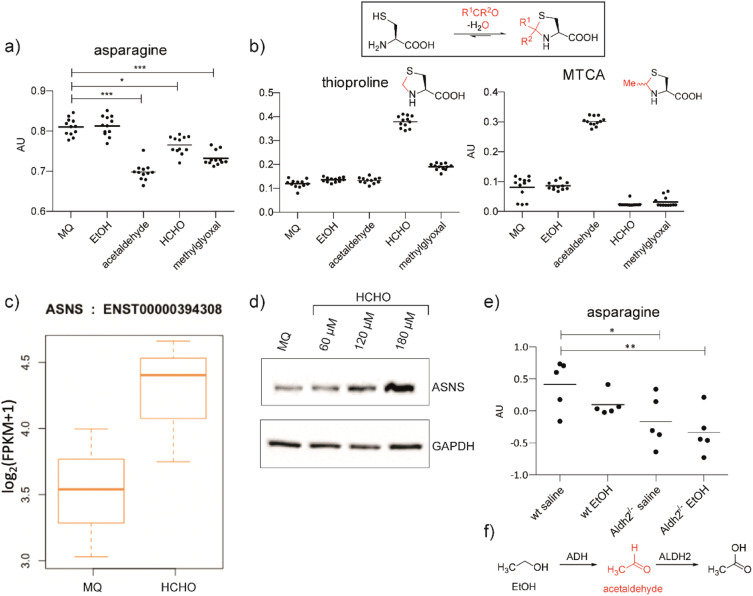
Aldehyde addition decreases asparagine and increases cysteine-derived thiazolidine levels in human cells. (a) Asparagine, thioproline and 2-methylthiazolidine-4-carboxylic acid (MTCA) levels in HEK293T cells treated with aldehydes. Full data set: Fig. S3.† (b) Reaction of cysteine with aldehydes/ketones. Structures of the cysteine-HCHO adduct (thioproline) and the cysteine-AcH adduct (MTCA) are shown; the cysteine-MGO adduct was not detected under our conditions. Independent replicates: Fig. S2b.† (c) ASNS mRNA levels in HEK293T cells treated with HCHO (150 μM, then a second dose – 225 μM after 24 hours) compared to water (Milli-Q water, MQ). ASNS gene, fc = 0.58, *p* = 0.298, *q* = 0.594. Errors: SD of the mean (*n* = 3). (d) Western blots showing protein levels of ASNS and GAPDH from HEK293T cells treated with the indicated amount of HCHO or water for 14 hours. Independent replicates: Fig. S8.† (e) MS analyses of asparagine levels in mouse livers. Full analyses: Fig. S12.† (f) Oxidation of ethanol (EtOH) by alcohol dehydrogenase (ADH) and of AcH by aldehyde dehydrogenase 2 (ALDH2).^6^

We hypothesised that aldehydes may react with specific cellular components to cause reductions in asparagine levels. To investigate this, we used mass spectrometry (MS) to measure asparagine levels in HEK293T cells treated with the biologically relevant aldehydes AcH,^22^ MGO,^2^ and HCHO.^23^ Importantly, when using a cell medium low in asparagine (Fig. S2a†), apparently non-cytotoxic doses of the aldehydes induced a decrease in asparagine levels (Fig. 1a and S2b, S3, S4†). No consistent depletion of the aldehyde scavengers cysteine or glutathione was observed, an observation potentially reflecting their high intracellular concentrations and reversible reactions with aldehydes (Fig. S2 and S3†).^24^

Levels of arginine and tryptophan, which are metabolically distant from asparagine, were not consistently affected by the aldehyde treatments (Fig. S2b and S3†). However, alanine was depleted in cells treated with HCHO, AcH, or MGO (Fig. S2b and S3†). This observation is consistent with the proposal that asparagine is an amino acid exchange factor regulating levels of other amino acids/related 2-oxo acids (experiments were conducted both in the presence and in the absence of GlutaMAX, Fig. S2b and S3†).^25^ Histidine and phenylalanine levels were also depleted, but not consistently with the different aldehyde treatments (Fig. S3†). Treatment with ethanol, *i.e.* the precursor of AcH, did not result in asparagine depletion in HEK293T cells (Fig. 1a), an observation that is consistent with the reported lack of alcohol dehydrogenase gene expression in HEK293T cells.^26^ Analyses in HCHO-treated LN-18 cells revealed evidence for asparagine depletion, although the effect was weaker than in HEK293T cells (Fig. S5†). This observation may be due, at least in part, to the use of non-dialysed foetal bovine serum which contains relatively high asparagine levels. Consistent with this, depletion of amino acids was not observed with HEK293T cells incubated with non-dialysed foetal bovine serum (Fig. S6 and S7†).

Cysteine reacts readily with HCHO and AcH to give thioproline and 2-methylthiazolidine-4-carboxylic acid (MTCA), respectively (Fig. 1).^9,10,27^ Treatment of HEK293T cells with HCHO or AcH led to increased thioproline and MTCA levels, respectively (Fig. 1b). Thioproline formation was also observed in HCHO-treated LN-18 cells (Fig. S5†). The reaction of cysteine and AcH to give MTCA is reported in blood^28^ and human parasites;^29^ the evidence presented here also shows that MTCA can occur in human cells. The cysteine-MGO adduct was not detected, possibly reflecting its lower stability, relative to thioproline or MTCA, under our analytical conditions.^10^

Analyses on HCHO-treated HEK293T cells indicated that mRNA encoding for human ASNS, the only human enzyme that produces asparagine,^30^ is upregulated by HCHO treatment (Fig. 1c). Treatment at relatively low HCHO concentrations resulted in an apparent dose-dependent increase in ASNS protein levels (observed by western blot analyses) (Fig. 1d and S8†). However, no increase in ASNS protein levels was observed after treatment with HCHO at 300 μM (Fig. S9†) and no evidence for a substantial HCHO-mediated effect on ASNS stability was accrued from initial studies with the protein synthesis inhibitor cycloheximide. Although further biological work is required, these observations suggest that low doses of HCHO might increase ASNS levels by increasing mRNA levels, but that HCHO does not, at least substantially, modulate ASNS protein stability (Fig. S9†).^31^

Studies were conducted using two mouse models with compromised aldehyde metabolism. Adh5^−/−^ mice are deficient in metabolising the GSH-HCHO adduct *S*-hydroxymethylglutathione,^7^ and are therefore unable to detoxify HCHO *via* the GSH-dependent pathway. We also carried out studies on Aldh2^−/−^ mice,^32^ which are deficient in the key AcH detoxification enzyme ALDH2. After treatment with the AcH and HCHO precursors ethanol and methanol, respectively, we analysed livers of Adh5^−/−^, Aldh2^−/−^ and wild-type (wt) mice for changes in cysteine-derived thiazolidine adducts, and asparagine and ASNS levels. Aldehyde precursors were used due to the poor penetrance throughout the organism and the potential for localised off-target toxicity of aldehydes. Compared to wt mice, thioproline levels were substantially elevated in Adh5^−/−^ mice treated with methanol,^33^ supporting the proposed importance of cysteine as a HCHO scavenger (Fig. S10a†). The cysteine-AcH adduct MTCA, was not detected in EtOH treated mice (Fig. S11†), possibly reflecting the relative instability of MTCA compared to thioproline.

Importantly, ethanol-treated Aldh2^−/−^ mice showed clearly decreased asparagine levels (Fig. 1e and S12†). This was not observed with methanol-treated mice under the tested conditions (Fig. S10a†). The observed difference between ethanol and methanol treatments might, in part, reflect the more efficient reaction of HCHO than AcH with cysteine, leading to better sequestration of HCHO in cells. HCHO formation is also likely to be less efficient than AcH formation in mouse livers as a consequence of poor ADH activity with methanol. Western blot analyses on the liver samples from Adh5^−/−^ mice treated with methanol and from Aldh2^−/−^ mice treated with ethanol displayed only mildly elevated ASNS levels relative to wt mouse livers (Fig. S11 and S10b†). Although other mechanisms are possible, including hormesis-type effects, the collective cellular and mouse studies suggest that aldehydes decrease cellular asparagine levels in a manner consistent with ASNS inhibition.

### Aldehydes form stable thiazolidine adducts with N-terminal cysteines

Studies then focused on determining whether aldehydes inhibit ASNS. Given free cysteine reacts efficiently with aldehydes to give thiazolidine rings, *e.g.* thioproline and MTCA,^10^ we envisioned that the N-terminal catalytic cysteine in N-terminal nucleophilic (Ntn) hydrolases such as ASNS^34^ may be particularly prone to reactions with aldehydes giving stable adducts.

Initially, we conducted studies with HCHO and dipeptides bearing either an N-terminal serine (SerGlu, 1), threonine (ThrGlu, 2), or cysteine (CysGlu, 3) residue; these three residues are commonly found at the N-terminus of Ntn hydrolases. Glutamic acid was used as the C-terminal residue for solubility reasons and because it does not react with aldehydes to give a stable adduct.^10^ The dipeptides were reacted with a 10-fold excess of HCHO at pD 9.4 (to promote conversion to the HCHO adducts^10^) and analysed by ^1^H NMR.

**Fig. 2:**
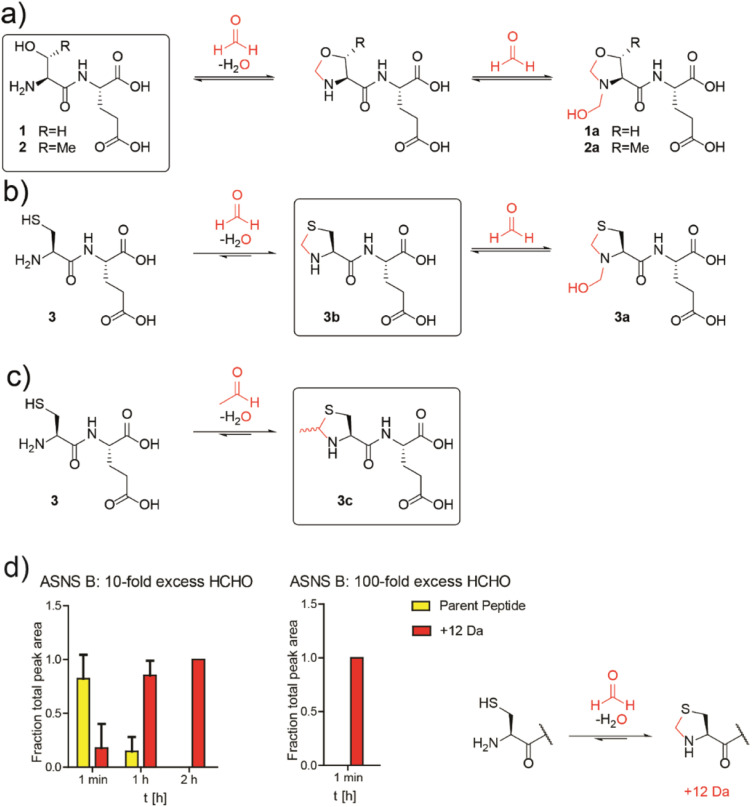
N-Terminal cysteine residues react with formaldehyde and acetaldehyde to form stable thiazolidines. (a) Peptides 1 and 2 react reversibly with HCHO to form N-terminal oxazolidines 1a and 2a, which are unstable under HPLC conditions, or on excess GSH addition (40 : 1 GSH : dipeptide). Full analyses: Fig. S13, S14 and S17a/b.† (b) Peptide 3 reacts with HCHO to form thiazolidine 3b and hemiaminal 3a; the latter of is unstable under HPLC conditions. Addition of a 40-fold excess GSH to 3a results in 3b. Full analyses: Fig. S15–S17c.† (c) Peptide 3 reacts with AcH to form N-terminal thiazolidine 3c, which is stable under HPLC conditions (Fig. S18†), but which degrades releasing AcH over time. Reactions employed a 10-fold excess of aldehyde (pD 9.4, 12 hours). Boxed structures are the major species isolated after HPLC purification. (d) Reaction of an ASNS B 7-residue N-terminal peptide (NH_2_-CSIFGVF-NH_2_) with HCHO (10-fold or 100-fold excess) at pH 7.4 and 50 mM potassium phosphate. HCHO adducts: red (+12 Da). Parent (unreacted) peptide: yellow. Reactions were monitored following addition of HCHO at the indicated times. Integrated areas of peaks were used (Fig. S22†). Errors: SEM (*n* = 3, technical repeats).

The peptides reacted to form N-terminal 5-membered oxazolidine or thiazolidine rings (Fig. 2a and b and S13–S15†). The HCHO-derived oxazolidines 1a and 2a could not be isolated by HPLC and were unstable under our LC/MS conditions. By contrast, thiazolidine 3b, but not hemiaminal 3a, was stable under our purification conditions (Fig. S16†) and 3b, but not 3a, was stable in the presence of a 40-fold excess of GSH (Fig. S17†). Cysteine-dipeptide 3 reacted with AcH to give the stable epimeric thiazolidines 3c. ^1^H NMR time-course analyses on 3c consistently revealed the presence of starting cysteine-dipeptide 3 and AcH, implying 3c is relatively less stable than the analogous HCHO-derived thiazolidine 3a (Fig. 2c and S18†).

We then investigated how HCHO reacts with a full-length type B ASNS. We used ASNS B from *Escherichia coli* (hereafter ASNS B) rather than human ASNS due to its analogous catalytic profile to human ASNS and its more efficient production in recombinant form.^35^ ASNS B was prepared as described,^35^ then treated with 10-fold, 100-fold and 1000-fold excesses of HCHO (20 minutes, 37 °C). Protein-observed MS studies under denaturing conditions revealed formation of multiple adducts with distinct masses (Fig. S19†), with many likely being hydroxymethyl adducts formed by reaction of HCHO with nucleophilic side chains. A clear +12 Da mass shift relative to unmodified ASNS B was observed with a 100-fold excess of HCHO (along with other adducts), suggesting efficient formation of at least one methylene-containing adduct. Importantly, evidence for the presence of a +12 Da adduct on the N-terminal cysteine of ASNS B was accrued from MS fragmentation analyses (Fig. S21†). Additional analyses with a dipeptide and a 7-residue peptide mimicking the N-terminus of ASNS B revealed formation of a +12 Da adduct on HCHO incubation, consistent with thiazolidine formation (Fig. 2d and S22†). Quantitative studies on the 7-residue peptide by MALDI MS indicated that the extent of thiazolidine formation is dependent on the HCHO concentration (Fig. 2d). Substitution of the N-terminal cysteine with alanine ablates stable product formation (Fig. S23b†).

Studies conducted on samples containing ASNS B and 1000-fold excesses of AcH, glyoxylic acid, glyoxal, MGO, or acrolein all displayed evidence for adduct formation (Fig. S19†). With glyoxylic acid and MGO, up to four adducts were formed, while AcH apparently reacted with ASNS B to form one distinct adduct. By contrast, no reactions were observed when ASNS B was incubated with the less reactive carbonyl compounds pyruvate, acetone or glucose (Fig. S20†). Incubation of a peptide mimicking the N-terminus of ASNS B with a 100-fold excess of AcH manifested formation of a single +26 Da adduct, consistent with MTCA-type thiazolidine formation (Fig. S23a†). We attempted to identify the N-terminal thiazolidine modification on ASNS purified from human cells treated with HCHO. However, despite multiple attempts, MS analyses of trypsin-digested immunoprecipitated ASNS from HEK293T cells treated with HCHO did not provide coverage of the N-terminal tryptic peptide of ASNS, thus precluding identification of the thiazolidine adduct (Fig. S24†).

### Aldehydes inhibit the N-terminal glutaminase domain of asparagine synthetase

**Fig. 3:**
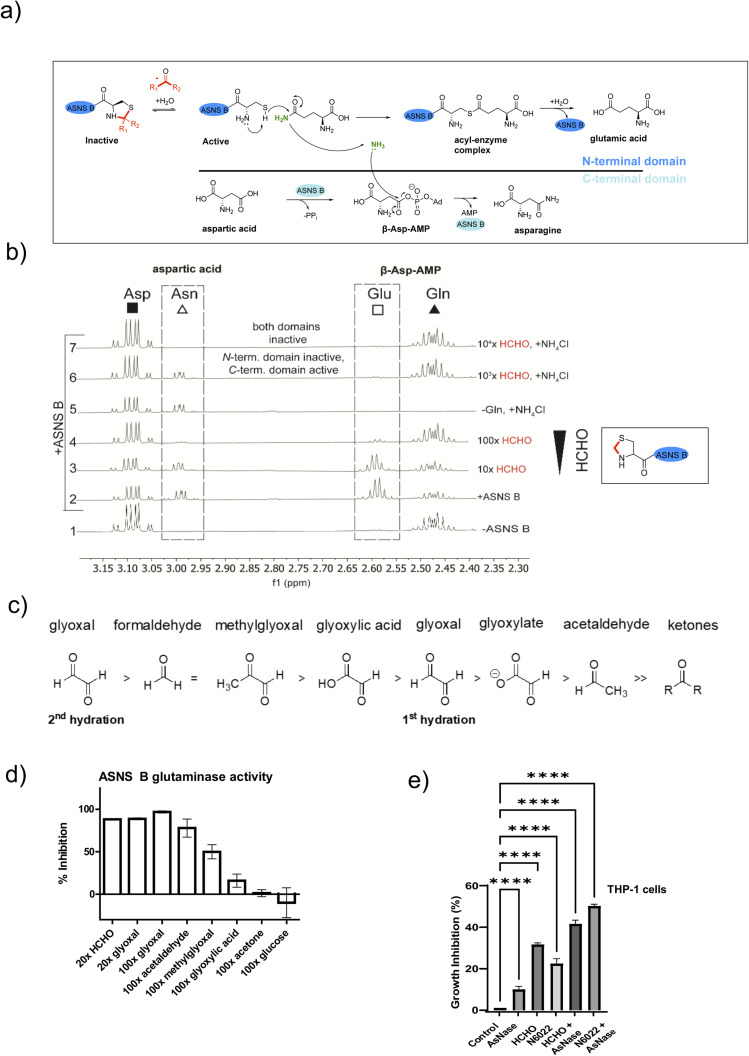
HCHO selectively inhibits the N-terminal glutaminase, but not the C-terminal synthetase, partial reaction of ASNS B. (a) Reactions involved in ASNS B catalysis (see also Fig. S1†). The glutamine amide NH_2_, which is transferred to aspartic acid, is in green.^42^ (b) ^1^H NMR (700 MHz) analysis of the C3 (Asp, Asn) and C4 (Gln, Glu) methylene groups in ASNS B-catalysed conversion of Gln (*δ*_H_ 2.42–2.51 ppm) to Glu (*δ*_H_ 2.55–2.61 ppm) and Asp (*δ*_H_ 3.03–3.13 ppm) to Asn (*δ*_H_ 2.97–3.00 ppm) (panel 2, +ASNS B). The glutaminase reaction, which is dependent on the ASNS B N-terminal cysteine, is inhibited by HCHO (panels 3, 4). Panel 6: the C-terminal synthetase domain of ASNS B is active in the presence of Asp, ATP, NH_4_Cl and a 10^3^ excess of HCHO, but not when a 10^4^-fold excess HCHO is pre-incubated with ASNS B (panel 7). See Fig. S25† for an independent replicate. (c) Order of hydration equilibrium constants for reactive carbonyl compounds; note glyoxal can undergo two hydrations.^36^ (d) ASNS B was pre-incubated with the indicated molar excess of carbonyl compound (or MQ water control) (45 minutes, 37 °C), then added to the assay components, with monitoring by ^1^H NMR (700 MHz, errors: SD of the mean (*n* = 2 independent repeats); see Fig. S26† for details). (e) Inhibition of THP-1 acute monocytic leukaemia cell growth by treatment with HCHO (80 μM), the HCHO metabolism inhibitor N6022 (10 μM), and/or ASNase (0.05 U mL^−1^). Growth inhibition (%) = 100 − (treatment group OD/non-treatment OD) × 100; OD = optical density. (*p value = 0.01 to 0.05, **p value = 0.001 to 0.01, ***p value = 0.0001 to 0.001, ****p value < 0.0001, *n* = 3 technical repeats. Biological replicates of the experiment shown in Fig. S27,†*n* = 4 biological repeats).

ASNS B (and human ASNS) catalyse asparagine biosynthesis in a two-step process involving distinct domains (Fig. 3 and S1†). First, the N-terminal cysteine of the glutaminase domain reacts with glutamine giving glutamate and ammonia; the latter travels through a tunnel to the synthetase domain, where it reacts with activated aspartic acid (β-aspartyl-AMP), forming asparagine (Fig. 3a). The results described above indicate that aldehydes might inhibit ASNS by reacting with its N-terminal cysteine. To test this, we used ^1^H NMR to monitor ASNS B activity/inhibition (Fig. 3 and S25, S2†). In the absence of an aldehyde, clear evidence for asparagine formation was accrued using either glutamine or ammonium chloride as the ammonia source (Fig. 3b).^35^ Near-complete inhibition was observed when ASNS B was pre-incubated with a 100-fold HCHO excess. However, no inhibition was observed when using ammonium chloride instead of glutamine (Fig. 3b). This observation implies that the ASNS B glutaminase activity is inhibited by a 100-fold excess of HCHO, whereas the synthetase activity is not inhibited under these conditions (Fig. 3b). When ASNS B was pre-incubated with a 10^4^-fold excess of HCHO, asparagine formation could not be rescued by addition of ammonium chloride. This finding implies that both the glutaminase and the synthetase partial reactions of ASNS B are inhibited at very high HCHO concentrations, possibly as a consequence of widespread protein-HCHO adduct formation with consequent (partial) denaturation (Fig. 3b).

Other biologically relevant aldehydes were tested as ASNS B inhibitors. Pre-incubation with an excess of acetone or glucose did not inhibit ASNS B (Fig. 3d and S26d, e†), while a 100-fold excess of glyoxylic acid mildly inhibited ASNS B. By contrast, a 100-fold excess of AcH or MGO substantially inhibited ASNS B activity (Fig. 3d and S26d, e†), while a 20-fold excess of HCHO (Fig. 3d, S26c, e†) or glyoxal was sufficient to almost completely ablate activity. Inhibition was also observed with acrolein (Fig. S26d†). These observations correlate with the expected electrophilic properties of carbonyl groups of the tested compounds (Fig. 3c).^36^

### Aldehydes inhibit growth of leukemia cells

Acute lymphoblastic leukemia (ALL) cells manifest reduced levels of asparagine. This property is exploited in ALL treatment, where treatment with the hydrolase asparaginase (ASNase) is used to limit asparagine availability and promote ALL cell death.^37^ ASNase is also reported to have an antiproliferative effect in acute myeloid leukaemia (AML).^38,39^

We proposed that elevated aldehyde levels might affect the growth of leukemia cells and accentuate the effects of ASNase. To test this, we subjected THP-1 cells, which are used as a model of acute monocytic leukemia (an AML sub-type), to treatment with HCHO, ASNase, or the ADH5 inhibitor N6022.^40^ Treatment of THP-1 cells with HCHO, N6022 or ASNase manifested inhibition of cell growth; treatment with mixtures of HCHO/N6022 and ASNase ablated growth under the tested conditions (Fig. 3e and S27†). Although further work is required (including in ALL models), our initial observations suggest elevated aldehyde concentrations, *e.g.* as induced by inhibiting aldehyde metabolism or potentially by treating with aldehyde-releasing small molecules,^41^ have potential to inhibit cancer cell growth. They also imply that inhibition of asparagine biosynthesis may be of therapeutic utility beyond ALL treatment.

## Conclusions

Our studies show that treatment of cells with non-cytotoxic amounts of reactive aldehydes can induce decreased asparagine levels. This observation correlates with biochemical analyses revealing aldehydes inhibit ASNS *via* reaction with its catalytically important N-terminal cysteine. The combined cellular and biochemical results thus suggest ASNS inhibition is a possible mechanism underlying the low asparagine levels in alcoholism and diabetes. It should, however, be noted that the relatively high aldehyde concentrations used in our work and in some other disease related studies likely result in a multitude of reactions. Coupled with the complexity of amino acid and related metabolism, it is thus possible that more than one mechanism (including involving reactions with cysteines other than in ASNS) contribute to perturbed asparagine levels. There is also a possibility of hormesis-type effects at lower aldehyde concentrations, possibly involving the induction of anti-stress mechanisms. Nonetheless, aldehyde-mediated ASNS inhibition is a mechanistically attractive explanation for the observed reductions in asparagine levels.

Under normal conditions, reactive aldehydes are removed by efficient metabolism, *e.g.* reaction with GSH or the direct action of cysteine-containing ALDH enzymes.^6^ The efficiency of the reactions of reactive aldehydes with cysteine itself is evidenced by the elevated levels of thioproline and MTCA in cells treated with HCHO and AcH respectively (Fig. 1b). Given the stability of these adducts (particularly for the HCHO adducts^10^), these observations suggest that cysteine can act as an efficient aldehyde scavenger. We propose that, under diseased conditions such as type 2 diabetes and alcoholism,^2,15^ normal aldehyde detoxification pathways become saturated, resulting in elevated concentrations of free aldehydes. These aldehydes can then induce functionally significant events, *e.g.* inhibition of ASNS that manifests as clinically observed reductions in asparagine.^14–20^

The results show that reactive aldehydes inhibit ASNS-catalysed generation of ammonia from glutamine *via* N-terminal thiazolidine formation (Fig. 2), but do not inhibit asparagine formation from ammonia (at least at lower aldehyde concentrations, Fig. 3). Thus, ASNS catalysis in cells may proceed at a reduced rate even after reaction of its N-terminal cysteine with an aldehyde. Such a mechanism may help cells to continue making asparagine under aldehyde-stressed conditions.

It is important to state that the set of cellular aldehydes that inhibit ASNS (or other targets) is unknown and may change in a context-dependent manner. The presence of high levels of diet-derived aldehydes may indirectly cause an increase in levels of endogenously produced aldehydes. For example, high levels of diet-derived ethanol/AcH may lead to elevated levels of endogenously produced HCHO or other aldehydes that could inhibit ASNS. It is also possible that alkylidene donors other than aldehydes react with N-terminal cysteines in an analogous manner to aldehydes; recent work reveals 5,10-methylenetetrahydrofolic acid as such a candidate.^43^ Despite these complexities, the functionally relevant reaction of certain aldehydes with the N-terminal cysteine of ASNS provides a potentially direct link between diet and amino acid metabolism.

We observed evidence that ASNS levels can be increased in response to both HCHO and AcH (Fig. 1c and d). Future work can involve studies on the underlying mechanism(s) of ASNS elevation, which might involve a specific aldehyde sensing/response mechanism or be a consequence of the induced integrated stress response (ISR), which is reported to regulate ASNS levels.^44^ Indeed, our RNA-seq analyses suggest upregulation of ISR genes on HCHO treatment (data not shown), possibly in a manner dependent on nuclear factor erythroid 2-related factor 2 (NRF2).^45^

All characterised HCHO sensing proteins employ cysteine residues that react with HCHO, though subsequent reaction with another residue to form a methylene bridge can occur.^8^ Interestingly, the cysteines involved in HCHO sensing mechanisms are not located at the protein N-terminus, possibly because thiazolidines formed with reactive aldehydes are too stable to be useful in a reversible sensing mechanism. In the case of the *Escherichia coli* HCHO transcription factor sensor FrmR, HCHO reacts to form a methylene bridge between an internal cysteine and the N-terminal proline.^46^ This observation is interesting because N-terminal prolines can react with HCHO to form stable bicyclic rings.^47^

N-Terminal cysteine, serine and threonine residues are of particular interest with respect to reactions with aldehydes because the N-terminal amino group lowers the p*K*_a_ of the alcohol/thiol side chain.^48,49^ It seems likely that enzymes other than ASNS with nucleophilic N-terminal cysteines react with aldehydes in a functionally relevant manner. In humans, there are relatively few (∼95) predicted proteins with N-terminal cysteines,^50^ a factor that may in part reflect selection against N-terminal cysteines due to their nucleophilicity and propensity to react with aldehydes.

Proteins with N-terminal cysteines have been shown to be regulated by a variant of the N-end rule involving oxygen availability-dependent oxidation of the N-terminal cysteine.^51^ The cysteine dioxygenases catalysing this oxidation require the amino and thiol groups of their N-terminal cysteine substrates for activity.^51^ Their activity is therefore likely blocked by N-terminal thiazolidine formation. This is also likely to be the case for other N-terminal modifying enzymes such as N-terminal protein acetyl transferases, which are common in most life forms.^52^

Searching for enzymes and biochemicals that reverse aldehyde-mediated protein modifications/cross-linking (including involving thiazolidines) is of interest. Precedent for such repair comes from the roles of the SPRTN protease in cleaving DNA–protein cross-links, including those potentially induced by HCHO.^53^ Diet-derived aldehydes such as AcH can also cause double-stranded breaks in DNA promoting recombination repair pathways.^54^

In terms of hypoxic regulation, it is notable that certain cysteine dioxygenases and HCHO-producing 2-oxoglutarate dependent demethylases are proposed to be hypoxia sensors.^55^ Further, some 2OG-dependent demethylases are gene targets of the hypoxia inducible factors^51,56^ that regulate transcription in a manner regulated by oxygen availability.^57^ We speculate that the HCHO (or HCHO precursors) produced by some demethylases might not be a simple toxic by-product, but have functional relevance by reacting with proteins and/or other biomolecules including nucleic acids and carbohydrates.

It is possible that the dynamic reaction of aldehydes with nucleophiles is a general and tuneable mechanism contributing to the regulation of bio(macro)molecule function and stability. However, obtaining a comprehensive picture of such interactions is challenging and will likely require new methods. Studies with isolated components thus remain of importance and have shown that relatively stable hemiaminal products can be formed by reactions of aldehydes with nucleic acids.^58^ However, by contrast with the relatively stable –N/O/S–CHR–S– cyclic products formed by reactions of aldehydes with thiols and other appropriate nucleophiles, most characterised –N/O–CHR–N/O– type adducts formed by reaction of aldehydes with nucleic acids (and histones) are less stable.^10,58^ It thus seems likely that a multitude of dynamic equilibria exist between reactive small molecule carbonyl compounds and biomacromolecular nucleophiles, both within and outside of cells. It is possible that one role of reversible reactions of this type is to protect biomacromolecules from more damaging less reversible modifications, that may impact on enzyme catalysis (as described here for ASNS), protein stability, or cause mutation (*e.g. via* irreversible alkylation).

Acute lymphoblastic leukemia (ALL) cells have unusually low levels of asparagine and ALL is treated with asparaginase (ASNase).^37^ ASNase also has an antiproliferative effect in acute myeloid leukaemia (AML).^38,39^ We found that treatment of THP-1 leukemia cells with ASNase, HCHO, or the ADH5 inhibitor N6022 inhibited cell growth, consistent with links between aldehydes and asparagine and disease. Recent studies on pancreatic cancer have identified an unexpected role for asparagine and ASNS in enabling proliferation when respiration is limited.^59^ Interestingly, ALL survivors are reported to have a greater incidence of diabetes,^60^ a finding which raises the possibility that, in some circumstances, perturbed carbohydrate metabolism may confer a selective advantage.

We hope that the metabolically relevant link between the reactions of certain aldehydes and the N-terminal cysteine of ASNS identified here will promote further work to identify other biologically relevant but analytically challenging aldehyde- and alkylidene-derived products in cells, with those promoting tumorigenesis being of particular biomedical interest.

## Author contributions

T. J., C. J. S. and R. J. H. designed the study. T. J. conducted all experiments, except: NS conducted the THP-1 experiments, J. W.-T. and J. S. O. M. co-developed the metabolomics method, J. W. T. carried out/analysed metabolomics samples, F. A. D. conducted the mouse studies and C. L. M. homogenised the mouse samples. S. H. ran and analysed the protein MS/MS samples. T. J., C. J. S. and R. J. H. analysed the data and co-wrote the manuscript. All authors reviewed and commented on the manuscript.

## Conflicts of interest

There are no conflicts to declare.

## Supplementary Material

SC-015-D3SC06551K-s001
