# Supplementary material for: Aldehyde-mediated inhibition of asparagine biosynthesis has implications for diabetes and alcoholism
Source: Chem Sci. 2024 Jan 15;15(7):2509–17. doi: 10.1039/d3sc06551k (PMC10866355; doi:10.1039/d3sc06551k)
Supplement: SC-015-D3SC06551K-s001 [file SC-015-D3SC06551K-s001.pdf]

Electronic Supplementary Material (ESI) for Chemical Science.
This journal is © The Royal Society of Chemistry 2024

**Supplementary Information**

# Aldehyde-Mediated Inhibition of Asparagine Biosynthesis has Implications for Diabetes and Alcoholism

Tobias John[a], Nadia Saffoon[a], John Walsby-Tickle[a], Svenja S. Hester[b], Felix Dingler[c], Christopher L. Millington[c], James S. O. McCullagh[a], Ketan J. Patel[c], Richard J. Hopkinson[d*], and Christopher J. Schofield[a*]

[a]Chemistry Research Laboratory, 12 Mansfield Road, Oxford, OX1 3TA, United Kingdom. [b]Nuffield Department of Medicine, Target Discovery Institute, University of Oxford, Oxford, United Kingdom. [c]John Radcliffe Hospital/Headley Way, Oxford OX3 9DS, United Kingdom. [d]Leicester Institute for Structural and Chemical Biology and School of Chemistry, University of Leicester, Henry Wellcome Building, Lancaster Road, Leicester, LE1 7RH, United Kingdom.

E-mail: christopher.schofield@chem.ox.ac.uk or richard.hopkinson@leicester.ac.uk

## Reagents

Paraformaldehyde, DCDO, acetaldehyde ($CH_3CHO$), NaOD, DCl, N-methylmorpholine, *L*-glutathione (reduced), iodoacetamide acid and 1,3-cyclohexanedione were from Sigma-Aldrich. N6022 was from Generon Ltd. Further chemicals used were: H-*L*-Glu(OtBu)-OtBu*HCl (Iris Biotech), DTT (Apollo Scientific), *L*-Cysteine (Chem-Impex), EDC hydrochloride (Fluorochem), cycloheximide (Cayman Chemical, CAY-14126-1), Boc-Cys(Trt)-OH (Insight Biomtechnology Ltd), Boc-O-tert-butyl-serine-OH (Alfa Aesar), and Boc-Thr(tBu)-OH (Fluorochem Ltd).

## Characterisation Methods

NMR spectra (HSQC, HMBC, COSY, $^1H$, $^{13}C$) were obtained using either Bruker Avance AV700, AV600, AV500, or AV400 spectrometers. To reference spectra to the solvent peak, the following shifts were used: $\delta_H$ = $D_2O$ 4.79 ppm; $\delta_H$ = $D_6$-DMSO 2.5 ppm, $\delta_C$ = $D_6$-DMSO 39.52 ppm; $\delta_H$ = $CDCl_3$ 7.26 ppm, $\delta_C$ = $CDCl_3$ 77.16 ppm; $\delta_H$ MeOD = 3.31 ppm, $\delta_C$ = MeOD 49 ppm. For reporting multiplicities, the following abbreviations are used: m (multiplet), q (quartet), t (triplet), d (doublet), dd (doublet of doublets), s (singlet), brs (broad singlet), app (apparent). For $^1H$-$^{13}C$-HSQC and $^1H$-$^{13}C$-HMBC spectra; colour codes are as follows: red HSQC signals denote CH or $CH_3$, blue HSQC signals denote $CH_2$ coupling, and green signals denote HMBC coupling. Waters LCT Premier ESI mass spectrometer was used to obtain high resolution electron spray ionisation mass spectra (HRMS). LCMS Agilent Technologies 1200 series was used to obtain electrospray ionisation mass spectra.

## Solid-Phase Peptide Synthesis

A peptide corresponding to the *N*-terminus of *E. coli* ASNS B: $NH_2$-CSIFGVF-$NH_2$ (with a *C*-terminal amide) was synthesised (0.1 mmol scale) using a rink amide-MBHA resin (100–200 mesh, 0.6–0.8 mmol/g loading, AGTC Bioproducts) and a LibertyBlue microwave peptide synthesiser (CEM) as described.[1] *N*-α-Fmoc protected amino acids (from CS Bio,

Novabiochem, Sigma-Aldrich, TCI Chemicals, Alfa Aesar, Merck, and AGTC Bioproducts) and acid-labile protecting groups were employed. Coupling and deprotection steps used the instrument's standard methods and were microwave-assisted.

Amino acid solutions (0.2 M), dissolved in DMF (peptide synthesis grade, AGTC Bioproducts), were prepared. *N*,*N*'-Diisopropylcarbodiimide (TCI Europe) was used for coupling with Oxyma Pure. Fmoc deprotections employed 20% (v/v) piperidine in DMF. On synthesis of the peptide including *N*-terminal deprotection, the resin was washed three times with $CH_2Cl_2$, then dried in air. Acid labile protecting groups were removed; cleavage from the resin was conducted using 5 mL of a deprotection mixture (1,3-dimethoxybenzene (2.5%), triisopropylsilane (2.5%), Milli-Q (MQ) water (2.5%) in $CF_3CO_2H$ (92.5%)) for 4 h at ambient temperature. Upon filtration of the resulting mixture, the peptide was precipitated with ice-cold $Et_2O$ (45 mL). The solid was pelleted (4255 g, 10 min, 4.0 °C), the liquid was removed by decanting, and the resultant solid was dried in air. Before purification, the peptide was dissolved in $H_2O$ and lyophilised. After filtration (0.45 µm), purification was conducted using a Shimadzu HPLC purification system (DGU-20A, 2 LC-20AR, CBM-20A, SPD-20A, and FRC-10A units) equipped with a NX-C18 LC column (250 × 21.2 mm, 110 Å; Phenomenex Gemini). The mobile phase employed a gradient of MeCN + 0.1% (v/v) formic acid in $H_2O$ + 0.1% (v/v) formic acid. Upon HPLC purification, MALDI MS or LC-MS were used to analyse fractions. Fractions containing the product were pooled according to purity and lyophilised. The purified peptide was dissolved in $H_2O$ and its concentration determined using $^1H$ NMR (700 MHz) spectroscopy[2]: 16 µL of the $D_2O$ peptide solution were added to 143 µL $D_2O$ and 1 µL of 1 mg/mL 3-(trimethylsilyl)propionic-2,2,3,3-$d_4$ acid sodium salt (TSP; Apollo Scientific). The peptide concentration was calculated using: $\boldsymbol{M_x}/\boldsymbol{M_y}=(\boldsymbol{I_x}/\boldsymbol{I_y})\times(\boldsymbol{N_y}\boldsymbol{N_x})$, with $\boldsymbol{M_x}/\boldsymbol{M_y}$ representing the molar ratio of TSP and the peptide, $\boldsymbol{I_x}/\boldsymbol{I_y}$ the signal intensities corresponding to the methyl protons of TSP and the peptide (typically averaged over the Ala, Val, Thr, and Leu residues using Global Spectrum Deconvolution), and $\boldsymbol{N_y}/\boldsymbol{N_x}$ the number of nuclei responsible for the intensity.

**Dipeptide Synthesis**

See **Figure S28**.

**a) Boc- and tert-butyl-protected Dipeptides**

L-Glutamic acid di-tert-butyl ester hydrochloride (1.24 mmol) and the Boc-protected amino acid (Boc-Cys(Trt)-OH, Boc-O-tert-butyl-serine-OH, Boc-Thr(tBu)-OH; 1.24 mmol) were dissolved in 10 mL $CH_2Cl_2$ at 0 °C. *N*-Methylmorpholine (1.24 mmol) and EDC hydrochloride (1.24 mmol) were added and the mixture was allowed to warm to ambient temperature with stirring for 1 h. On completion of reaction, the mixture was quenched with 1 M HCl (aq) to give pH 7; the organic layer was separated and washed with 1 M NaCl, dried over $MgSO_4$ and the solvent removed. Flash column chromatography (0-20% MeOH in $CH_2Cl_2$ over 15 CV in 10 g $SiO_2$ column) afforded the protected dipeptides.

**Boc-*L*-Cys(Trt)-*L*-Glu(di-tBu-ester)**

$^{1}$H NMR (400 MHz, $CDCl_3$) $\delta_H$ 7.45 – 7.38 (m, 6H, 35, 37, 42, 44, 47, 49), 7.33 – 7.25 (m, 6H, 34, 38, 41, 45, 46, 50), 7.25 – 7.17 (m, 3H, 36, 43, 48), 6.61 (d, J = 8.0 Hz, 1H, 6), 4.80 (d, J = 7.5 Hz, 1H, 1), 4.40 (dt, J = 8.0, 5.0 Hz, 1H, 8), 3.85 (brs, 1H, 2), 2.81 – 2.69 (app. dd, m, 1H, 4'), 2.49 (dd, J = 13.0, 5.0 Hz, 1H, 4"), 2.35 – 2.17 (m, 2H, 11), 2.16 – 2.05 (m, 1H, 10'), 1.92 – 1.79 (m, 1H, 10"), 1.43 (s, 9H, 29, 30, 31), 1.42 (s, 9H, 18, 19, 20), 1.40 (s, 9H, 22, 23, 24); $^{13}$C NMR (101 MHz, $CDCl_3$) $\delta_C$ 172.2 (C12), 170.6 (C9), 170.3 (C3), 155.4 (C25), 144.5 (C33, C39, C40), 129.7 (C35, C37, C42, C44, C47, C49), 128.2 (C34, C38, C41, C45, C46, C50), 127.0 (C36, C43, C48), 82.4 (C17), 80.6 (C21), 77.4 (C28), 67.3 (C32), 53.7 (C2), 52.3 (C8), 33.7 (C4), 31.4 (C11), 28.4 (C18, C19, C20), 28.2 (C29, C30, C31), 28.1 (C22, C23, C24), 28.0 (C10); HRMS (ESI+) $(M+H)^+$: 727.3387 (calculated for $C_{40}H_{52}N_2O_7SNa$), 727.3382 (observed). Yield: 644 mg, 0.91 mmol, 73%.

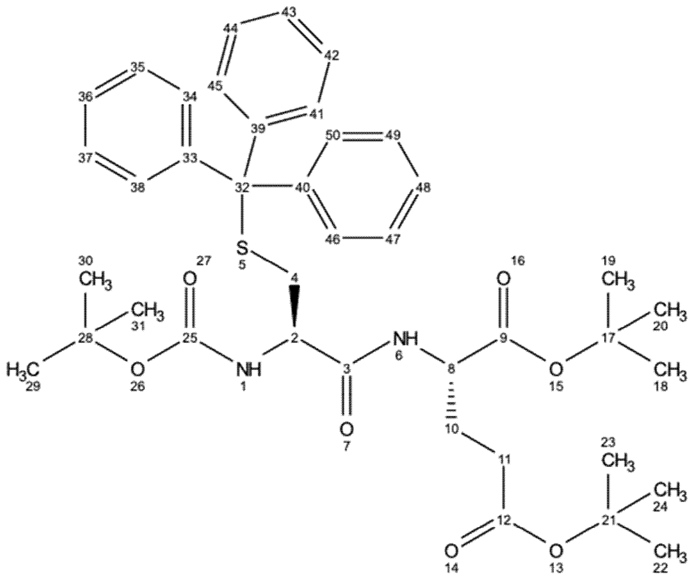

**Boc-*L*-Ser(tBu)-*L*-Glu(di-tBu-ester)**

$^{1}$H NMR (400 MHz, $CDCl_3$) $\delta_H$ 7.17 (brs, 1H, 5), 5.41 (brs, 1H, 1), 4.53 – 4.44 (m, 1H, 6), 4.15 (brs, 1H, 2), 3.81 (dd, J = 9.0, 3.5 Hz, 1H, 4'), 3.38 (app. t, J = 7.5 Hz, 1H, 4''), 2.36 – 2.08 (m, 3H, 10', 11), 1.92 – 1.80 (m, 1H, 10''), 1.45 (s, 18H, 21, 22, 23, 33, 34, 35), 1.43 (s, 9H, 25, 26, 27), 1.19 (s, 9H, 29, 30, 31); $^{13}$C NMR (101 MHz, $CDCl_3$) $\delta_C$ 172.2 (C12), 170.9 (C8), 170.6 (C3), 155.5 (C17), 82.3 (C32), 80.6 (C24), 80.2 (C20), 74.0 (C28), 62.0 (C4), 54.6 (C2), 52.2 (C6), 31.4 (C11), 28.5 (C33, C34, C35), 28.2 (C21, C22, C23), 28.1 (C10), 27.5 (C25, C26, C27), 27.1 (C29, C30, C31); HRMS (ESI+) $(M+H)^+$: 503.3327 (calculated for $C_{25}H_{47}N_2O_8$), 503.3326 (observed). Yield: 399 mg, 0.79 mmol, 64%.

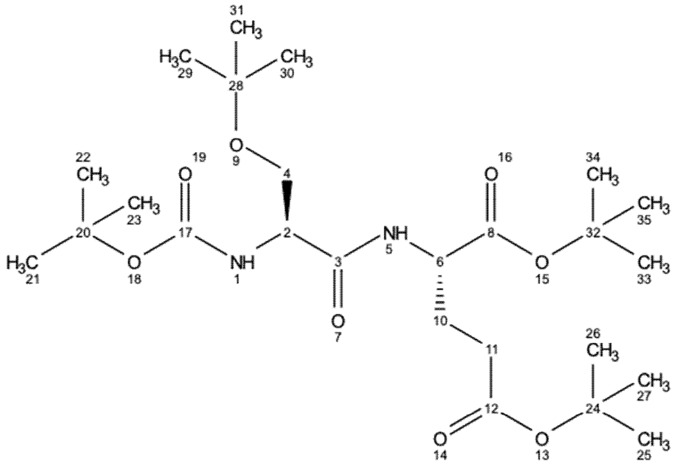

**Boc-*L*-Thr(tBu)-*L*-Glu(di-tBu-ester)**

$^{1}$H NMR (600 MHz, $CDCl_3$) $\delta_H$ 7.55 (d, J = 7.5 Hz, 1H, 5), 5.59 (d, J = 5.5 Hz, 1H, 1), 4.42 (dt, J = 7.5, 5.5 Hz, 1H, 6), 4.14 – 4.08 (m, 2H, 2, 4), 2.29 (ddd, J = 16.0, 10.0, 6.0 Hz, 1H, 11'), 2.22 (ddd, J = 16.0, 10.0, 6.0 Hz, 1H, 11''), 2.13 – 2.04 (m, 1H, 10'), 1.97 – 1.89 (m, 1H, 10''), 1.49 – 1.40 (m, 27H, 27, 28, 29), 1.26 (s, 9H, 19, 20, 21), 1.08 (d, J = 6.0 Hz, 3H, 17); $^{13}$C NMR (151 MHz, $CDCl_3$) $\delta_C$ 172.0 (C12), 170.6 (C8), 170.0 (C3), 155.7 (C30), 82.2 (C22), 80.7 (C26), 79.7 (C33), 75.3 (C18), 67.1 (C4), 58.6 (C2), 52.6 (C6), 31.6 (C11), 28.5 (C23, C24, C25), 28.2 (C34, C35, C36), 28.1 (C19, C20, C21), 27.8 (C10), 27.0 (C27, C28, C29), 17.4 (C17); HRMS (ESI+) $(M+H)^+$: 517.3483 (calculated for $C_{26}H_{49}N_2O_8$), 517.3484 (observed). Yield: 481 mg, 0.93 mmol, 83%.

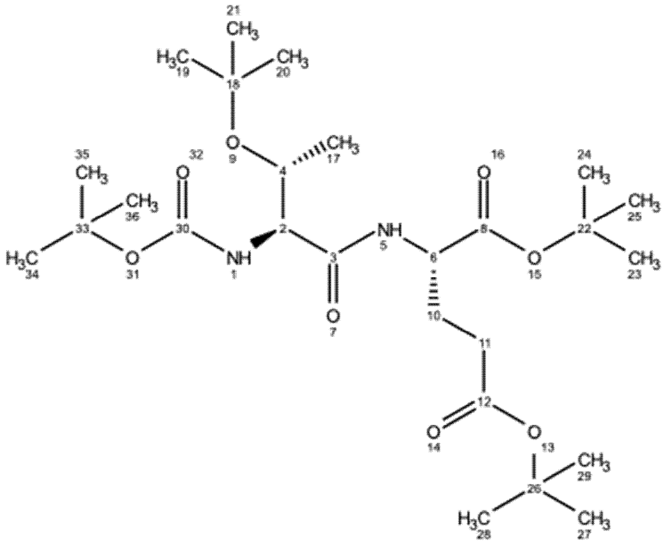

**b) Deprotection of Dipeptides**

The deprotection procedure was adapted from that of Polavckova et al.[3] The protected dipeptides were dissolved in $CH_2Cl_2$ and a 50-fold molar excess of $CF_3CO_2H$ was added. The mixture was stirred at ambient temperature for 2 h. On completion of the reaction, the mixture was evaporated to dryness and the oily residue evaporated three times with toluene. The residue was extracted with $H_2O$/EtOAc and the organic phase was washed with 1 M HCl. The aqueous layer was concentrated and purified by HPLC using an ACE5 C18 column (Hichrom, 100 x 21.2 mm) employing a 2% (v/v) isocratic elution with 2% (v/v) MeCN in $H_2O$, 0.1% (v/v) formic acid over 12 min. For the deprotection of Boc-*L*-Ser(Trt)-*L*-Glu(di-tBu-ester), the amount of $CF_3CO_2H$ used was increased to 70 equivalents with 6 equivalents of triisopropylsilane (40 °C).

***L*-Cys-*L*-Glu (3)**

$^{1}$H NMR (400 MHz, $D_2O$) $\delta_H$ 4.43 (dd, J = 9.0, 5.5 Hz, 1H, 8), 4.28 (t, J = 5.5 Hz, 1H, 2), 3.20 – 3.05 (m, 2H, 4), 2.56 – 2.48 (m, 2H, 11), 2.31 – 2.17 (m, 1H, 10''), 2.10 – 1.97 (m, 1H, 10'); $^{13}$C NMR (101 MHz, $D_2O$) $\delta_C$ 177.2 (C12), 175.3 (C9), 167.9 (C3), 54.1 (C2), 53.0 (C8), 30.2 (C11), 25.7 (C4), 25.0 (C10); ESI-MS, $[M+H]^+$: 251.07 (calculated), 251.10 (observed); HRMS (ESI+) $(M+H)^+$: 251.0696 (calculated for $C_8H_{15}N_2O_5S_1$), 251.0698 (observed). Yield: 25 mg, 0.1 mmol, 11%.

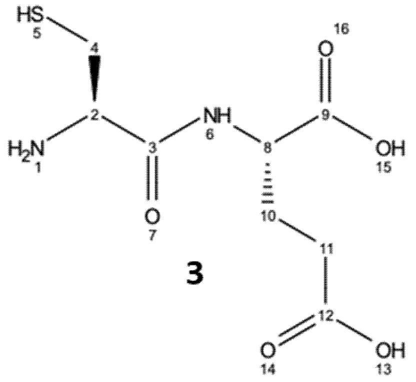


**3**

***L*-Ser-*L*-Glu (1)**

$^1$H NMR (400 MHz, $D_2O$) $\delta_H$ 4.49 (dd, J = 9.0, 5.5 Hz, 1H, 6), 4.18 (dd, J = 6.0, 4.0 Hz, 1H, 2), 4.05 (dd, J = 12.5, 4.0 Hz, 1H, 4'), 3.98 (dd, J = 12.5, 6.0 Hz, 1H, 4''), 2.56 – 2.45 (m, 2H, 11), 2.32 – 2.18 (m, 1H, 10'), 2.12 – 1.97 (m, 1H, 10''); $^{13}$C NMR (101 MHz, $D_2O$) $\delta_C$ 177.1 (C12), 174.9 (C8), 167.7 (C3), 60.1 (C4), 54.5 (C2), 52.6 (C6), 30.0 (C11), 25.9 (C10); ESI-MS, $[M+H]^+$: 235.09 (calculated), 235.15 (observed); HRMS (ESI-) $(M-H)^-$: 233.0779 (calculated for $C_8H_{13}N_2O_6$), 233.0778 (observed). Yield: 39.2 mg, 0.17 mmol, 21%.

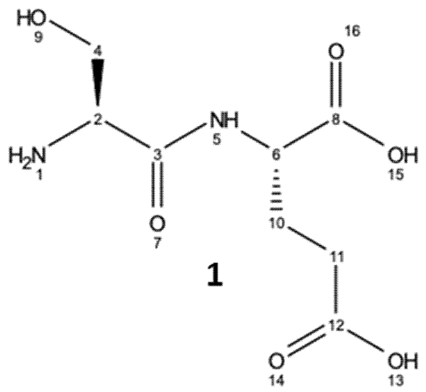


**1**

***L*-Thr-*L*-Glu (2)**

$^1$H NMR (400 MHz, $D_2O$) $\delta_H$ 4.40 (dd, J = 8.5, 5.5 Hz, 1H, 6), 4.09 (app. p, J = 6.5 Hz, 1H, 4), 3.81 (d, J = 6.5 Hz, 1H, 2), 2.44 (app. t, J = 7.2, 1.0 Hz, 2H, 11), 2.23 – 2.10 (m, 1H, 10''), 1.96 (ddt, J = 14.0, 8.5, 7.0 Hz, 1H, 10'), 1.27 (d, J = 6.5 Hz, 3H, 17); $^{13}$C NMR (101 MHz, $D_2O$) $\delta_C$ 177.1 (C12), 174.7 (C8), 167.9 (C3), 66.2 (C4), 58.6 (C2), 52.5 (C6), 30.0 (C11), 25.8 (C10), 18.7 (C17); ESI-MS, $[M+H]^+$: 249.11 (calculated), 249.16 (observed); HRMS (ESI+) $(M+H)^+$: 249.1081 (calculated for $C_9H_{17}N_2O_6$), 249.1082 (observed). Yield: 117 mg, 0.47 mmol, 51%.

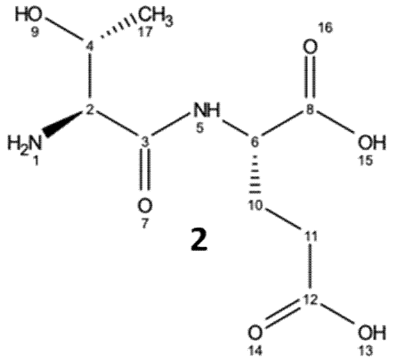


**2**

## c) Dipeptide Reactions with HCHO

Reactions of the dipeptides with HCHO (12 h, ambient temperature, $D_2O$) were studied by NMR ($^1$H NMR [600 or 400 MHz], $^{13}$C NMR [151 or 101 MHz] see below). In general, to obtain HCHO in solution, paraformaldehyde was resuspended in a microwave vial in the respective solvent (here $D_2O$) and heated at 95 °C for 3 h in a Biotage Initiator+ (0.5-2 mL vial, reference no. 352016, Biotage) with stirring to obtain a colourless solution. A 10-fold molar excess of aqueous HCHO (410 µmol) was added to the dipeptide solution (41 µmol); NaOD was added to obtain pH 9 (corresponding to pD 9.4). The solution was neutralized after 12 h, then subjected to HPLC purification (isocratic elution, 2% MeCN in $H_2O$, 0.1% (v/v) formic acid, 10 min, column ACE5 C18 (Hichrom), 100 x 21.2 mm), or studied by NMR. The product was concentrated to give a colourless solid. Note that for reactions with **1-3**, **3b** and **3c** were the only products sufficiently stable to undergo HPLC purification under the tested conditions. **3c** was prepared the same way as the HCHO adduct, but using a 10-fold excess of $CH_3CHO$ (pD 9.4, overnight).

### *L*-Cys-*L*-Glu HCHO adduct 3a, observed in situ

$^1$H NMR (600 MHz, $D_2O$) $\delta_H$ 4.40 (s, 2H, 18), 4.31 (d, J = 3.0 Hz, 1H, 1), 4.29 (d, J = 7.5 Hz, 1H, 16'), 4.23 (d, J = 10.0 Hz, 1H, 16"), 4.18 (m, 1H, 5), 3.34 (dd, J = 11.0, 3.0 Hz, 1H, 14'), 3.19 (dd, J = 11.0, 7.5 Hz, 1H, 14"), 2.24 (m, 2H, 8), 2.11 (m, 1H, 7"), 1.94 (m, 1H, 7'); $^{13}$C NMR (151 MHz, $D_2O$) $\delta_C$ 182.1 (C9), 178.5 (C6), 171.1 (C2), 78.1 (C18), 68.3 (C1), 55.3 (C5), 55.3 (C16), 34.1 (C8), 33.5 (C14), 28.4 (C7); ESI-MS, $[M+H]^+$: 263.07 (calculated), 264.10 (observed), corresponding to **3b**, likely resulting from fragmentation of **3a**.

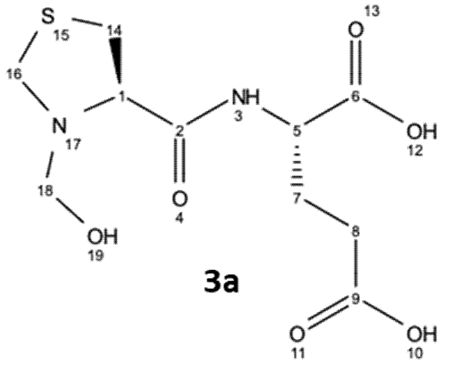


**3a**

**_L_-Cys-_L_-Glu HCHO adduct 3b, HPLC purified**

$^1$H NMR (600 MHz, $D_2O$) $\delta_H$ 4.66 (app. t, J = 7.0 Hz, 1H, 1), 4.50 (d, J = 10.0 Hz, 1H, 16''), 4.44 (d, J = 11.0 Hz, 1H, 16'), 4.35 (dd, J = 8.5, 5.0 Hz, 1H, 5), 3.60 (dd, J = 12.0, 7.5 Hz, 1H, 14''), 3.35 (dd, J = 12.0, 7.0 Hz, 1H, 14'), 2.49 (t, J = 7.5 Hz, 2H, 8), 2.26 – 2.18 (m, 1H, 7''), 2.08 – 1.99 (m, 1H, 7'); $^{13}$C NMR (151 MHz, $D_2O$) $\delta_C$ 177.7 (C9), 176.3 (C6), 167.5 (C2), 62.4 (C1), 54.2 (C5), 49.5 (C16), 33.5 (C14), 30.6 (C8), 26.3 (C7); ESI-MS, $[M+H]^+$: 263.07 (calculated), 263.10 (observed); HRMS (ESI+) $(M+H)^+$: 263.06962 (calculated for $C_9H_{15}N_2O_5S_1$), 263.06971 (observed). Yield from 20 mg starting material: 12 mg, 46 µmol, 57%.

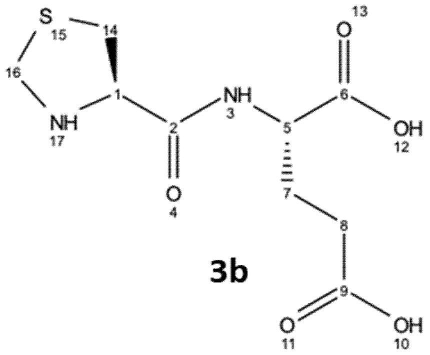


**3b**

**_L_-Cys-_L_-Glu acetaldehyde adduct (3c), HPLC purified, diastereomers A and B**

$^1$H NMR (600 MHz, $D_2O$) $\delta_H$ 5.02 (q, J = 6.5 Hz, 1H, 16, A), 4.97 (q, J = 6.5 Hz, 1H, 16, B), 4.66 (app. t, J = 7.5 Hz, 1H, 1, B), 4.41 – 4.34 (m, 5), 3.70 (dd, J = 12.5, 7.5 Hz, 1H, 14'', A), 3.65 (dd, J = 12.3, 7.5 Hz, 1H, 14'', B), 3.38 (dd, J = 12.5, 7.5 Hz, 1H, 14', A), 3.43 (dd, J = 12.5, 7.5 Hz, 1H, 14', B), 2.51 (t, J = 7.5 Hz, 8), 2.28 – 2.19 (m, 7''), 2.09 – 2.00 (m, 7'), 1.76 – 1.71 (m, 18), $^1$H resonance for 1 (A) is obscured under the residual solvent resonance; $^{13}$C NMR (151 MHz, $D_2O$) $\delta_C$ 177.6 (C9), 176.1 (C6), 167.5 (C2), 62.0 (C1, A), 62.7 (C1, B), 61.8 (C16, A), 61.5 (C16, B) 53.9 (C5), 33.5 (C14, A+B), 30.5 (C8), 26.2 (C7), 18.4 (C18, A), 17.3 (C18, B); ESI-MS, $[M+H]^+$: 277.09 (calculated), 277.106 (observed); HRMS (ESI+) $(M+H)^+$: 277.0853 (calculated for $C_{10}H_{17}N_2O_5S_1$), 277.0854 (observed). Yield from 20 mg starting material: 10 mg, 36 µmol, 45%; approx. diastereoisomer ratio A:B, ~56:44, respectively.

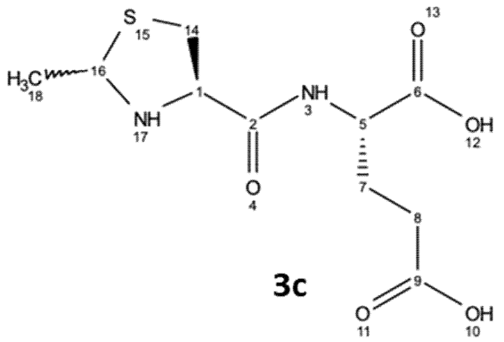


**3c**

***L*-Ser-*L*-Glu HCHO adduct, observed *in situ* (1a)**

$^{1}$H NMR (400 MHz, $D_2O$) $\delta_H$ 4.46 – 4.41 (m, 3H, 15’, 20), 4.19 – 4.12 (m, 2H, 2, 17'), 4.04 (dd, J = 8.5, 5.0 Hz, 1H, 19), 3.78 (dd, J = 8.5, 5.0 Hz, 1H, 17"), 2.24 – 2.14 (m, 2H, 5), 2.13 – 2.01 (m, 1H, 4"), 1.98 – 1.86 (m, 1H, 4'), $^{1}$H resonance for 15’’ is obscured under the residual solvent resonance; $^{13}$C NMR (101 MHz, $D_2O$) $\delta_C$ 181.9 (C6), 178.4 (C3), 173.8 (C14), 84.2 (C15), 77.1 (C20), 68.3 (C17), 62.6 (C13), 55.0 (C2), 34.0 (C5), 28.4 (C4); ESI-MS, $[M+H]^+$: not found, only starting material (**1**) was observed.

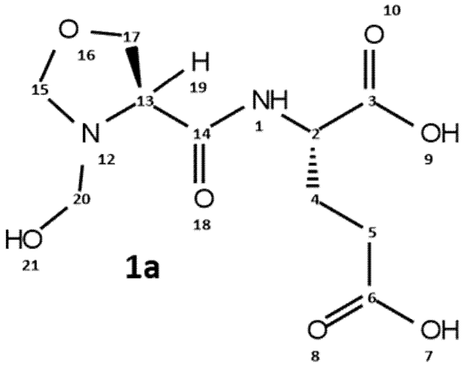

**1a**

***L*-Thr-*L*-Glu HCHO adduct, observed in situ (2a)**

$^{1}$H NMR (400 MHz, $D_2O$) $\delta_H$ 4.52 – 4.45 (m, 2H, 15', 11’’), 4.40 (d, J = 10.5 Hz, 1H, 11'), 4.18 (dd, J = 8.5, 4.5 Hz, 1H, 2), 4.01 (m, 1H, 17), 3.51 (d, J = 6.5 Hz, 1H, 19), 2.28 – 2.04 (m, 3H, 4’’, 5), 2.00 – 1.87 (m, 1H, 4'), 1.49 (d, J = 6.0 Hz, 3H, 20), $^{1}$H resonance for 15’’ is obscured under the residual solvent resonance; $^{13}$C NMR (101 MHz, $D_2O$) $\delta_C$ 182.0 (C6), 178.4 (C3), 173.5 (C14), 83.6 (C15), 78.2 (C17), 76.9 (C11), 68.4 (C13), 54.9 (C2), 34.0 (C5), 28.4 (C4), 18.7 (C20); ESI-MS, $[M+H]^+$: not found, only starting material (**2**) observed.

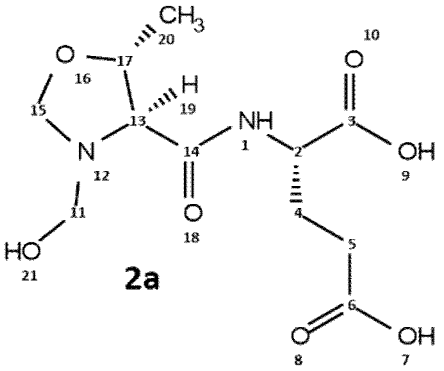

**2a**

CysSer (GenScript) was reacted with a 5-fold excess of HCHO in $D_2O$ for 8 h, then analysed by NMR and MS *in situ*.

$^1$H NMR (600 MHz, $D_2O$) $\delta_H$ 4.75 (app. t, J=1.2, 1H, 3), 4.55 (app. t, 1H, 9), 4.49 (app. dd, 2H, 1', 1''), 4.02 – 3.91 (m, 2H, 11', 11''), 3.64 (dd, J=7.6, 1.0, 1H, 4), 3.38 (dd, J=7.6, 1.0, 1H, 4); $^{13}$C NMR (151 MHz, $D_2O$) $\delta_C$ 173.5 (C10), 167.4 (C6), 62.1 (C3), 61.0 (C11), 55.9 (C9), 49.2 (C1), 33.4 (C4); HRMS (ESI-) (M-H)-: 219.0445 (calculated for $C_7H_{11}N_2O_4S_1$), 219.0438 (observed).

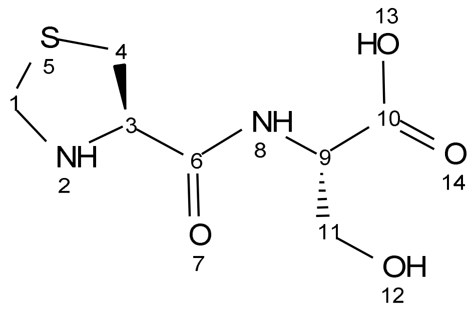


**Stability Studies With Peptides 1a, 2a and 3b**

A 40-fold molar excess GSH (from a 250 mM stock in $D_2O$) was added to *in situ* generated solutions of **1a** or **2a** or to HPLC purified **3b**. The reactions were monitored by NMR (400 or 600 MHz, see **Figure S17**).

**MALDI-TOF-MS Assays**

MALDI MS measurements employed a MALDI Autoflex Speed machine (Bruker). For preparation of the CHCA (α-cyano-4-hydroxycinnamic acid) matrix, CHCA was dissolved in MQ purified water/MeCN (1:1) 0.1% $CF_3CO_2H$ (1 mg CHCA per 100 µL mixture) and sonicated. The sample (1 µL) was then spotted with 1 µL of saturated CHCA solution onto a ground steel target (Bruker) and analysed. HCHO titration experiments were conducted at pH 7.4 in 5 µL potassium phosphate buffer (50 mM), 2.5 µM peptide concentration (2.5 µL from 10 µM stock in MQ water), 25 µM/250 µM HCHO (from 10 to $10^2$-fold excess; 2.5 µL from 100 µM/1 mM HCHO stock in MQ water). Reactions were performed in 10 µL final reaction volume in a 384 well plate (Microplate, 384 well, PP, V-bottom, 781280, Greiner) at ambient temperature, which was sealed (StarSeal Sealing Tape Polyolefin Film, E2796-9793, StarLab). Samples were taken after 1 min (directly after adding HCHO), 1 h, 2 h, 8 h and 24 h. No cross-links or further adducts were observed, but the peptide was partially present as disulfide-bridged dimer (data not shown).

## Cloning of DNA encoding for *E. coli* ASNS B

The open reading frame of *E. coli* ASNS B was from Thermo Fisher (GeneArt) and was codon-optimised for *E. coli* expression:

5'-
ATGTGCAGCATTTTTGGCGTGTTCGATATCAAAACCGATGCAGTTGAACTGCGTA
AAAAAGCACTGGAACTGAGCCGTCTGATGCGTCATCGTGGTCCGGATTGGAGCG
GTATTTATGCAAGCGATAATGCAATTCTGGCACATGAACGTCTGAGCATTGTTGA
TGTTAATGCCGGTGCACAGCCGCTGTATAATCAGCAGAAAACCCATGTTCTGGCA
GTGAATGGTGAAATCTATAATCATCAGGCACTGCGTGCAGAATATGGTGATCGTT
ATCAGTTTCAGACCGGTAGCGATTGTGAAGTTATTCTGGCCCTGTATCAAGAAAA
AGGTCCGGAATTTCTGGATGATCTGCAGGGTATGTTTGCATTTGCACTGTATGAT
AGCGAGAAAGATGCATATCTGATTGGTCGTGATCATCTGGGTATTATTCCTCTGT
ATATGGGCTATGATGAACATGGTCAGCTGTATGTTGCCAGCGAAATGAAAGCCC
TGGTTCCGGTTTGTCGTACCATTAAAGAATTTCCGGCAGGTAGCTATCTGTGGTC
ACAGGATGGCGAAATTCGTAGCTATTATCATCGTGATTGGTTCGATTATGACGCC
GTGAAAGATAATGTGACCGATAAAAATGAACTGCGCCAGGCACTGGAAGATAGC
GTTAAAAGCCATCTGATGAGTGATGTTCCGTATGGTGTTCTGCTGAGCGGTGGTC
TGGATAGCAGCATTATTAGCGCAATTACCAAAAAATACGCAGCCCGTCGTGTTG
AAGATCAAGAACGTAGCGAAGCATGGTGGCCTCAGCTGCATAGCTTTGCAGTTG
GTCTGCCTGGTAGTCCGGATCTGAAAGCAGCACAAGAAGTTGCAAACCATCTGG
GCACCGTTCATCATGAAATTCATTTTACCGTTCAAGAAGGTCTGGACGCAATTCG
TGATGTGATTTATCACATTGAAACCTATGATGTGACCACCATTCGTGCAAGCACC
CCGATGTATCTGATGTCACGTAAAATCAAAGCCATGGGCATTAAAATGGTTCTGA
GTGGTGAAGGTAGTGATGAAGTTTTTGGTGGCTATCTGTATTTTCACAAAGCACC
GAATGCAAAAGAACTGCATGAAGAAACCGTTCGTAAACTGCTGGCACTGCACAT
GTATGACTGTGCACGTGCCAATAAAGCAATGAGCGCATGGGGTGTTGAAGCACG
TGTTCCGTTTCTGGATAAAAAGTTCCTGGATGTTGCCATGCGTATTAATCCGCAG
GATAAAATGTGTGGCAATGGCAAAATGGAAAAACATATCCTGCGTGAATGCTTC
GAGGCATATCTGCCTGCAAGCGTTGCATGGCGTCAGAAAGAACAGTTTAGTGAT
GGTGTTGGTTATAGCTGGATTGATACCCTGAAAGAAGTGGCAGCACAGCAGGTT
AGCGATCAGCAGCTGGAAACCGCACGTTTTCGTTTTCCGTATAATACCCCGACCA
GCAAAGAAGCGTATCTGTATCGCGAAATTTTTGAAGAACTGTTTCCGCTGCCGAG
CGCAGCAGAATGTGTTCCTGGTGGTCCGAGCGTTGCCTGTAGCAGCGCAAAAGC
AATTGAATGGGATGAAGCCTTCAAAAAAATGGATGATCCGAGCGGTCGTGCCGT
TGGTGTTCATCAGAGCGCCTATAAATGA-3'

The ASNS B gene was amplified by PCR, introducing an NdeI restriction site at the 5' end and an XhoI restriction site at the 3' end, with an additional CCCGGG sequence adjacent to the restriction sites to increase efficiency of the restriction enzymes (forward primer 5'-CCCGGGCATATGTGCAGCATTTT-3', reverse primer 5'-CCCGGGCTCGAGTTTATAGGCGCTCTG-3', Merck). After digestion with NdeI and XhoI (overnight at 37 °C; New England Biolabs), the mixtures were purified (GeneJet Gel Extraction Kit, Thermo Fisher Scientific). The T4 DNA ligase (New England Biolabs) was used to ligate the fragments (2 days at ambient temperature). Following transformation of the ligation mixture into XL10-Gold cells (NEB, C3040H), the plasmid DNA of individual clones was isolated (Thermo Scientific GeneJet Plasmid Miniprep Kit) and the DNA sequence verified by sequencing (Eurofins).

**Preparation of *E. coli* ASNS B**

A glycerol stock of BL21(DE3) competent *E. coli* cells (NEB, C2527H) transformed with the plasmid pET22b ASNS B was used to inoculate a starter culture that was grown overnight at 37 °C and 180 rpm (+50 µg/mL ampicillin). The next day, 6 L of the main culture (containing 50 µg/mL ampicillin in 2xYT) was inoculated with 1:100 (v/v) of starter culture and grown until $OD_{600}$ of 0.85 at 37 °C and 150 rpm. 0.01 mM isopropyl ß-D-1-thiogalactopyranoside (IPTG) was added and growth continued overnight (18 °C, 150 rpm). Three drops of liquid polypropylene were used to remove foam to increase aeration. The cells were centrifuged (9820 g, 8 min, 4 °C) to yield a 70 g pellet which was frozen at -80 °C. The pellet (17.5 g) was resuspended in 70 mL Anion Exchange Buffer A (**Table S1**) (+1 protease inhibitor tablet [Sigma FAST EDTA free], +DNase I [Sigma]) at 4 °C. Cells were disrupted by 10 min sonication (Sonics Vibra-Cell) at 60% amplitude (10 s on, 10 s off, 5 min total sonication time). Cell debris was removed by centrifugation (63988 g, 30 min, 4 °C). The cell lysates were filtered using 0.45 µm filters (Filtropur S, Sarstedt). The filtrate was loaded onto a Q-Sepharose (8 mL) column employing the following salt gradient to purify *E. coli* ASNS B: The sample was applied at 2 mL/min; the column was washed with Anion Exchange Buffer A for 5 column volumes (CV); elution with 15%(v/v) Anion Exchange Buffer B (**Table S1**) for 2 column volumes (CV) was followed by a linear gradient from 15% to 100% (v/v) over 6 CV, and 6 CV of 100% buffer B. Fractions 16-55 were pooled and concentrated to 2 mL (Amicon Ultra-15, Centrifugal Filters, Ultracel 50 k, 4 °C, 3100 g). Subsequently, the concentrated protein was applied to a size exclusion column (S200 300 mL) using a flowrate of 0.5 mL/min. Proteins were eluted using 1.1 CV size exclusion buffer, and fractions 21-46 were pooled and concentrated (Amicon Ultra-15, Centrifugal Filters, Ultracel 50 k, 4 °C, 3100 g) to yield 1.2 mL of 91.1 mg/mL ASNS B (18.2 mg from 1 L of culture) (**Figure S29**). The concentrated protein solution was rapidly frozen and stored at -80 °C.

**ASNS B NMR Assays**

Buffers used are given in **Table S2**. ASNS B was diluted from the frozen stock solution (91.1 mg/mL) with size exclusion chromatography (SEC) buffer (50 mM BisTris, pH 7.5, 150 mM NaCl) to 1 mg/mL (15.7 µM). When ASNS B was preincubated with carbonyl compounds, the indicated molar excesses of carbonyl compound were added to 10 µL of 1 mg/mL ASNS B and incubated at 37 °C for 20 min (45 min for the detailed kinetic inhibition studies, with the NMR probe temperature at 37 °C during the kinetic study, **(Figure 3d**, **Figure S26d,e**; spectra were taken at 201 s intervals, with an acquisition time of 179 s). The preincubated enzyme/carbonyl compound mixture was added to the assay mixture containing ATP, Gln (Gln was not added in the experiment shown in **Figure 3b,** panel 5), Asp, $MgCl_2$, and $NH_4Cl$ ($NH_4Cl$ was only added for experiments shown in **Figure 3b,** panels 5-7). Assay mixtures were incubated in an Eppendorf tube at 37 °C for 4 h, quenched with 10 µL $CF_3CO_2H$, and frozen until analysis by $^1$H NMR (700 MHz). All other component stock solutions (ATP, Gln, Asp, $NH_4Cl$) were prepared in 50 mM potassium phosphate buffer in $D_2O$ (pH 7.6 corresponding to pD 8); only $MgCl_2$ was prepared in $D_2O$ due to solubility issues in potassium phosphate buffer; the pH of all components was adjusted to pH 7.6 (pD 8) using NaOD and DCl. Carbonyl compound solutions were prepared in MQ water without pH adjustment (glyoxylic acid was prepared in 50 mM potassium phosphate, pD 8 for the kinetic inhibition studies, **Figure 3d**, **Figure S26d+e**); the stock solutions of the carbonyl compounds were prepared such that 15.7 µL of the carbonyl compound solution was added to the ASNS B solution, independent of the

excess of the carbonyl compound used. For controls without ASNS B, 10 µL Size Exclusion Chromatography (SEC) buffer was preincubated with 15.7 µL MQ water; to assess activity without carbonyl compounds, ASNS B was preincubated with 15.7 µL MQ purified water.

### ASNS B Protein Observed Mass Spectrometry

For protein observed MS analyses, ASNS B was diluted with SEC buffer (**Table 1**) to 1 mg/mL, as for the NMR assays. 30 µl of 1 mg/mL enzyme stock was then reacted with 47.1 µL of carbonyl compounds (0.1, 1 or 10 mM stock in MQ water solution) at 37 °C for 20 min, which was then subjected to analysis by protein observed MS (**Figure S19**, **Figure S20**).

### Tissue Culture

Cells were from the American Type Culture Collection (ATCC; HEK293T, ref. CRL-3216; LN-18, ref. CRL-2610). Authentication from ATCC was relied upon. Cell lines were regularly tested to be mycoplasma negative (MycoAlert Mycoplasma Detection Kit ref. no LT07-218). Cell lines were cultured in DMEM (D6546, Sigma; note this formulation does not contain asparagine) supplemented with 10% FBS (Gibco) and 1% (2mM final concentration) GlutaMAX (Gibco) in a 37 °C incubator at 5% $CO_2$. For the experiments shown in **Figure 1a,b**, **Figure S2b** and **Figure S3**, dialysed FBS was used (A3382001, Thermo Fisher Scientific). For harvesting the cells, the medium was aspirated and cells were washed twice with phosphate buffered saline (PBS, Sigma, D8537). Upon aspirating PBS, radioimmunoprecipitation assay buffer (1x RIPA [Sigma, R0278], supplemented with protease inhibitor [cOmplete™, Mini, EDTA-free Protease Inhibitor Cocktail, Roche]), was used to scrape the cells. The cell suspension in the reaction tube was either frozen at -20 °C or incubated for 45 min on ice and vortexed several times during that time interval. After centrifuging (16,000 g, 15 min, 4 °C), the supernatant was used for analyses.

### THP-1 Proliferation Assays

For cell proliferation assays (**Figure 3e and S27)**), THP-1 cells were plated in technical triplicates at a density of $0.15*10^6$ cells/well into 96-well plates (100 µl/well) (Sarstedt AG &Co.KG, Costar-83.3924) using RPMI-1640 media (Gibco Life Technologies, 21875-034) with 10% FBS (Sigma–Aldrich, F7524) and 1% penicillin/streptomycin (Gibco Life Technologies, P4333). Cells were maintained at 37 ºC in a 5% $CO_2$ for 72 h. L-asparaginase (0.05 U/ml), HCHO (80 µM) and N6022 (10 µM) were added on day one 24 hours after seeding the cells. 40 µM HCHO and 10 µM N6022 were added to THP-1 cells at 48 h after seeding the cells. 20 µl of CellTiter 96® AQueous One Solution (Promega, G358C) were added to each well to determine the number of viable cells at 72 h after seeding the cells. GraphPad Prism version 9.5.1 (GraphPad Software, Inc.) was used to determine the relative viability compared to non-treated THP-1 cells (i.e. no L-asparaginase, no HCHO, and no N6022).

### SDS-PAGE Analyses

Polyacrylamide gel electrophoresis was performed using NuPAGE 4-12% Bis-Tris Protein Gels (Life Tech) in 1x MOPS running buffer (20x NuPAGE MOPS SDS Running Buffer, NP0001). SDS-PAGE was run at 170 V for 40 min (Mini Gel Tank, Life Technologies). The PageRuler Prestained Protein Ladder (Thermo Fisher Scientific, reference 26617) was used.

**Western Blot Analyses**

Proteins resolved by SDS-PAGE were transferred onto a nitrocellulose membrane (Amersham Protran Premium 0.2 NC 300 mm, GE Healthcare) using a current of 350 mA for 60-90 min or 85 mA overnight (Mini Protean Tetra Cell, Bio-Rad) in transfer buffer containing 10% (v/v) methanol (20x NuPAGE Transfer Buffer, Invitrogen). Membranes were blocked in 5% milk (w/v) 1x PBS 1‰ tween (PBST; Tween-20, Sigma-Aldrich) at ambient temperature for at least 30 min. The membranes were then incubated with the appropriate primary antibody (1:1000) in 5% milk (w/v) PBST at 4 °C overnight on a shaker. After three 10-min washes with PBST, the blots were incubated with the appropriate secondary antibody (in 5% milk PBST in a 1:5,000 dilution, 1 h, ambient temperature) followed by detection using GE Healthcare Amersham ECL Prime Western Blotting Detection Reagent (RPN2236) and Bio-Rad Universal Hood iii. Primary antibodies used were: GAPDH Loading Control Monoclonal Antibody (GA1R, Invitrogen, MA5-15738), abcam Asparagine Synthetase Antibody (ab226413; used for immunoprecipitation only), Origene ASNS antibody (TA503444; used for mouse samples only), and Cell Signaling Asparagine Synthetase Antibody (ref. no. 20843). Secondary antibodies used were anti-mouse IgG, HRP-linked Antibody (Cell Signaling, reference 7076), anti-Rabbit IgG (H+L) HRP Conjugate (Promega reference W4011). Raw images for western blots are given in Figure S30.

**RNA-Seq Analyses**

HEK293T cells (0.9 x$10^6$) were seeded into 6 cm dishes as biological triplicates. The next day, cells were treated with 150 µM HCHO (or MQ water control), followed by another cumulative treatment of 225 µM HCHO (or MQ water control) the subsequent day. Cells were harvested 4 h after the second treatment. For harvesting, cells were washed twice with ice-cold PBS (Sigma, D8537); RNA was isolated using the PureLink™ RNA Mini Kit (Invitrogen, 12183020) following the manufacturer's recommendations on "purification from plant and animal cells", "monolayer cells", "syringe homogenization", and "on-column DNase treatment" (PureLink DNase Set, Invitrogen, 12185010). Samples were sent for RNA-Seq PolyA to the Oxford Genomics Centre.

For transcript-analysis, reads were mapped for each sample to the reference genome (ftp.ensembl.org/pub/release-77/gtf/homo_sapiens/Homo_sapiens.GRCh38.77.gtf.gz) using HISAT[65] and the resulting SAM files were sorted and converted to BAM files using SAMtools.[66] The resulting BAM files were merged with Stringtie[64] and transcript abundances were estimated and table counts created for Ballgown[65] using the differential expression analysis protocol described by Pertea et al.[66]

**Cell Viability Assays**

1*$10^4$ HEK293T cells were seeded in a 96-well plate. After the cells were attached (>8 h), they were treated with a first dose of HCHO, followed by a second dose of HCHO after 24 h. The second dose was 1.5-fold higher than the first dose. Thus, e.g. the data point at 200 µM HCHO represents an initial dose of 80 µM HCHO, followed by 120 µM HCHO (administered 24 h after the first dose). 14 h after the second dose, viable cells were estimated (**Figure S4c**) using CellTiter 96® AQueous One Solution Reagent (Promega, G358C).

**Mouse Studies**

All animal experiments undertaken in this study were done so with the approval of the University of Oxford's Animal Welfare Ethical Review Body and under project license authority granted by the UK Home Office under the Animal (Scientific Procedures) Act 1986.

*Adh5*$^{-/-}$ mice were generated using the previously described *Adh5*$^{tm1Stam}$ allele (MGI ID: 3033711),[67] a gift from Limin Liu, and maintained in a C57BL/6J background, as were *Aldh2*$^{-/-}$ mice, based on the *Aldh2*$^{tm1a(EUCOMM)Wtsi}$ allele (MGI ID: 4431566) [REF:22, Langevin et al., 2011]. Age- and sex-matched C57BL/6J mice (Charles River) served as controls. All animals were maintained in specific pathogen-free conditions on a 12-hour light/dark cycle in individually-ventilated cages with environmental enrichment. Mice had access to drinking water and food (Teklad Global 16% Protein Rodent Diet) *ad libitum*.

For the methanol challenge experiment, mice received a single injection of 19 mL/kg body weight of 20% (v/v) methanol in physiological saline solution, and were sacrificed 6 h later. Samples were lysed in a 2 mL Eppendorf tube with a 7 mm stainless steel metal ball (Qiagen). Samples where homogenized in a tissue lyser (Qiagen/Retsch) for 4 min at 30 Hz. For Western blots, 10-20 mg of tissue was cut and lysed in 200 µL of RIPA buffer (supplemented with protease inhibitor). For the metabolomics samples, 20-40 mg of liver tissue was lysed in 400 µl 80% MeOH (v/v).

For the ethanol challenge experiment, mice were injected intraperitoneally with 26 mL/kg of a 20% (v/v) solution of ethanol in saline, split into two doses of 13 mL/kg, 4 hours apart. Tissues were collected 16 hours after the first injection.

**Metabolomics Mass Spectrometry**

For cell harvesting, media was removed from the flask by decanting; the cells were then washed twice with PBS buffer to remove residual buffer and dead cells (Sigma, D8537) (residual PBS was removed). Cell metabolism was arrested by adding liquid $N_2$ cover cells; 200 µL of 80% (v/v) MeOH 20% (v/v) MQ water was added to extract metabolites and a cell scraper used to de-adhere all cells from surface of the container. The MeOH/cell debris were removed into a centrifuge tube and centrifuged (18000 g, 25 min, 4 °C). For normalizing each experimental condition, the dsDNA concentration of the supernatant was measured. The supernatant was passed through centrifugal filters that had been pre-washed with MQ water (Amicon Ultra Centrifugal Filters, 10 kDa Ultracel, 0.5 mL; 18000 g for 25 min at 4 °C). The filtrate of each experimental condition was diluted with 80% (v/v) MeOH 20% (v/v) MQ water according to the dsDNA factor to normalise sample volume to DNA content (based on the lowest dsDNA concentration obtained). Next, the AccQ-Tag Ultra Derivatization Kit (Waters, Elstree, UK. ref. 186003836) was added to a 5 µL aliquot of the sample in order to derivatize amino acids, following the manufacturer's recommendations. Targeted metabolomic analyses were performed for the analysis of free amino acids and other derivatised primary and secondary amines. Reversed-phase chromatography was performed using an ACQUITY I-Class PLUS UPLC System (Waters, Milford, MA, USA) coupled to a Vion IMS QTof mass spectrometer (Waters, Milford, MA, USA). An AccQ-Tag Ultra C18 column (2.1x100 mm, 1.7 µm; Waters, Milford, MA, USA) at 50 °C was used with mobile phase A: water with 10.0 % Eluent A concentrate (Waters, Milford, MA, USA), and mobile phase B: MeCN with 1.3% formic acid. The linear gradient used was: 0.00 min, 0.1 % B; 0.54 min, 9.1% B; 5.74 min 21.2% B; 7.74 min, 59.6% B; 8.04 min, 90.0% B; 8.05 min, 90.0% B; 8.64 min, 0.1% B; and

9.50 min, 0.1% B (v/v). The flow rate was 0.50 mL/min and the total analysed time was 9.50 min. The Vion IMS QTof spectrometer was equipped with an ESI probe in the positive ion mode with source parameters as follows: capillary voltage, +2.0 kV; cone voltage, 40 V; source temperature, 130 °C; desolvation temperature, 450 °C; cone gas flow, 50 l/h; and desolvation gas flow, 900 L/h. Scan parameters were as follows: analyser mode, sensitivity High Definition $MS^E$; scan range, 501000 *m/z*; scan time, 0.200 s; low energy, 6.0 eV; and high energy ramp, 20.030.0 eV. Lockmass data were collected every 5.0 min using leucine enkephalin and used for accurate mass correction. Raw data files were processed using Progenesis QI (Waters Corp, Estree, UK) with retention time (RT) alignment, and peak picking was performed for relative abundance comparisons. After processing, metabolites were identified by meeting the following criteria: accurate mass measurement (within 5 ppm), RT (within 2 min of database values), isotopic distribution, fragmentation pattern matching (greater than 25 % similarity, where available), and coefficient of variation (less than 35 % for QC samples). Data sets were normalised using MetaboAnalyst.[68] Statistical significance was assessed using GraphPad Prism 5 (Version 5.04) using 1way ANOVA with Dunnett's Multiple Comparison Test or an unpaired t-test (if only two experimental groups were present); ****P≤0.0001, ***P≤0.001, **P≤0.01, *P≤0.05, ns: not significant.

**Immunoprecipitation**

Immunoprecipitations were performed using Dynabeads™ Protein G for Immunoprecipitation (Invitrogen), by adapting the manufacturer's methods. In brief, the Dynabeads were vortexed for 30 s to resuspend the magnetic beads. The bead suspension (50 µL) was transferred to a 1.5 mL Eppendorf tube, which was placed on a magnet and the supernatant was removed. The tube was removed from the magnet. The asparagine synthetase antibody (7.5 µg; from Abcam (ab226413)), diluted in 200 µL PBS (phosphate buffered saline, with 0.02% Tween 20) was added to the magnetic beads and incubated with rotation (10 min, ambient temperature). The tube was placed on the magnet and the supernatant removed. The tube was removed from the magnet and the beads were washed with 200 µL PBS (0.02% Tween 20) by gentle pipetting. The tube was placed on the magnet and the supernatant removed. 2 mg of the radioimmunoprecipitation assay buffer (RIPA) lysed sample containing the antigen diluted with 200 µL PBS (0.02% Tween 20) was added to the bead-antibody complex which was resuspended by gentle pipetting. This mixture was incubated with rotation (2 h, ambient temperature). The tube was then placed on the magnet and the supernatant was transferred to a new tube for analysing the binding efficiency of the antigen. The bead-antibody-antigen complex was washed 3 times with 200 µL PBS (without Tween 20); the supernatant was removed with the aid of the magnet after each wash. The bead-antibody-antigen complex was resuspended using 100 µL PBS (without Tween 20) and transferred to a new tube to prevent co-elution of proteins attached to the tube. The tube was placed on the magnet to remove the supernatant. 20 µL 50 mM glycine pH 2.8 and 10 µL of 2x NuPAGE™ LDS Sample buffer containing dithiothreitol (DTT, dissolved in 100 µL LDS Sample buffer 4x to 0.04 M, which was then diluted 1:1 with MQ water to result in a 2x sample buffer) was used to resuspend the bead-antibody-antigen complex. The antibody-antigen complex was released from the magnetic beads by heating the sample for 10 min at 70 °C. The tube was placed on a magnet and the supernatant was used for western blot analysis and tryptic digestions.

**In-solution Trypsin Digestion**

After immunoprecipitation, the antigen mixture was used for trypsin hydrolysis after confirming the presence of the antigen by western blot analysis (**Figure S24a**). The sample was treated with dithiothreitol (DTT) (2 µL of 85 mM DTT in 50 mM ammonium bicarbonate, 40 min, 56 °C), then alkylated with iodoacetamide (IAA) (7 µL of 55 mM IAA in MQ water for 30 min in the dark at ambient temperature). Excess IAA was removed by treatment with 3 µL of 85 mM DTT in 50 mM ammonium bicarbonate (10 min in the dark at ambient temperature). Six volumes of ice-cold acetone were added to induce precipitation, whilst vortexing several times, followed by storage overnight at -20 °C. The sample was then centrifuged (15000 g, 10 min, 4 °C), the supernatant removed, the pellet was dried (5 min) and resolubilized with 27 µL 50 mM ammonium bicarbonate. Digestion was performed using Pierce Trypsin Protease, MS Grade. The lyophilized trypsin pellet (20 µg) was reconstituted in 20 µL of 50 mM acetic acid and 60 µL of 50 mM ammonium bicarbonate, resulting in a 0.25 µg/µL stock solution. The trypsin stock solution (3 µL) was added to the sample, which was incubated overnight at 37 °C. To promote digestion in folded parts of the protein, another digestion step was performed in 80 (v/v) % acetonitrile; thus, 1 µL of trypsin stock solution and 124 µL MeCN were added to the sample, followed by incubation at 37 °C for 3 hours. Digestion was stopped by adding 5% (v/v) formic acid and the sample dried using vacuum centrifugation (Eppendorf Concentrator Plus, >2 h, 30 °C, 1400 rpm with rotor F-45-48-11, V-AQ mode). The pellet was resolubilized in 20 µL 0.1% (v/v) formic acid, 98% (v/v) MQ water, 2% (v/v) MeCN and analysed using a LC-MS/MS Orbitrap Elite mass spectrometer.

**MS/MS fragmentation studies**

Lyophilised peptides were reconstituted in 5% (v/v) DMSO and 5% (v/v) formic acid, then analysed by liquid chromatography tandem mass spectrometry (LC-MS/MS) using an Ultimate 3000 UHPLC (ThermoFisher Scientific) machine connected to an Orbitrap Fusion Lumos Tribrid machine (ThermoFisher Scientific). The peptide solutions were loaded onto a PepMapC18 column (300 µm x 5mm, 5µm particle size, Thermo Fischer), then separated using a 50 cm-long EasySpray column (ES803, Thermo Fischer) with a gradient of 2-35% (v/v) MeCN in 5% (v/v) DMSO, 0.1% (v/v) aqueous formic acid (250 nL/min flow rate over 60 min). Eluted peptides were analysed using an Orbitrap Fusion Lumos Tribrid platform (instrument control software v3.3). Data were acquired in the data-dependent mode, with advance peak detection (APD) enabled. Survey scans were acquired in the Orbitrap at 120 k resolution over a m/z range of 400 -1500, Automatic Gain Control (AGC) target of 4e5 and S-lens radio frequency (RF) of 30. Precursor ions were isolated in the Quadrupole (1.6 isolation window), fragmented in the Higher-energy C-trap dissociation (HCD) cell with 30% capillary electrophoresis (CE), and analysed in the Orbitrap at 30K resolution with an AGC target of 5e4 and 54 ms maximum injection time and a 7 sec dynamic exclusion list.

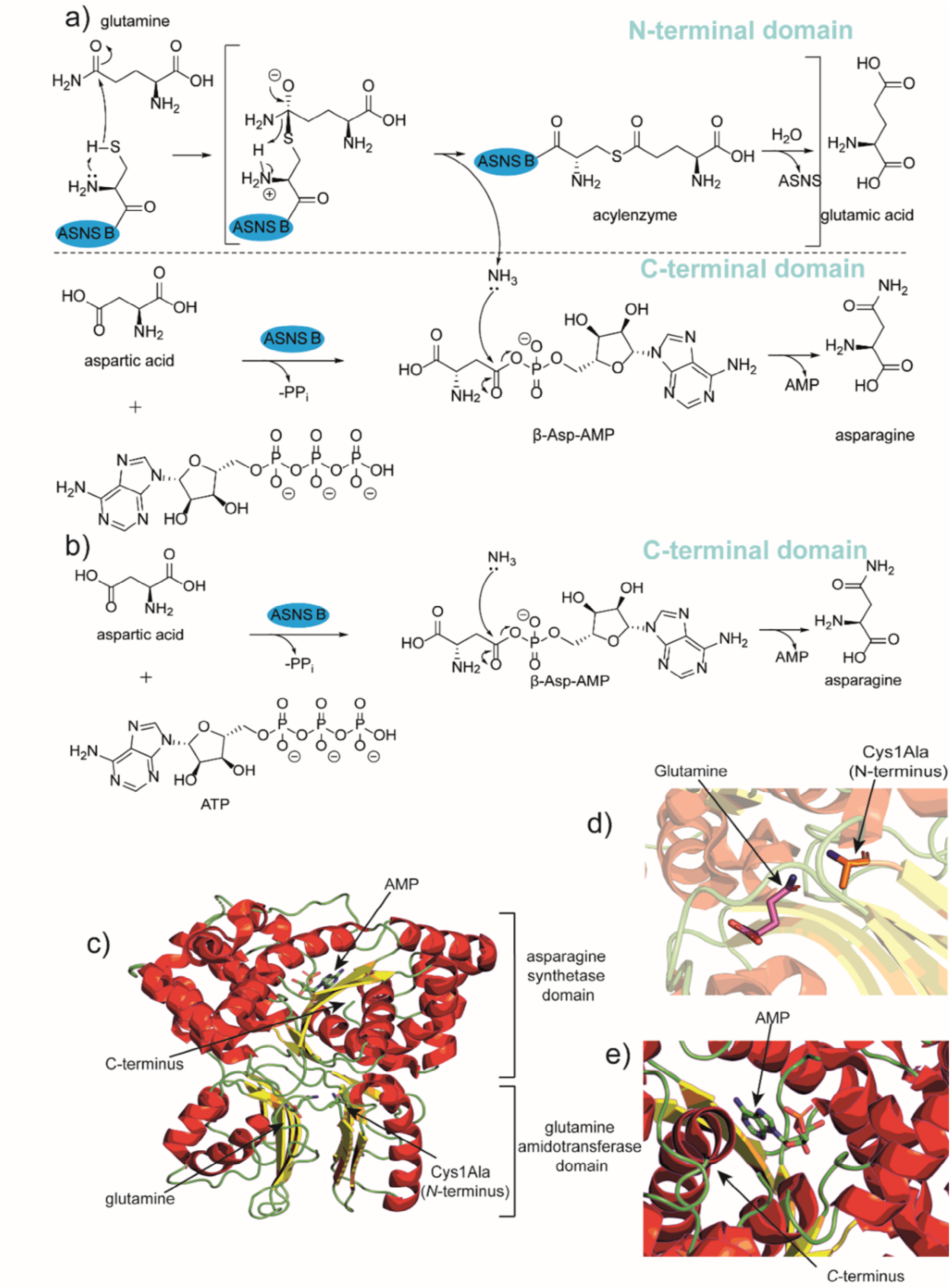


**Figure S1 | Outline mechanism and structure of ASNS. a,b.** The nucleophilic *N*-terminal cysteine of *E. coli* asparagine synthetase B (ASNS B) and human ASNS reacts with glutamine to form an acyl-enzyme complex, which is hydrolysed forming glutamic acid and ammonia. The ammonia then travels from the glutaminase domain into the synthetase domain, where it reacts with β-aspartyl-adenosine monophosphate (β-Asp-AMP) to form asparagine and adenosine monophosphate (AMP). **b.** The C-terminal domain catalyses production of asparagine from aspartate, ATP and $NH_3$. **c,d,e.** Views from a crystal structure of ASNS B (PDB: 1CT9)[4] highlighting the *N*-terminal glutamine amidotransferase domain with glutamine in the binding pocket and the asparagine synthetase domain with AMP in the binding pocket.

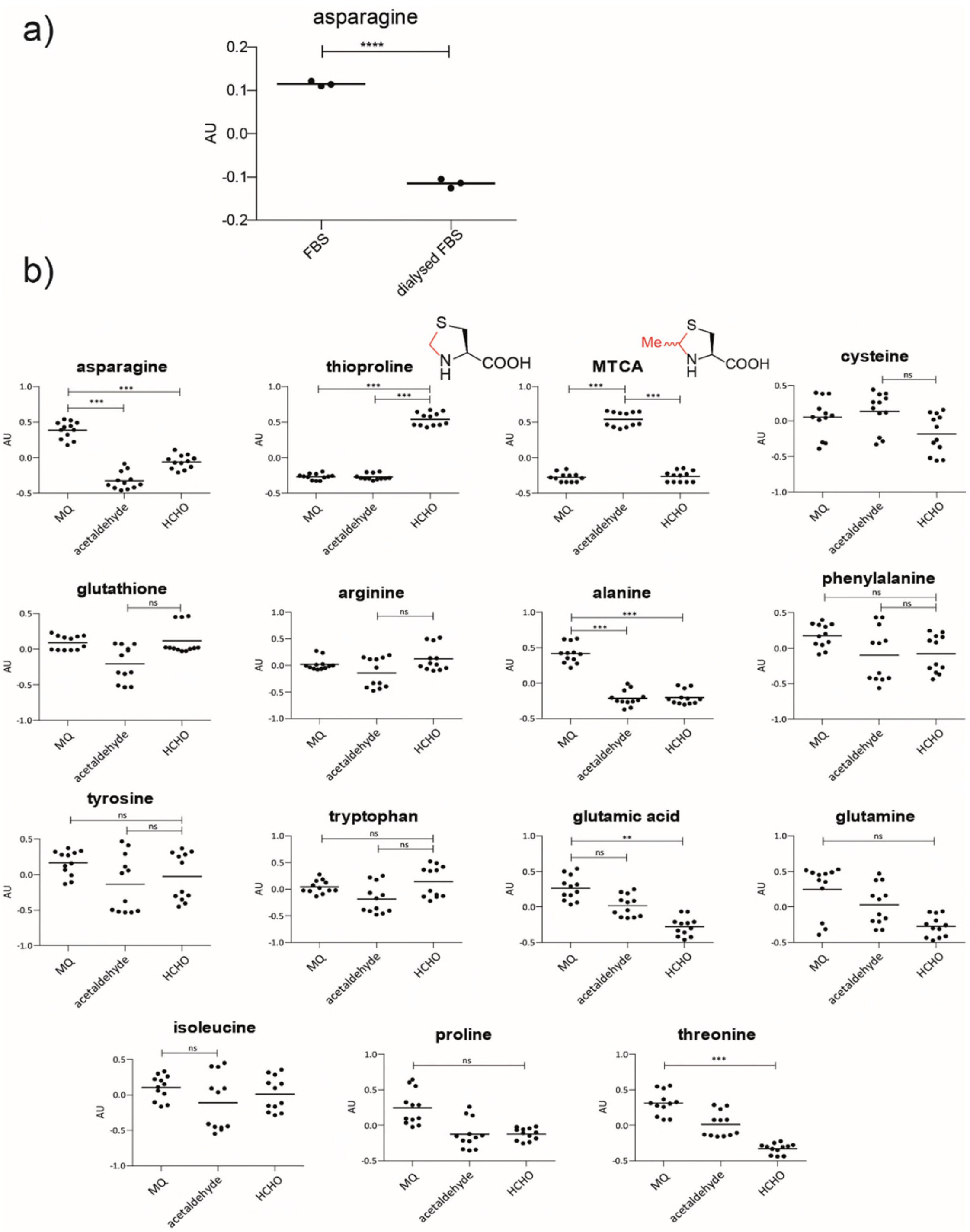


**Figure S2 | Metabolomics analyses of aldehyde-treated HEK293T cells grown in the presence of dialysed fetal bovine serum. a.** Asparagine levels in growth medium (DMEM, FBS, GlutaMAX) compared to DMEM supplemented with dialysed fetal bovine serum (FBS). Instrumental analyses in triplicate are shown. **b.** HEK293T cells were seeded in growth medium (DMEM, dialysed FBS, no glutamine/GlutaMAX). After 8 h, MQ water, HCHO (200 µM) or $CH_3CHO$ (2 mM) were added. All experimental groups were supplemented with 2 mM glutamine. After 15 h, the same amounts of aldehydes or MQ water were added. Cells were harvested after a further 8 h. Individual data points are shown as means of biological quadruplicates ran as instrumental triplicates (12 data points per group). Statistical significance was assessed based on the mean of instrumental triplicates.

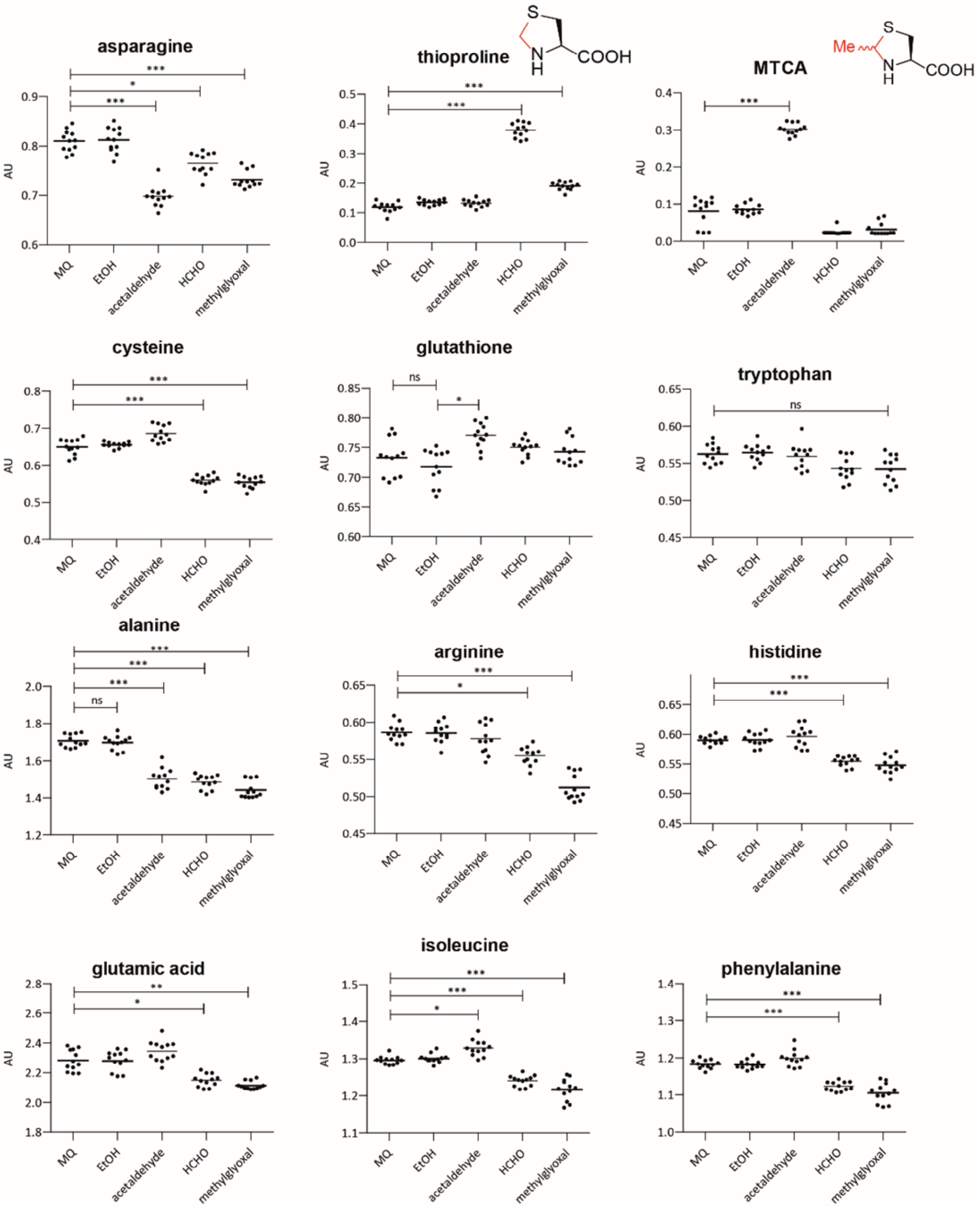


**Figure S3 | Metabolomics analyses of aldehyde treated HEK293T cells grown in the presence of dialysed fetal bovine serum.** Full data set of results shown in **Figure 1a,b**. HEK293T cells were seeded in growth medium (DMEM, dialysed FBS, no glutamine/GlutaMAX). After 7.5 h, MQ water, ethanol (EtOH, 17 mM), $CH_3CHO$ (2 mM), HCHO (150 µM) or methylglyoxal (750 µM) were added. All experimental groups were supplemented with glutamine (2 mM). After 15.5 h, the same amounts of aldehyde or MQ water were added (with the exception of methylglyoxal, where 250 µM were added). Cells were harvested after a further 8 h. Individual data points are shown as means of biological quadruplicates ran as instrumental triplicates (12 data points per group). Statistical significance was assessed based on the average of the instrumental triplicates. The GSH-aldehyde adducts and the cysteine-methylglyoxal adduct could not be detected under our conditions. Note, commercial methylglyoxal is reportedly contaminated with up to 7% HCHO,[5] which might explain the slight increase in thioproline in the methylglyoxal conditions.

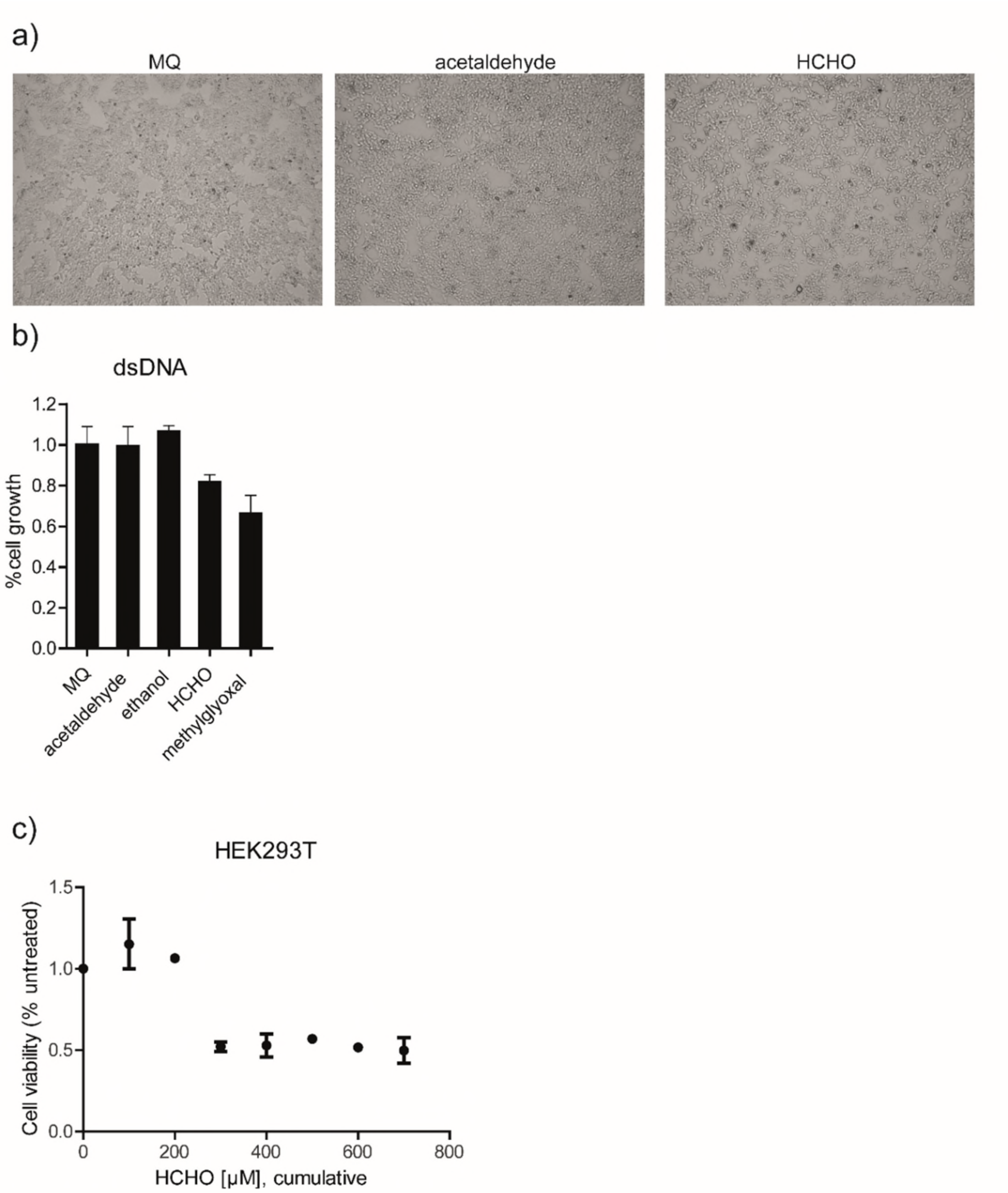


**Figure S4 | Cellular analyses following aldehyde or ethanol treatment. a.** Representative images of HEK293T cells before harvesting for the MS analyses shown in **Figure S2b**. Little evidence was observed for toxicity or morphology changes on $CH_3CHO$ (2 doses of 2 mM) or HCHO treatment (2 doses of 200 µM). **b.** Double stranded (ds) DNA concentration as a proxy for cell number (as measured by Nanodrop One, Thermo Scientific) of HEK293T cells on treatment with the indicated compounds. The HCHO (2 doses of 150 µM) and methylglyoxal (750+250 µM) treatments resulted in reduced cell growth, whereas $CH_3CHO$ (2 doses of 2 mM) and ethanol (2 doses of 17 mM) addition did not. Errors: SD (n = 4, samples were used for metabolomics experiment shown in **Figure S3**). **c.** HEK293T cells were treated with two cumulative doses (that is without exchanging the medium between the two treatments) of HCHO 24 h apart. After 14 h post the second treatment, viable cells were estimated using the CellTiter 96® AQueous One Solution Reagent. Errors: SEM (n = 3, technical repeats).

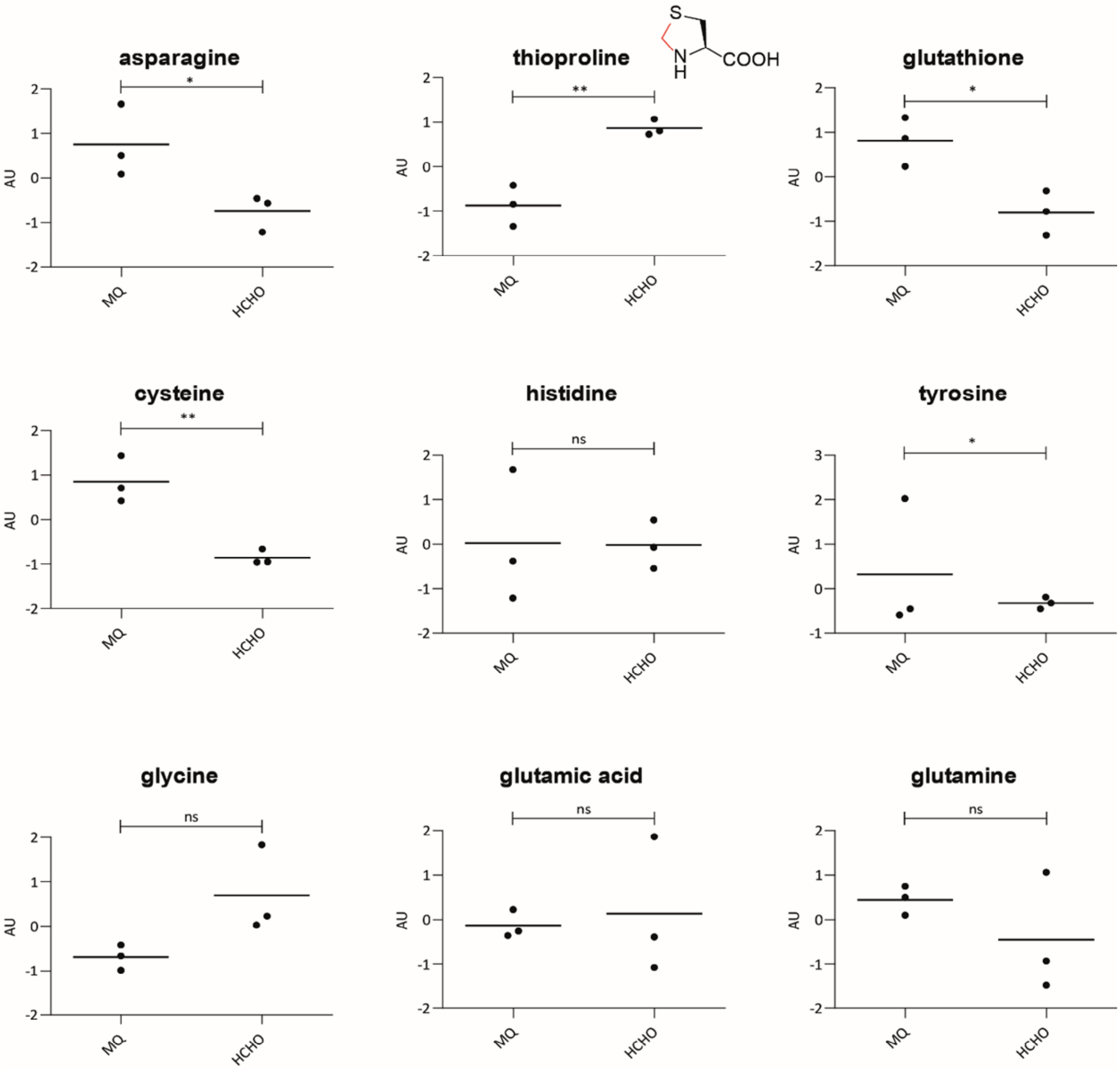


**Figure S5 | Metabolomics analyses of HCHO-treated LN-18 cells.** LN-18 cells (grown using non-dialysed FBS) which were treated with HCHO (180 µM) for 24 h, followed by a second treatment at 270 µM. Cells were harvested 26 h after the second treatment.

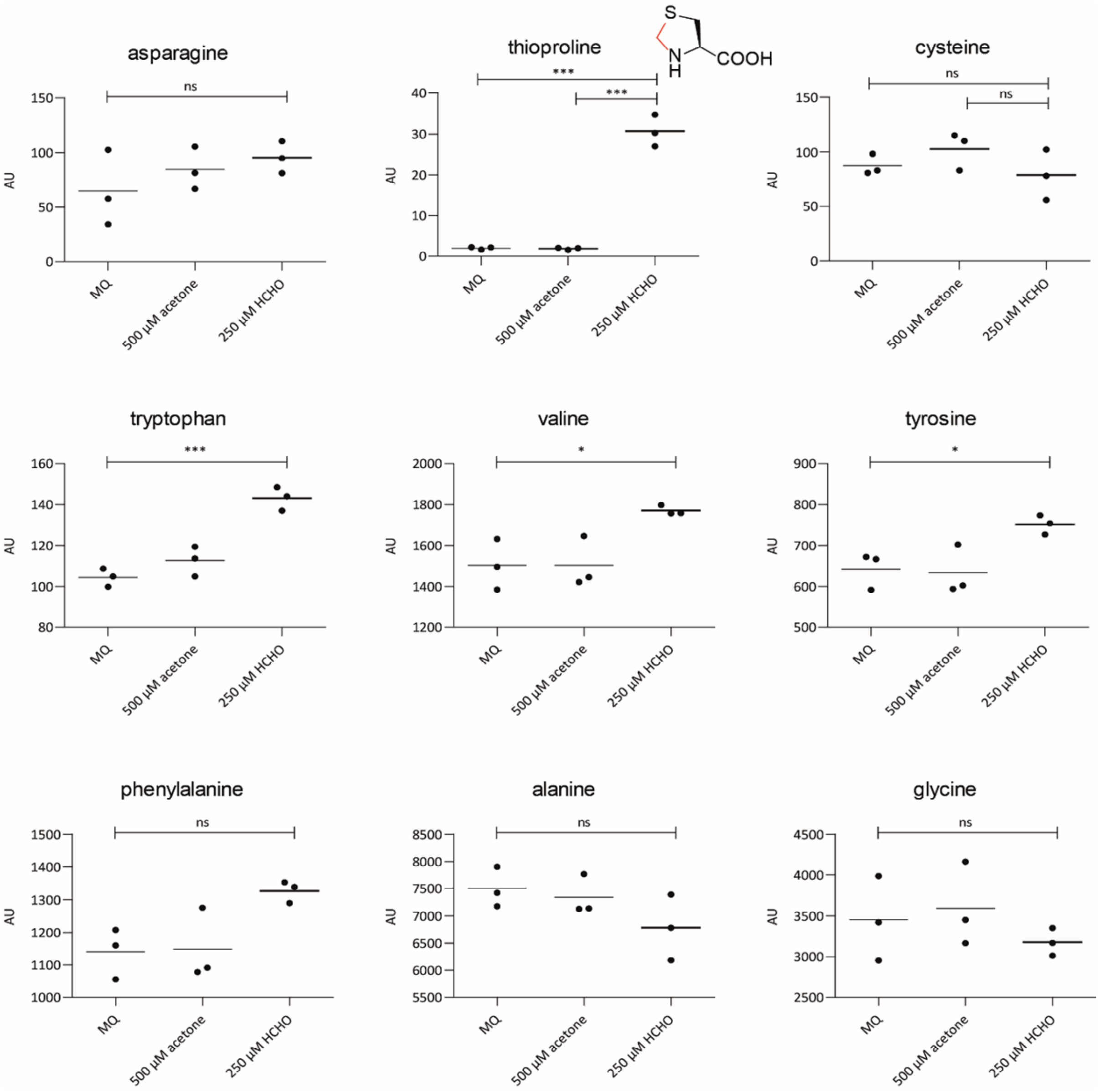


**Figure S6 | Metabolomics analyses of acetone or HCHO-treated HEK293T cells.** HEK293T cells (grown using non-dialysed FBS) were treated with two cumulative treatments of aldehydes that were 24 h apart. Cells were harvested 16 h after the second treatment. The second dose was 1.5-fold that of the preceding dose. Cumulative doses are shown in the graphs.

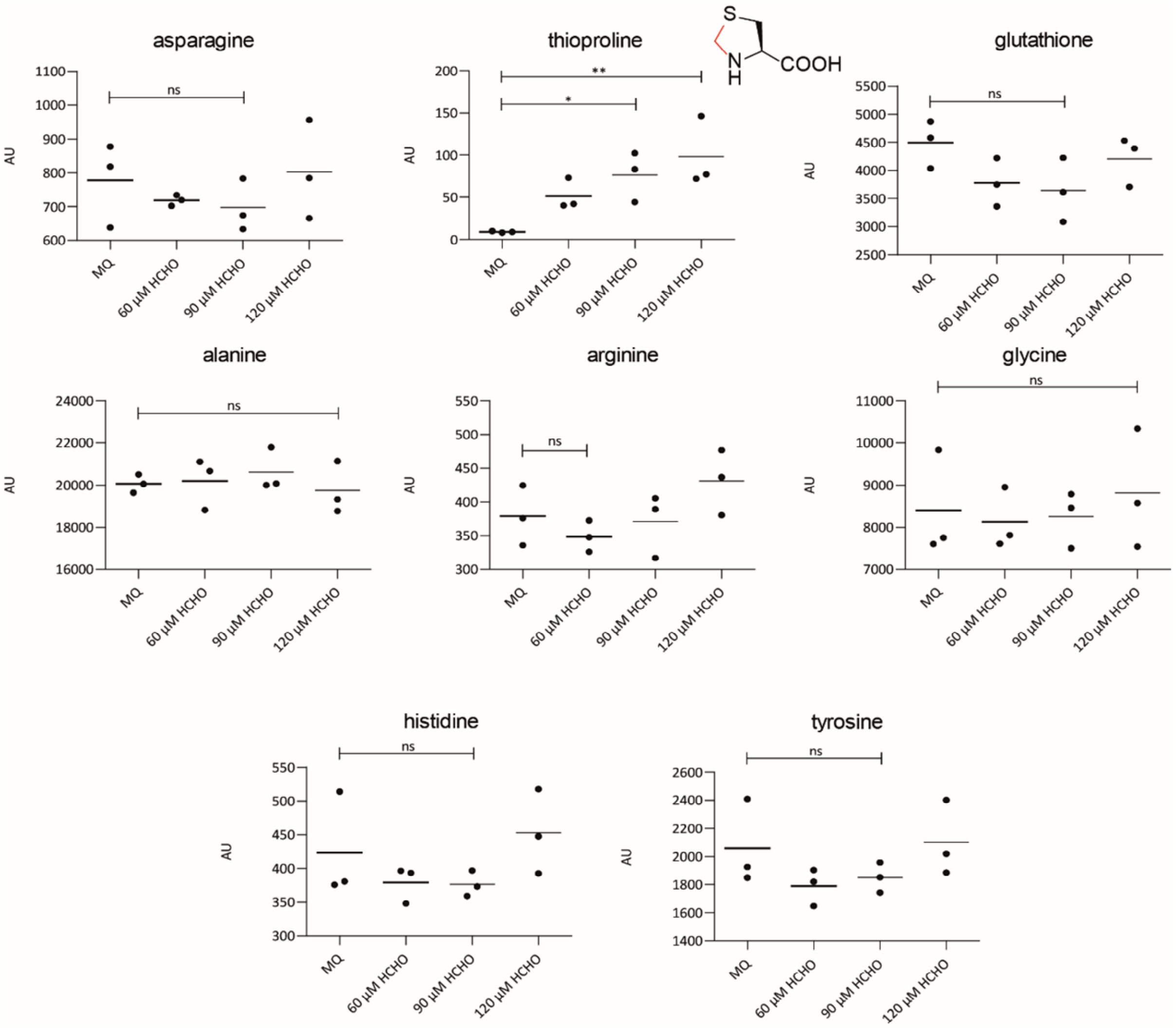


**Figure S7 | Metabolomics analyses of HCHO-treated HEK293T cells.** HEK293T cells (grown using non-dialysed FBS) with treatment with the indicated doses of HCHO for 16 h.

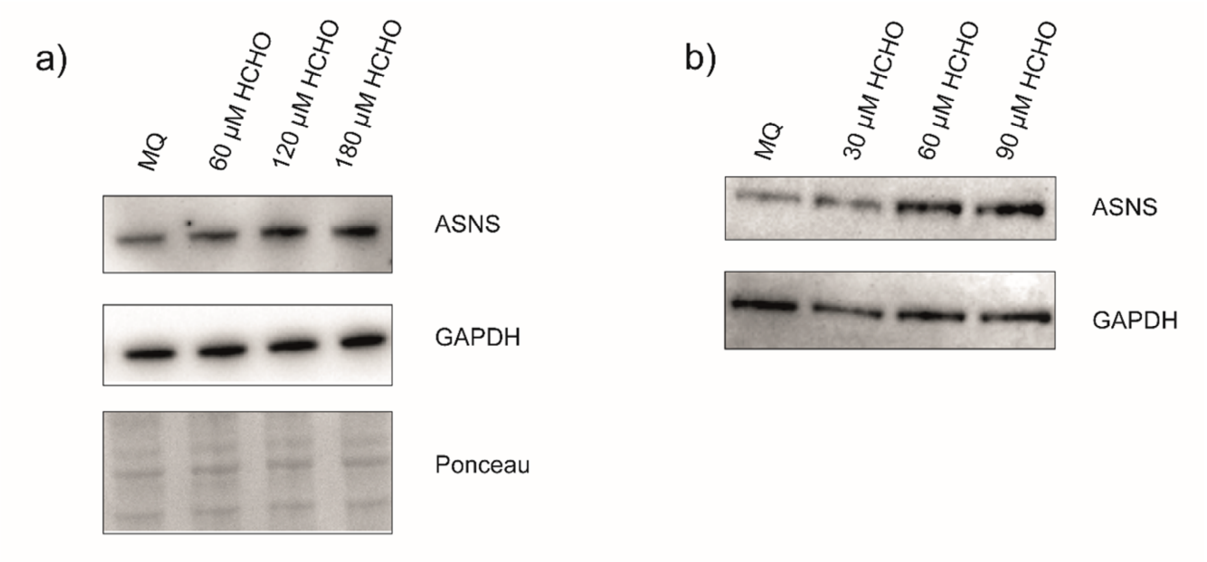


**Figure S8 | Evidence that HCHO may upregulate ASNS levels in cells. a,b.** Independent replicates of the experiment shown in **Figure 1d**. HEK293T cells were treated with the indicated amounts of HCHO or MQ water control for 9 h (a) or 14 h (b), followed by analysis of ASNS and GAPDH protein levels by western blotting. A dose-dependent increase in ASNS protein levels is observed.

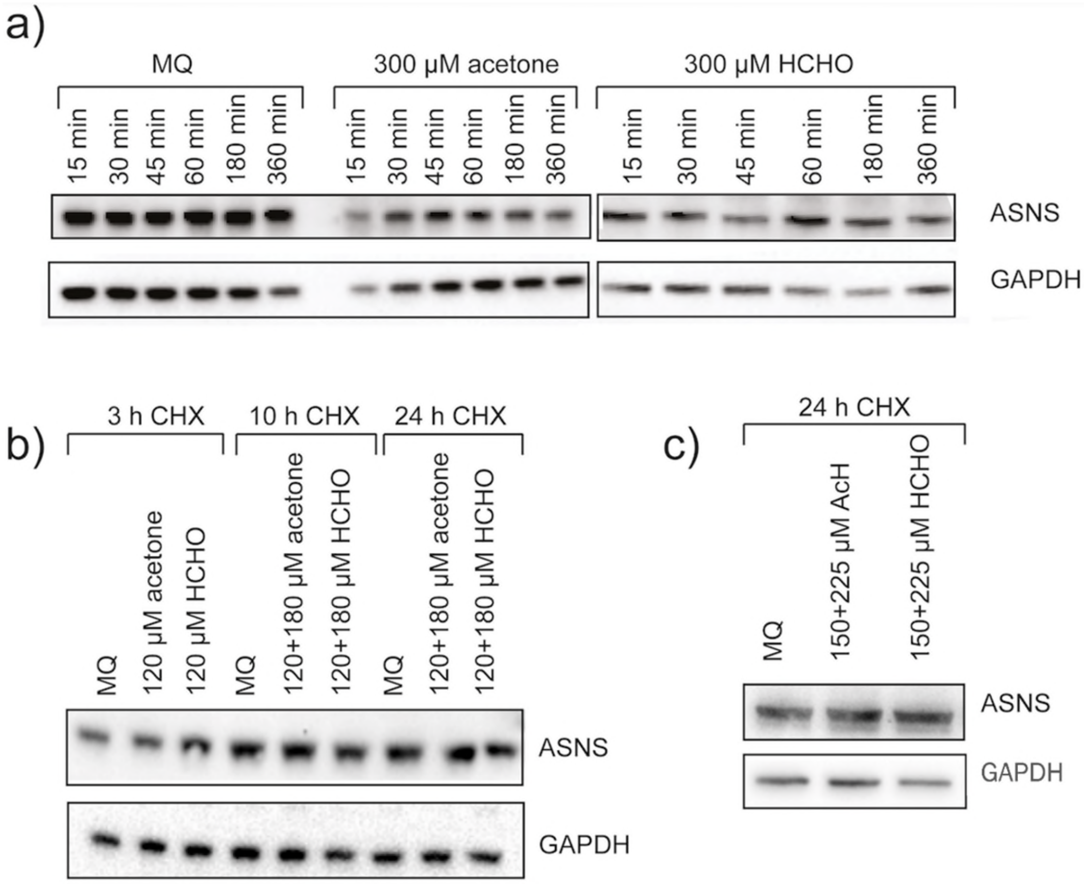


**Figure S9 | Studies on ASNS stability in cells treated with cycloheximide (CHX). b.** HEK293T cells were treated with the indicated amounts of HCHO, acetone, or MQ water control for 14 h. Cells were then treated with CHX and harvested at the indicated time-points. ASNS levels appear consistent across the time-points but are lower in the acetone- and HCHO-treated samples. **b.** HEK293T cells were treated with either HCHO (120 µM), acetone (120 µM), or MQ water control for 14 h. The carbonyl compounds were added from 100 mM stock solutions in MQ water. The cells were then treated with CHX (100 µM) and harvested after a further 3 h incubation. HEK293T cells were also treated with HCHO (120 µM), acetone (120 µM) or MQ water control for 14 h, followed by addition of CHX (100 µM). After 7.5 h post CHX treatment, further treatment of HCHO or acetone (180 µM) was conducted. The cells were then harvested 10 h and 24 h after CHX addition. No significant difference in ASNS stability is observed across the samples. **c.** HEK293T cells were treated with MQ water, AcH or HCHO overnight and subsequently treated with a second dose of MQ water, AcH or HCHO along with CHX, then harvested after 24 h. The final CHX concentration was 100 µM in all experiments (from a 100 mM DMSO stock). No significant difference in ASNS stability is observed across the samples.

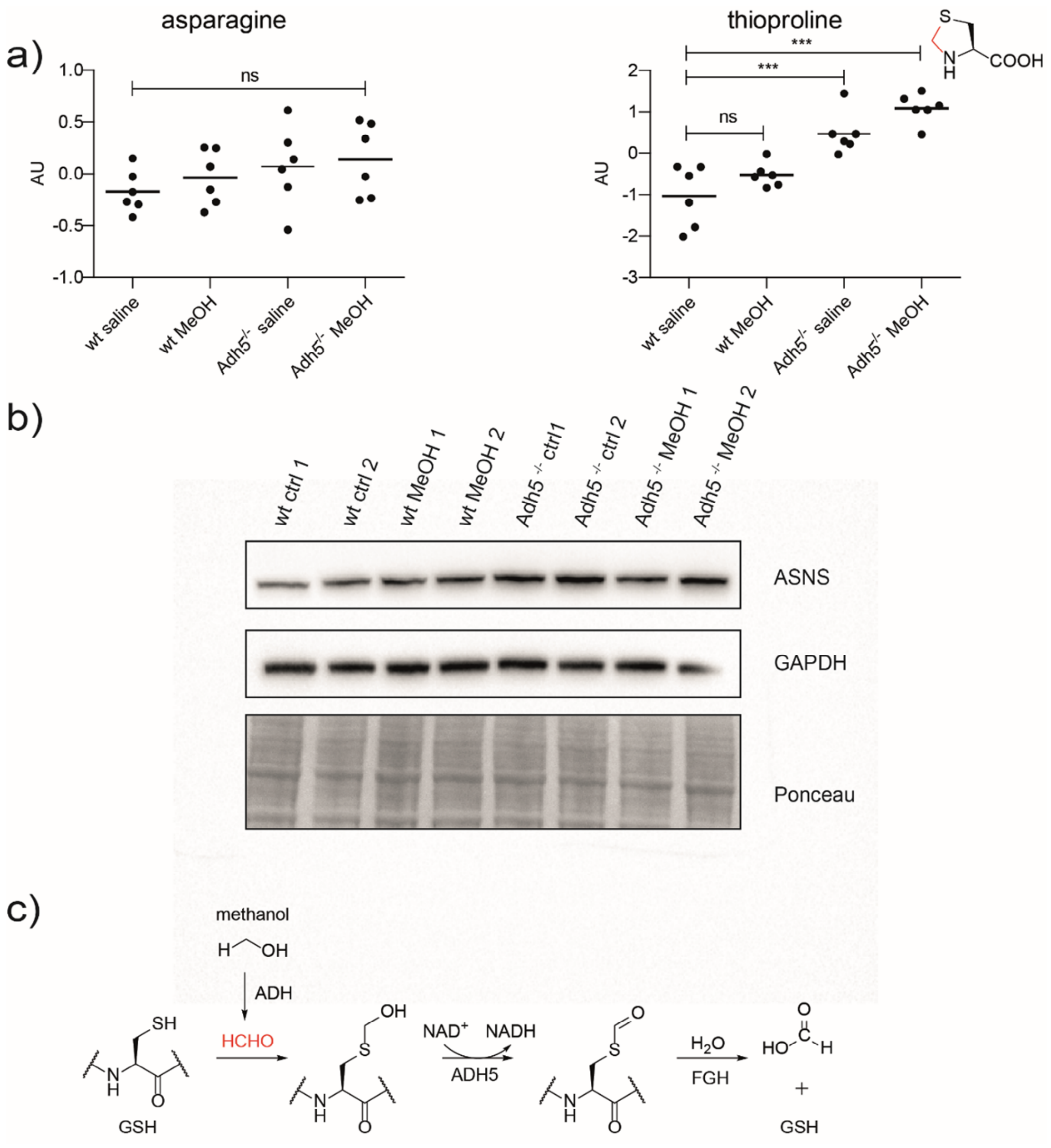


**Figure S10 | Metabolomics and western blot analyses of mouse livers.** Liver metabolite levels (**a**) and ASNS protein levels (**b**), in livers from wildtype (wt) or *Adh5*$^{-/-}$ mice treated with saline solution (control) or methanol (MeOH) for 6 h. An Origene ASNS antibody (TA503444) was used to obtain signals for the mouse samples (n=2). Individual data points and means are shown. **c.** Outline scheme showing the origin and catabolism pathway for HCHO.[69] FGH: *S*-formyl-glutathione hydrolase.

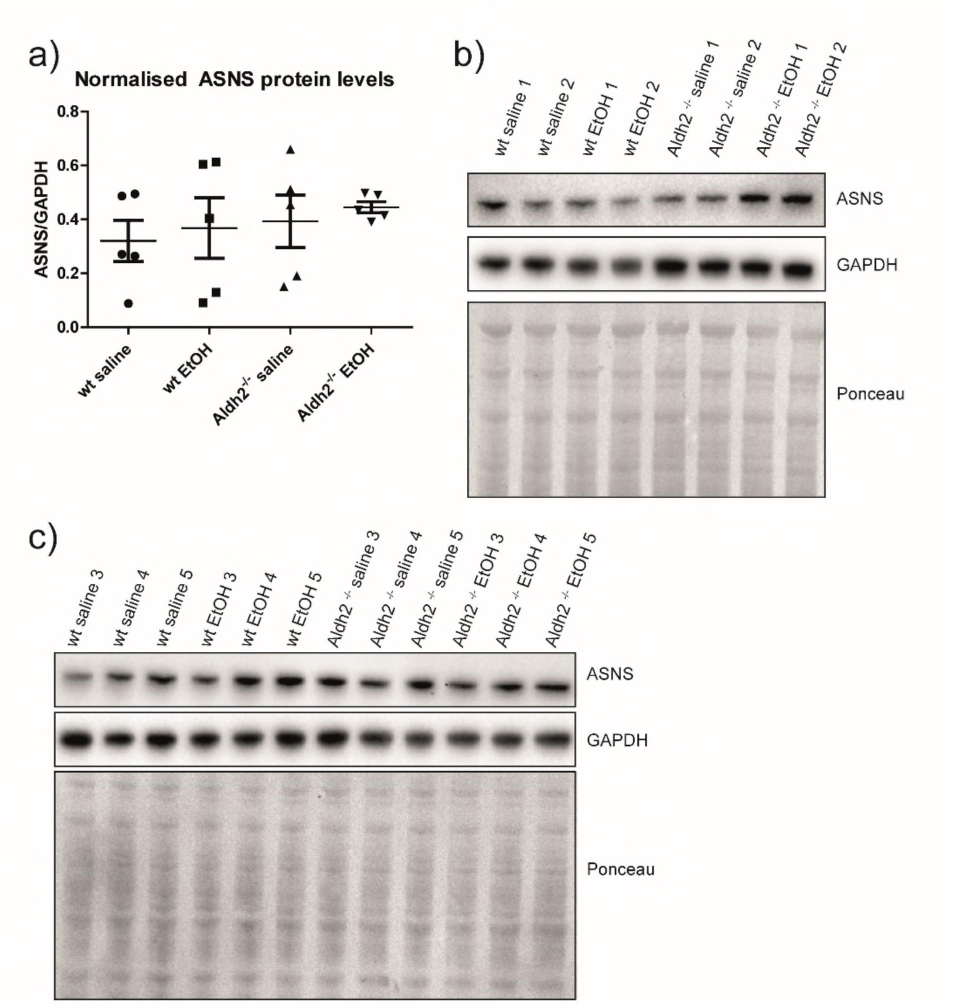


**Figure S11 | Effects of EtOH treatment and *Aldh2*$^{-/-}$ deficiency on ASNS protein levels in mice.** Wildtype (wt) or *Aldh2*$^{-/-}$ mice were treated with saline as a control or EtOH and their livers were analysed by western blots. **a.** Quantification and normalization of the signals obtained in (**b**) and (**c**).

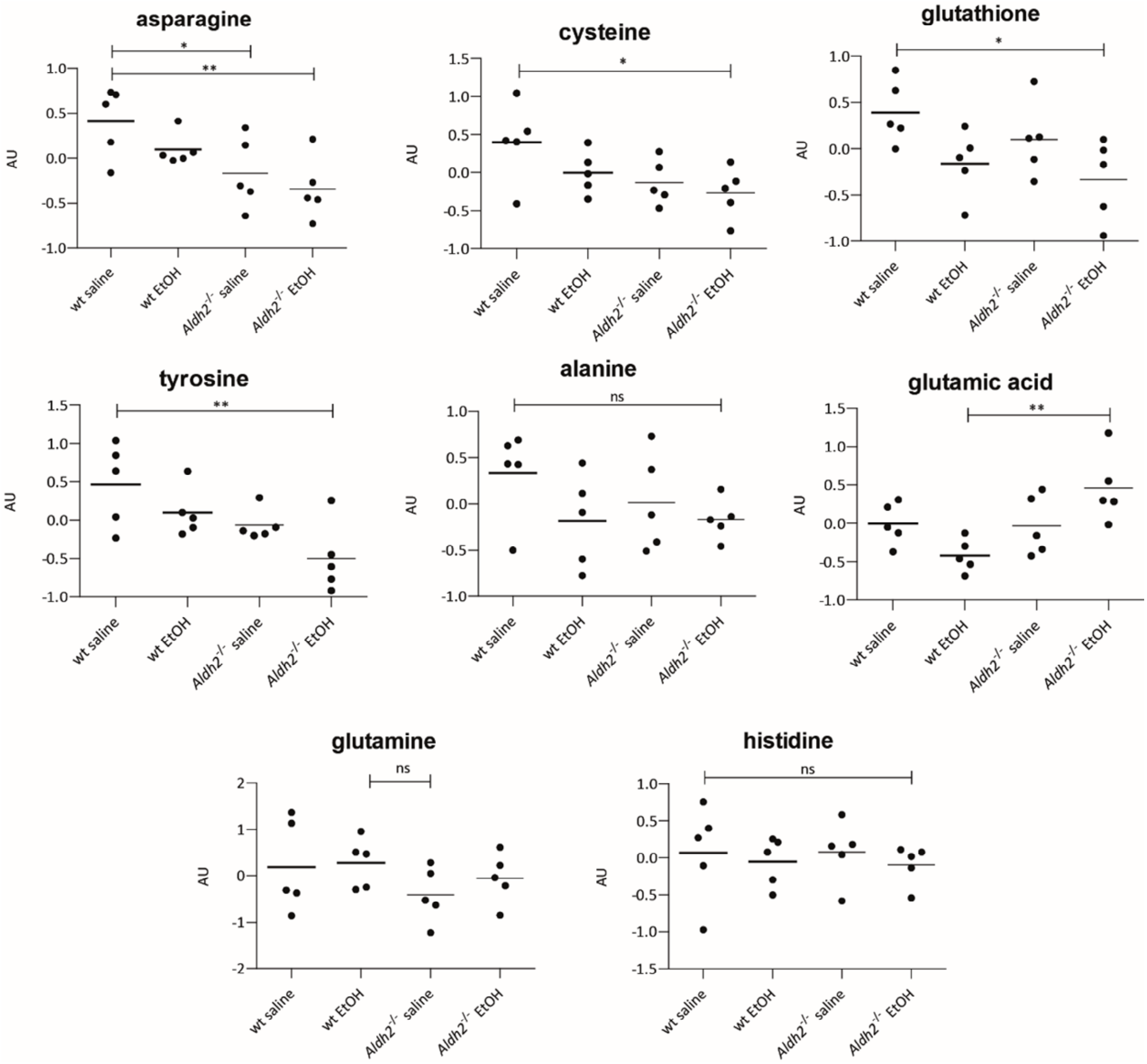


**Figure S12 | Metabolomics analyses of mouse livers.** Wildtype (wt) or *Aldh2*$^{-/-}$ mice were treated with saline as a control or EtOH. Notably, MTCA was not detected in any of these samples.

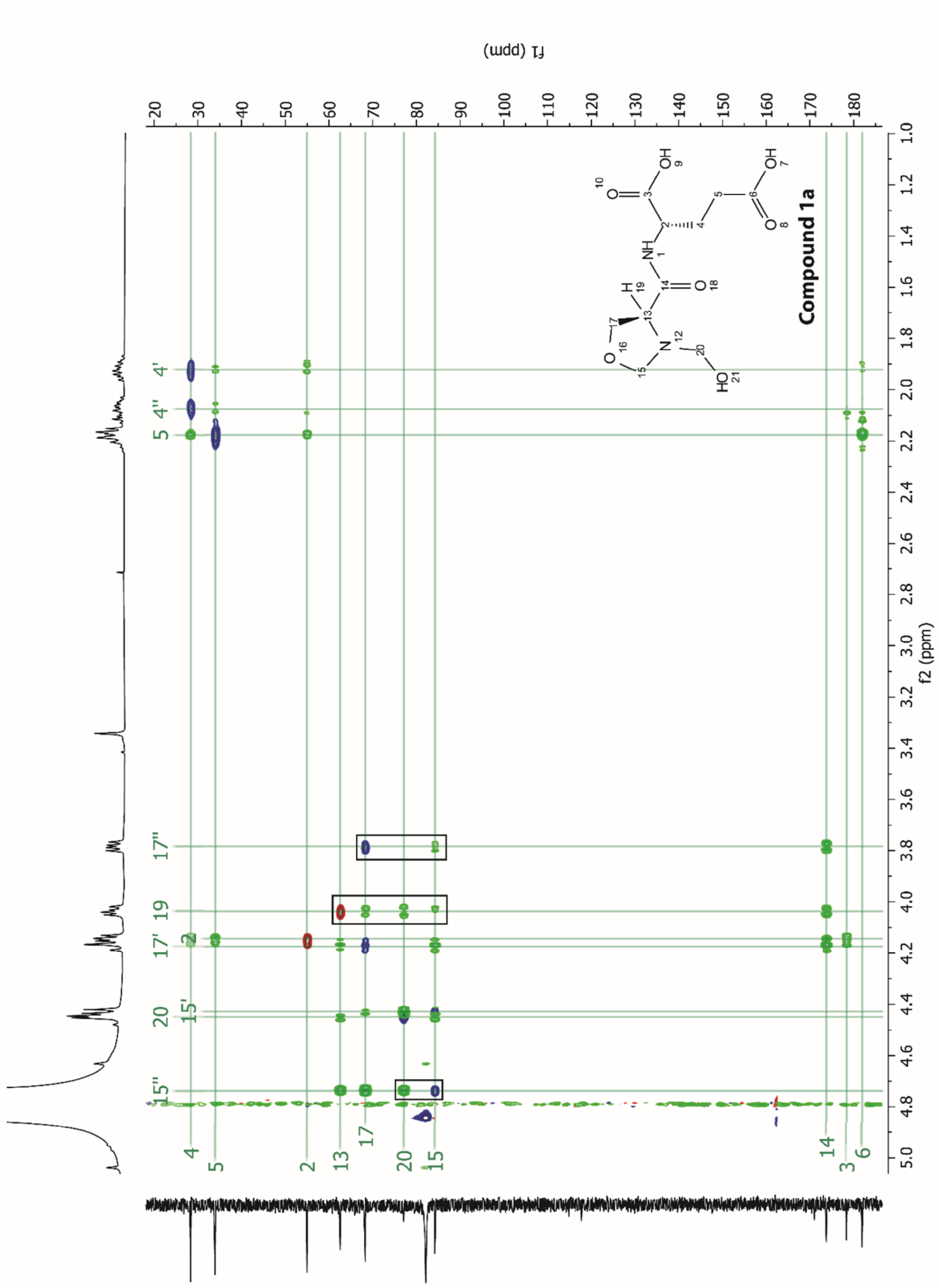


**Figure S13 | Overlay of 2D $^{1}H$-$^{13}C$ HSQC and 2D $^{1}H$-$^{13}C$ HMBC spectra for *in situ*-analysed SerGlu-derived 1a (400 MHz).** Important interactions are highlighted in a rectangle. The x-axis shows the $^{1}H$ spectrum and the y-axis shows the $^{13}C$ spectrum.

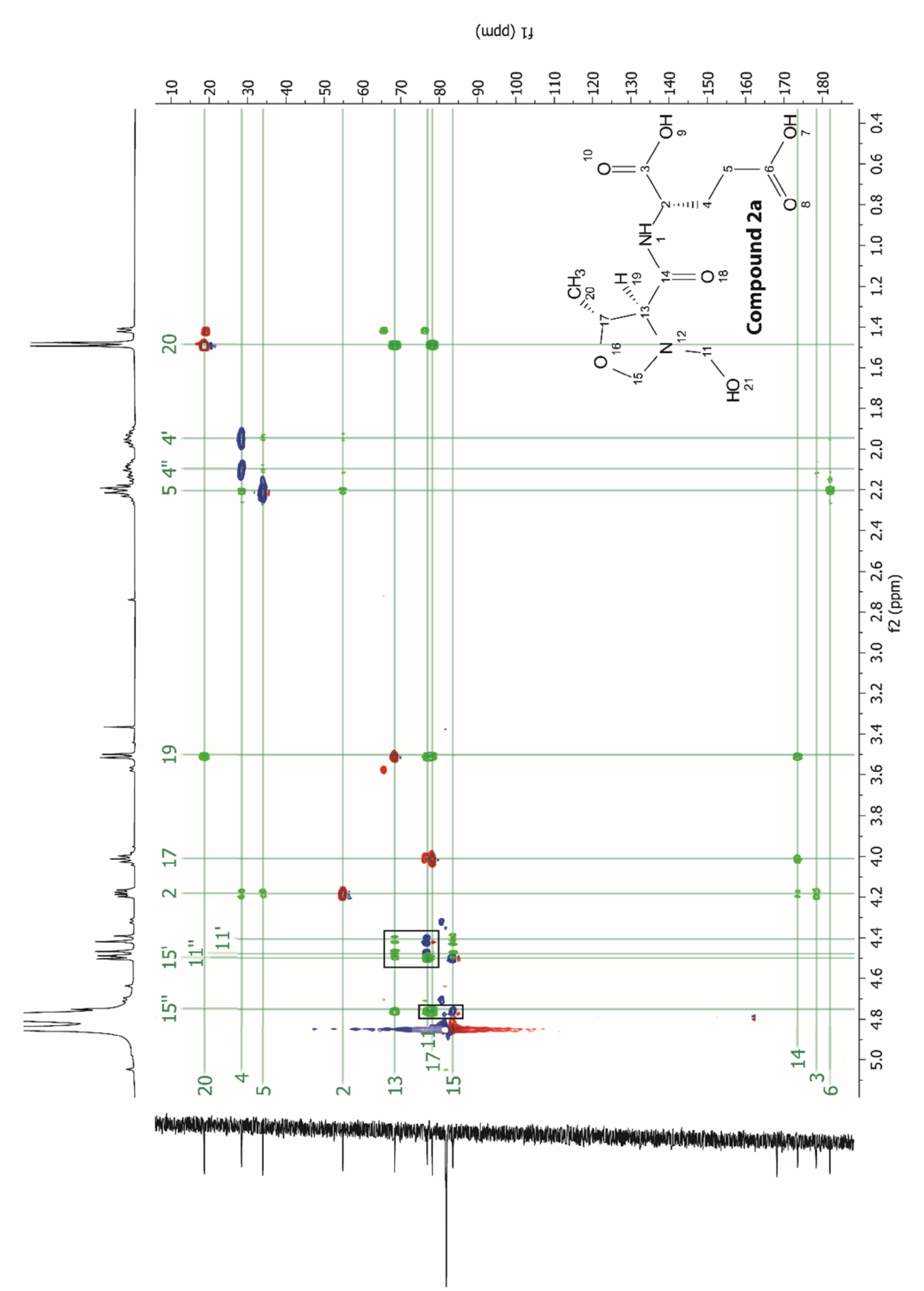


**Figure S14 | Overlay of 2D $^{1}H$-$^{13}C$ HSQC and 2D $^{1}H$-$^{13}C$ HMBC spectra for *in situ*-analysed ThrGlu-derived 2a (400 MHz).** Important interactions are highlighted in a rectangle.

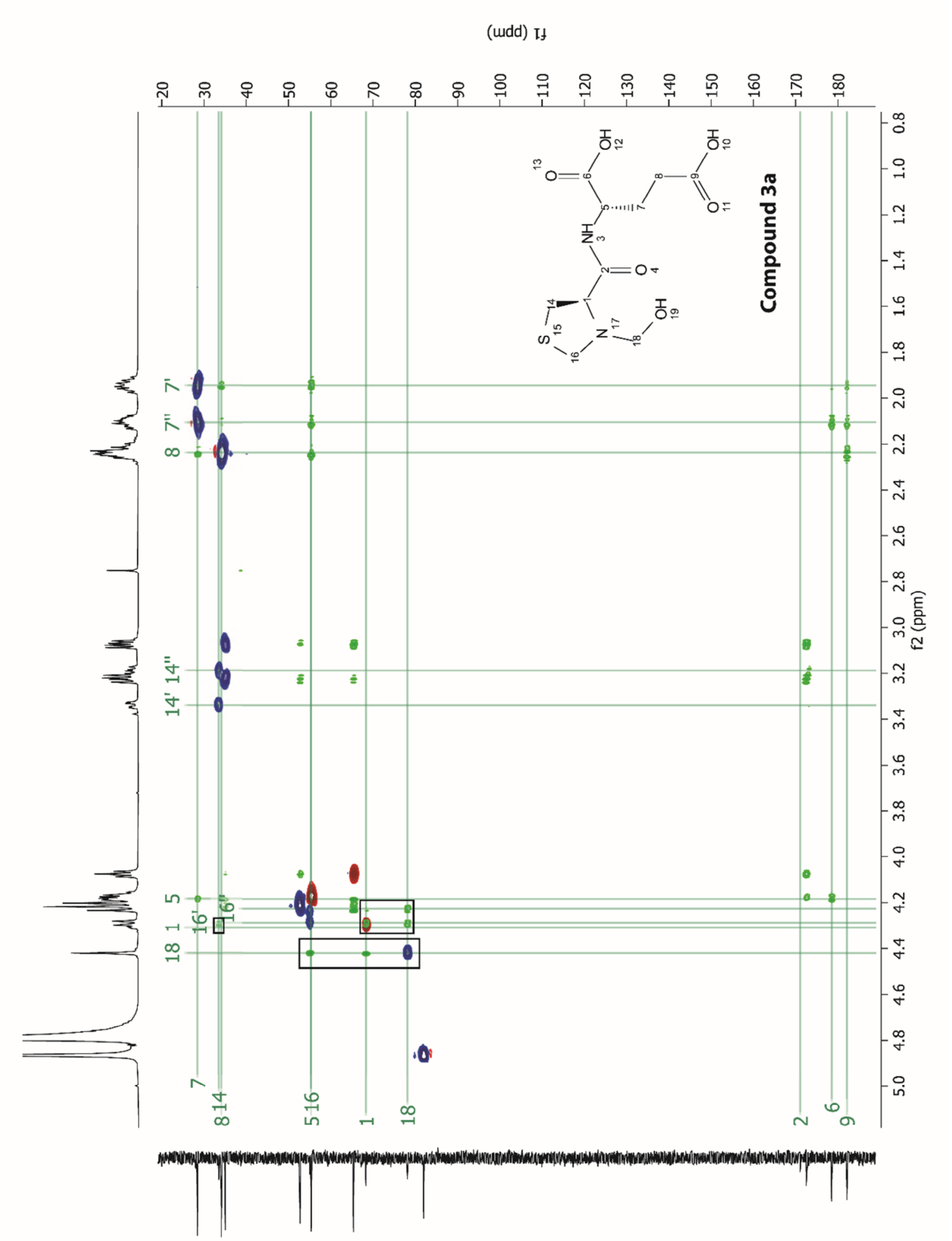


**Figure S15 | Overlay of 2D $^{1}$H-$^{13}$C HSQC and 2D $^{1}$H-$^{13}$C HMBC spectra for *in situ*-analysed CysGlu-derived 3a (600 MHz).** Important interactions are highlighted in a rectangle. The spectrum was acquired 3 days after the addition of HCHO to the dipeptide. Apart from **3a**, the mixture likely contains **3b**, that is the CysGlu-derived thiazolidine without a hemiaminal.

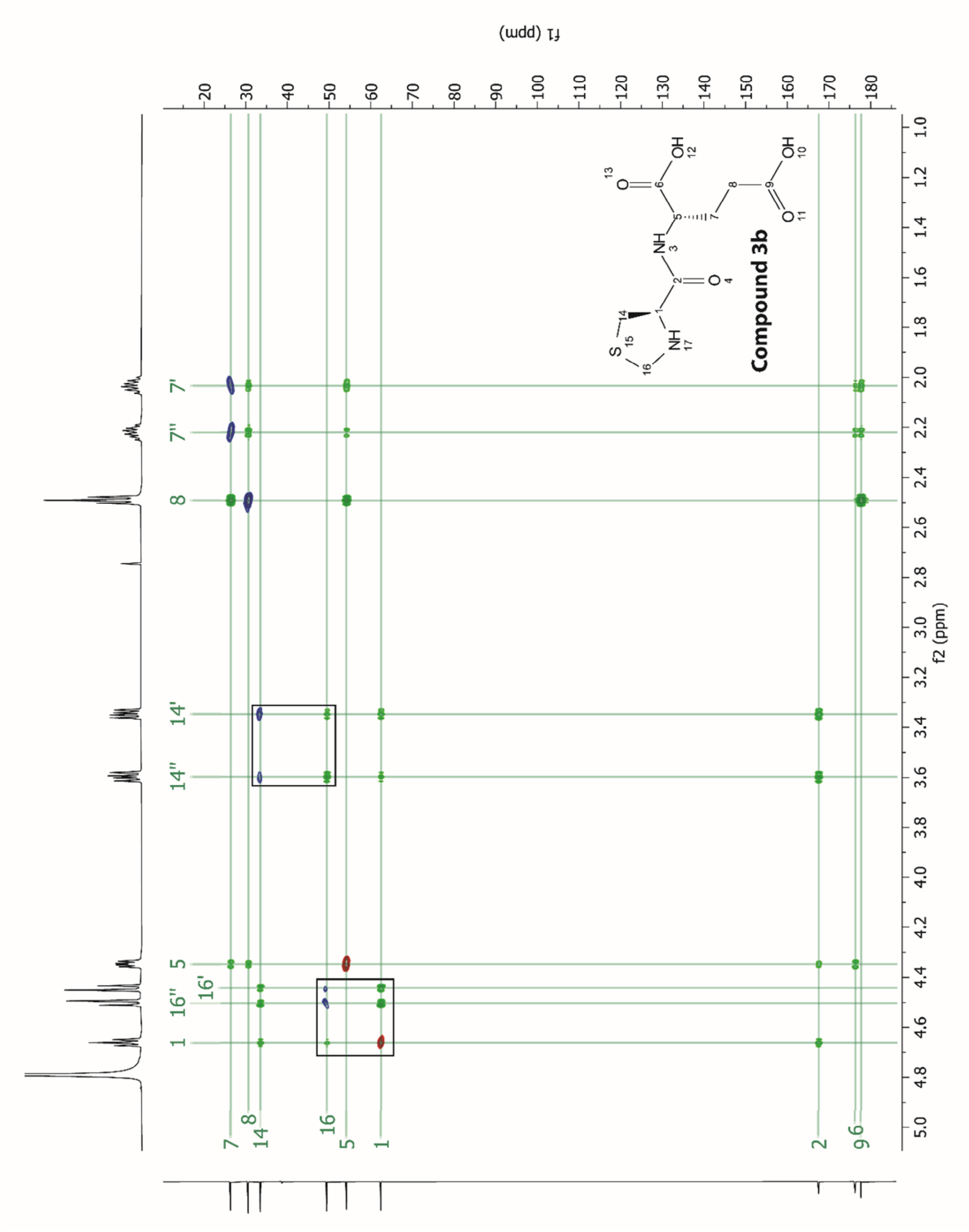


**Figure S16 | Overlay of 2D $^{1}H$-$^{13}C$ HSQC and 2D $^{1}H$-$^{13}C$ HMBC spectra for the HPLC-purified CysGlu-derived product 3b (600 MHz).** Important interactions are highlighted in a rectangle.

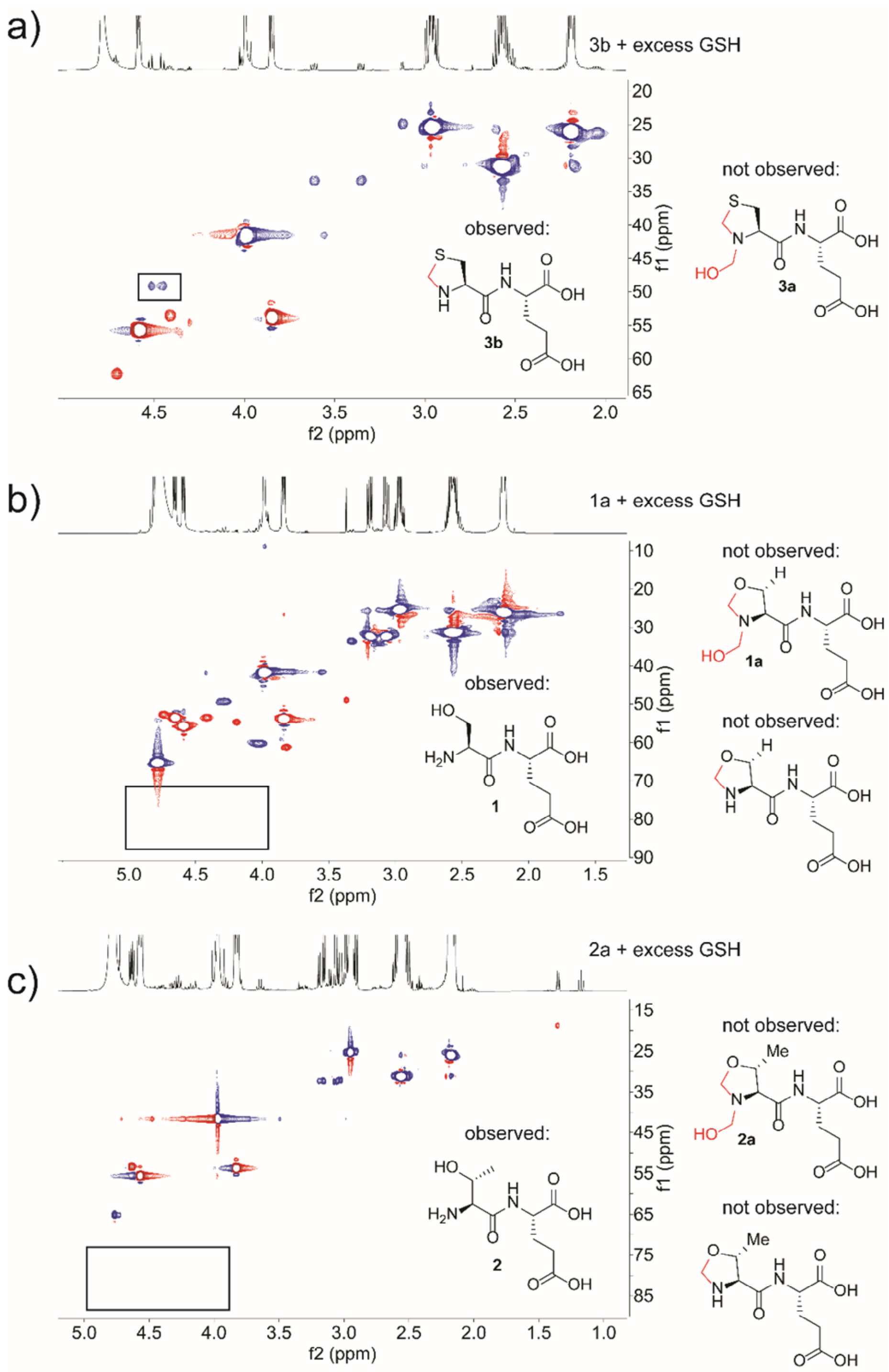


**Figure S17 | Thiazolidine 3b is relatively stable in the presence of excess GSH, whereas oxazolidines 1a and 2a are unstable. a.** NMR analysis of HPLC-purified **3b** with a 40-fold excess of GSH (600 MHz). The HCHO-derived thiazolidine methylene bridge signals are highlighted in a rectangle and are consistent with the HCHO-derived methylene signal of purified **3b** at $\delta_C$ 49.5 ppm. **B,c. 1a** (b, 600 MHz) and **2a** (c, 400 MHz) were generated *in situ*, followed by addition of a 40-fold excess of GSH (relative to dipeptide). The rectangle indicates the region where HCHO-derived oxazolidine methylene bridge signals should appear ($\delta_C$ 84.2 ppm for **1a** and $\delta_C$ 83.6 ppm for **2a**). The absence of signals indicates **1a** and **2a** are unstable in the presence of excess GSH, resulting in reformation of **1** and **2**.

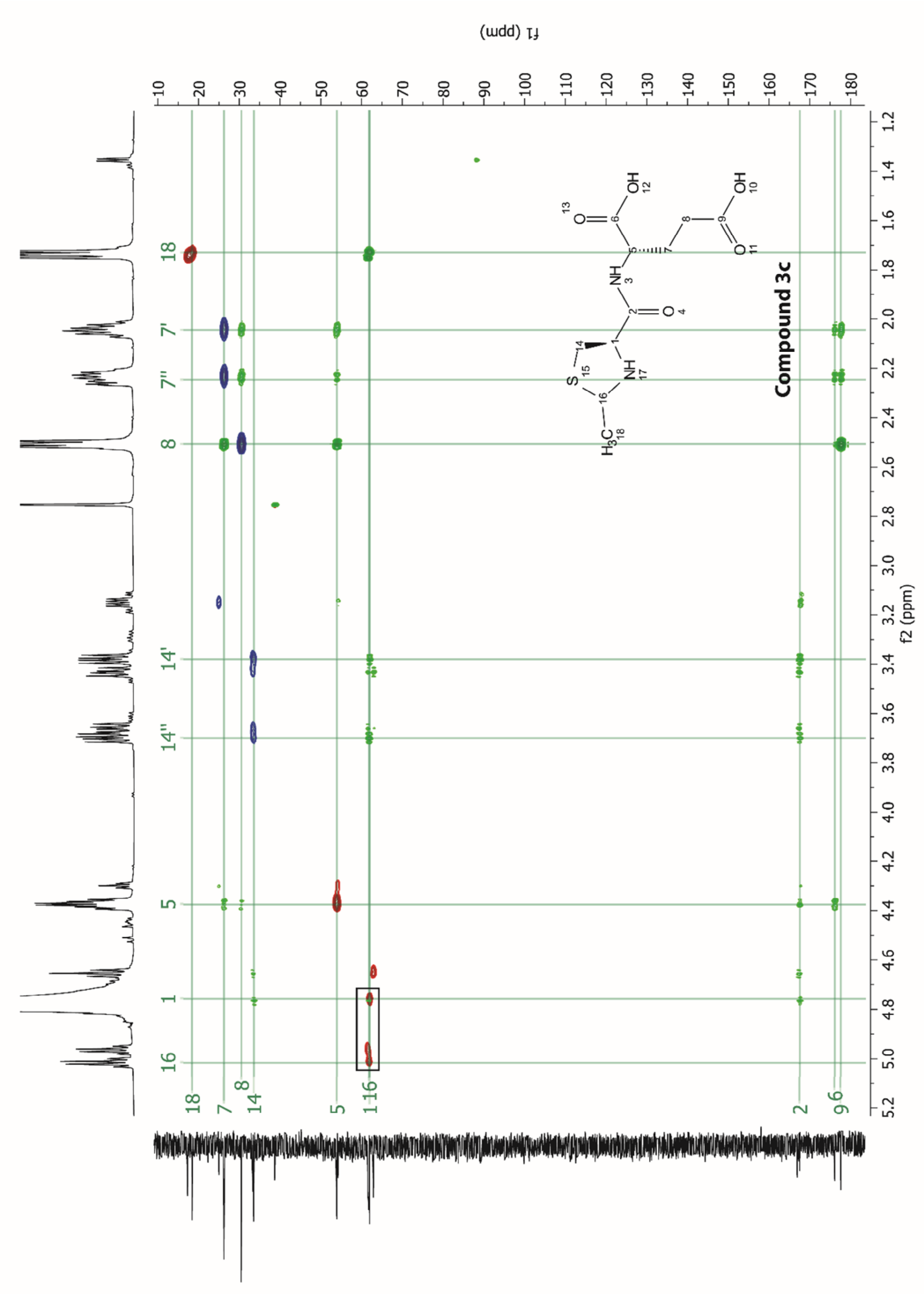


**Figure S18 | Overlay of 2D $^{1}H$-$^{13}C$ HSQC and 2D $^{1}H$-$^{13}C$ HMBC spectra for the HPLC-purified CysGlu-derived product 3c (600 MHz).** Important interactions are highlighted in a rectangle.

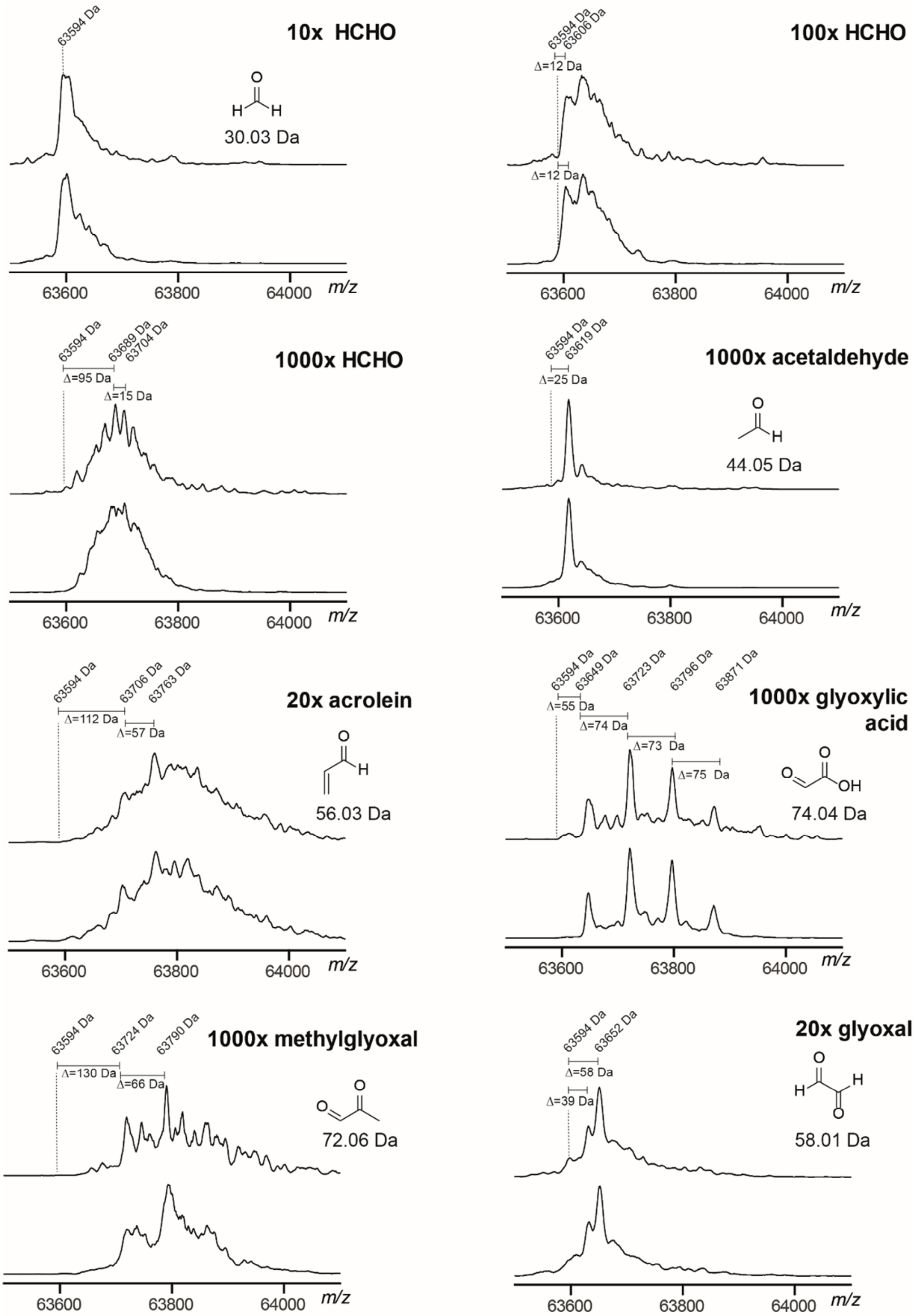


**Figure S19 | Protein-observed MS studies on the reaction of ASNS B with reactive carbonyl compounds**. ASNS B was pre-incubated with MQ water (ASNS B only) or the indicated excess of carbonyl compound (20 min, 37 °C). Reactions were subjected to solid phase extraction coupled to mass spectrometry (SPE-MS) analysis, in technical duplicates.

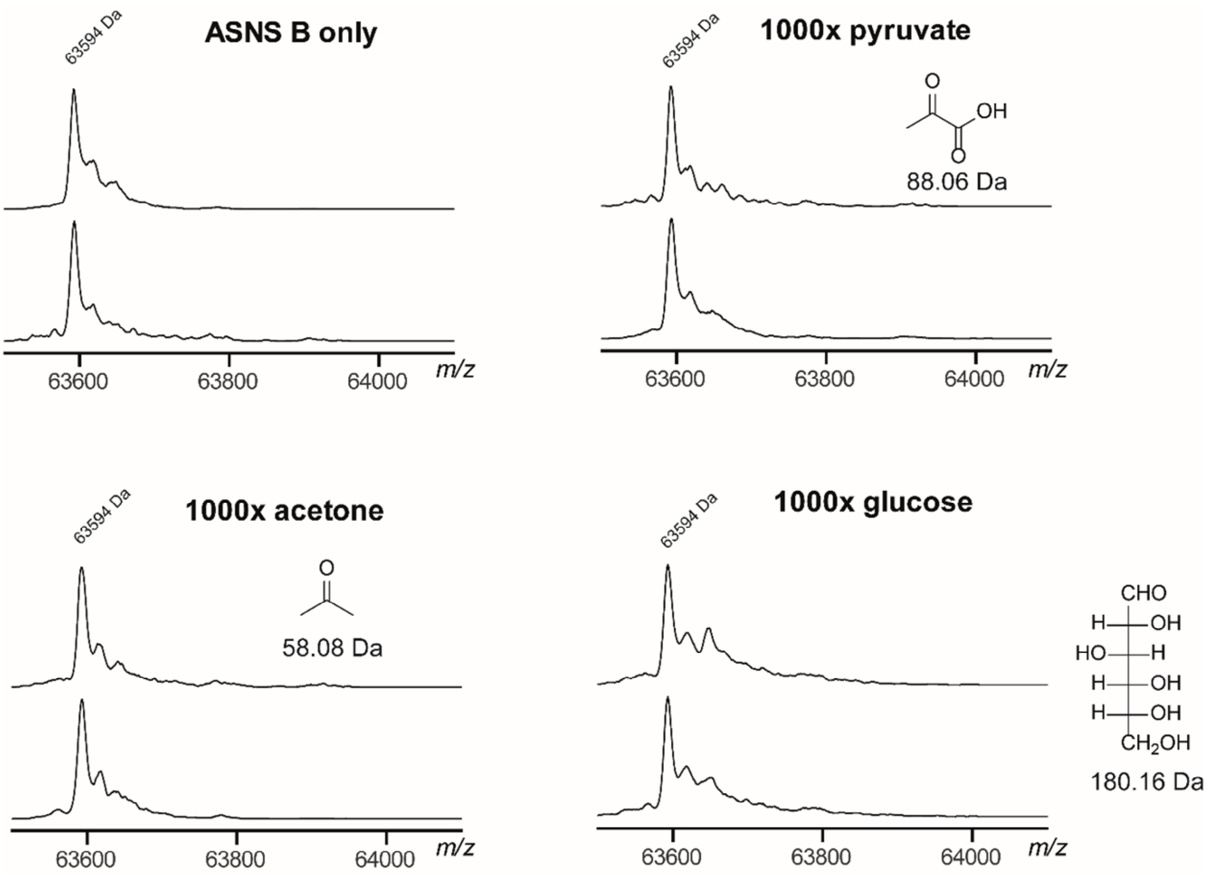


**Figure S20 | Protein-observed MS studies for potential reaction of ASNS B with relatively non-reactive carbonyl compounds**. ASNS B was pre-incubated with MQ water (ASNS B only) or the indicated molar excess of carbonyl compound for 20 min at 37 °C. The reactions were then subjected to analysis by SPE-MS, in technical duplicates.

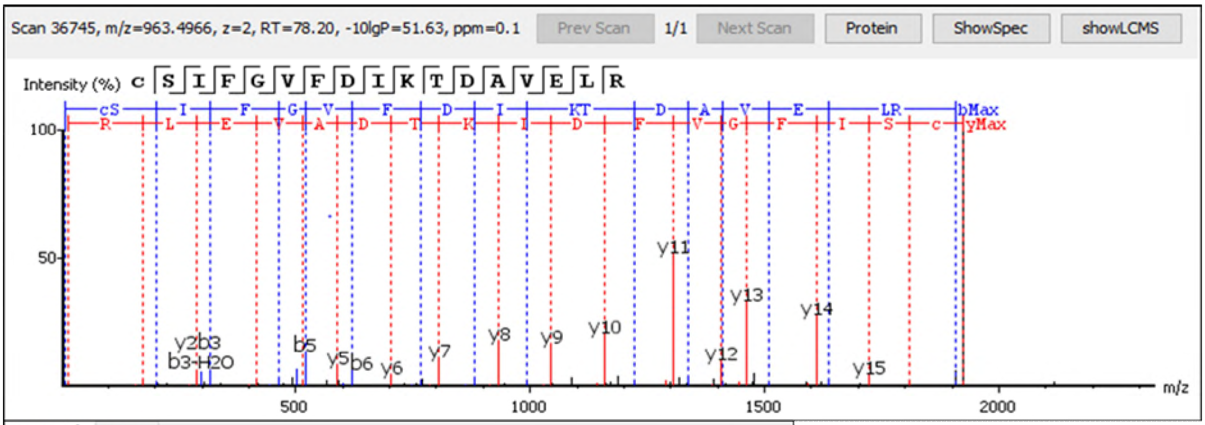


Ion Match | Survey

| # | b | b-H2O | b-NH3 | b (2+) | Seq | y | y-H2O | y-NH3 | y (2+) | # |
|---|---|---|---|---|---|---|---|---|---|---|
| 1 | 116.02 | 98.01 | 98.99 | 58.51 | C(+12.00) | | | | | 17 |
| 2 | 203.05 | 185.04 | 186.02 | 102.02 | S | 1809.97 | 1791.96 | 1792.95 | 905.49 | 16 |
| 3 | 316.13 | 298.12 | 299.11 | 158.57 | I | 1722.95 | 1704.93 | 1705.92 | 861.97 | 15 |
| 4 | 463.20 | 445.19 | 446.17 | 232.10 | F | 1609.86 | 1591.84 | 1592.84 | 805.43 | 14 |
| 5 | 520.22 | 502.21 | 503.20 | 260.61 | G | 1462.79 | 1444.78 | 1445.77 | 731.89 | 13 |
| 6 | 619.29 | 601.28 | 602.28 | 310.15 | V | 1405.77 | 1387.76 | 1388.75 | 703.38 | 12 |
| 7 | 766.36 | 748.34 | 749.33 | 383.68 | F | 1306.70 | 1288.68 | 1289.68 | 653.85 | 11 |
| 8 | 881.39 | 863.38 | 864.36 | 441.19 | D | 1159.63 | 1141.62 | 1142.61 | 580.32 | 10 |
| 9 | 994.47 | 976.46 | 977.44 | 497.74 | I | 1044.60 | 1026.60 | 1027.58 | 522.80 | 9 |
| 10 | 1122.57 | 1104.56 | 1105.54 | 561.78 | K | 931.52 | 913.51 | 914.50 | 466.26 | 8 |
| 11 | 1223.61 | 1205.60 | 1206.59 | 612.31 | T | 803.43 | 785.42 | 786.42 | 402.21 | 7 |
| 12 | 1338.64 | 1320.63 | 1321.61 | 669.82 | D | 702.38 | 684.37 | 685.35 | 351.69 | 6 |
| 13 | 1409.68 | 1391.67 | 1392.65 | 705.34 | A | 587.35 | 569.34 | 570.32 | 294.18 | 5 |
| 14 | 1508.74 | 1490.74 | 1491.72 | 754.87 | V | 516.31 | 498.30 | 499.29 | 258.66 | 4 |
| 15 | 1637.78 | 1619.78 | 1620.76 | 819.39 | E | 417.25 | 399.23 | 400.22 | 209.12 | 3 |
| 16 | 1750.87 | 1732.86 | 1733.85 | 875.94 | L | 288.20 | 270.19 | 271.18 | 144.60 | 2 |
| 17 | | | | | R | 175.12 | 157.11 | 158.09 | 88.06 | 1 |

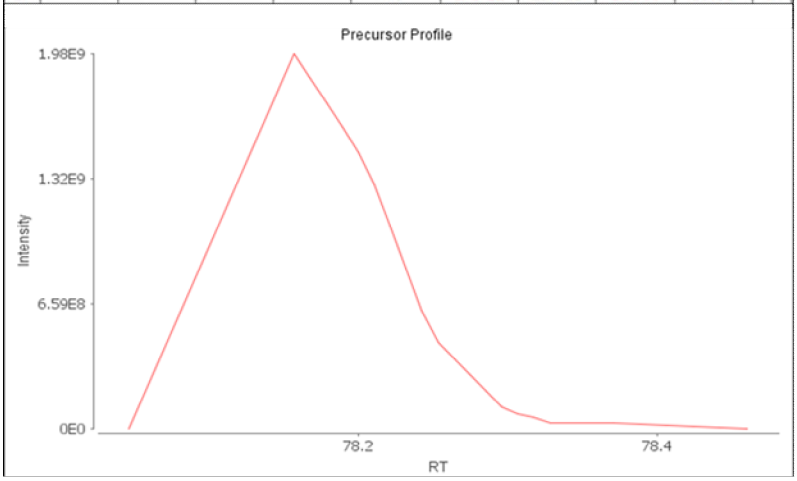


**Figure S21 | Evidence that recombinant ASNS B reacts with HCHO to form an *N*-terminal +12 Da adduct.** ASNS B was incubated with HCHO (200-fold excess, 37 °C, 16 h in 20 mM potassium phosphate, pH 7.4) before being subjected to trypsin digestion and LUMOS LC-MS/MS analyses. The b ions marked in blue correspond to an *N*-terminal +12 Da fragment.

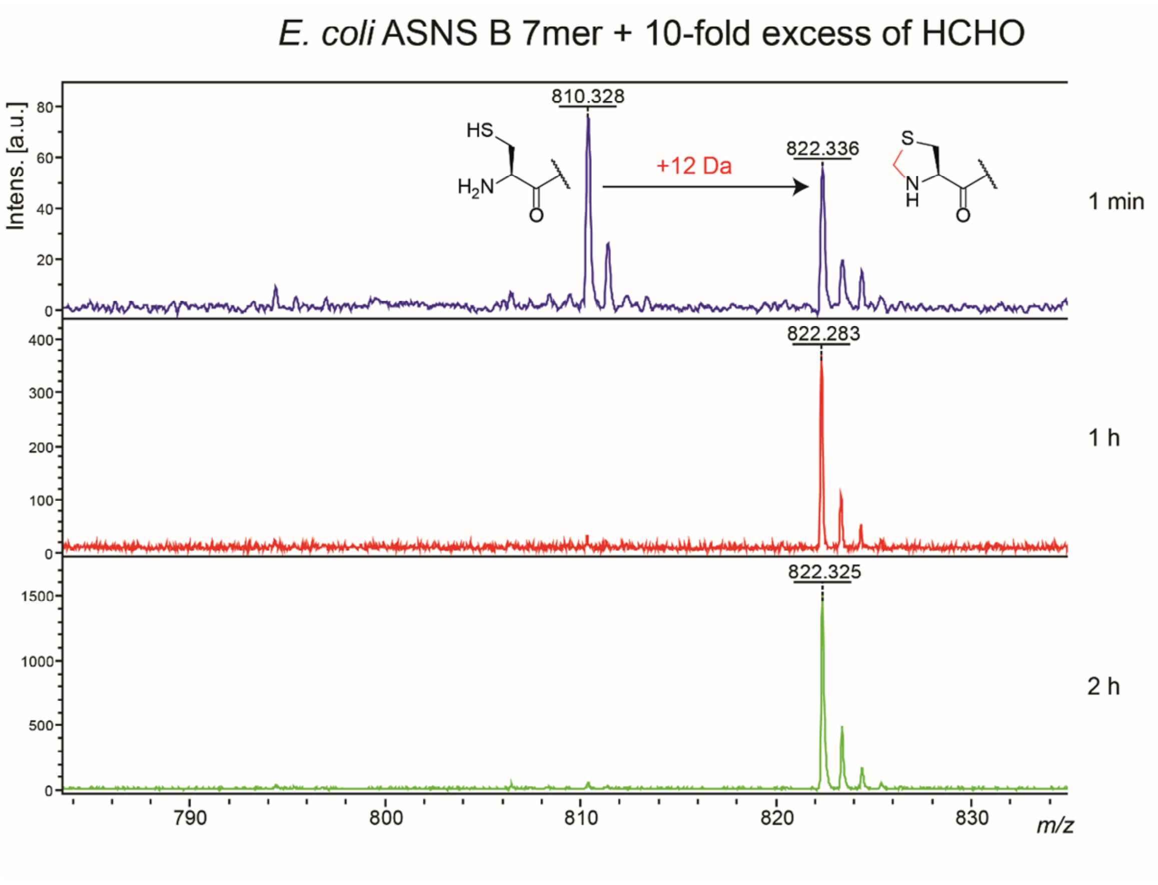


**Figure S22 | MALDI MS data obtained from addition of HCHO to the *N*-terminal ASNS B 7mer peptide.** The reaction mixture (2.5 µM peptide, 25 µM HCHO in 50 mM potassium phosphate at pH 7.4, ambient temperature) was analysed directly after addition of HCHO (1 min) and at the indicated time-points by MALDI MS. $K^+$ adducts of the substrate and product were observed. Peaks show parent peptide $[M+K]^+$: *m/z* 810; +12 Da HCHO adduct $[M+K]^+$: *m/z* 822. Peak areas were used to generate the graph in **Figure 22a**. The 7mer peptide ($NH_2$-CSIFGVF-$NH_2$) corresponds to the first 7 residues of *E. coli* ASNS B.

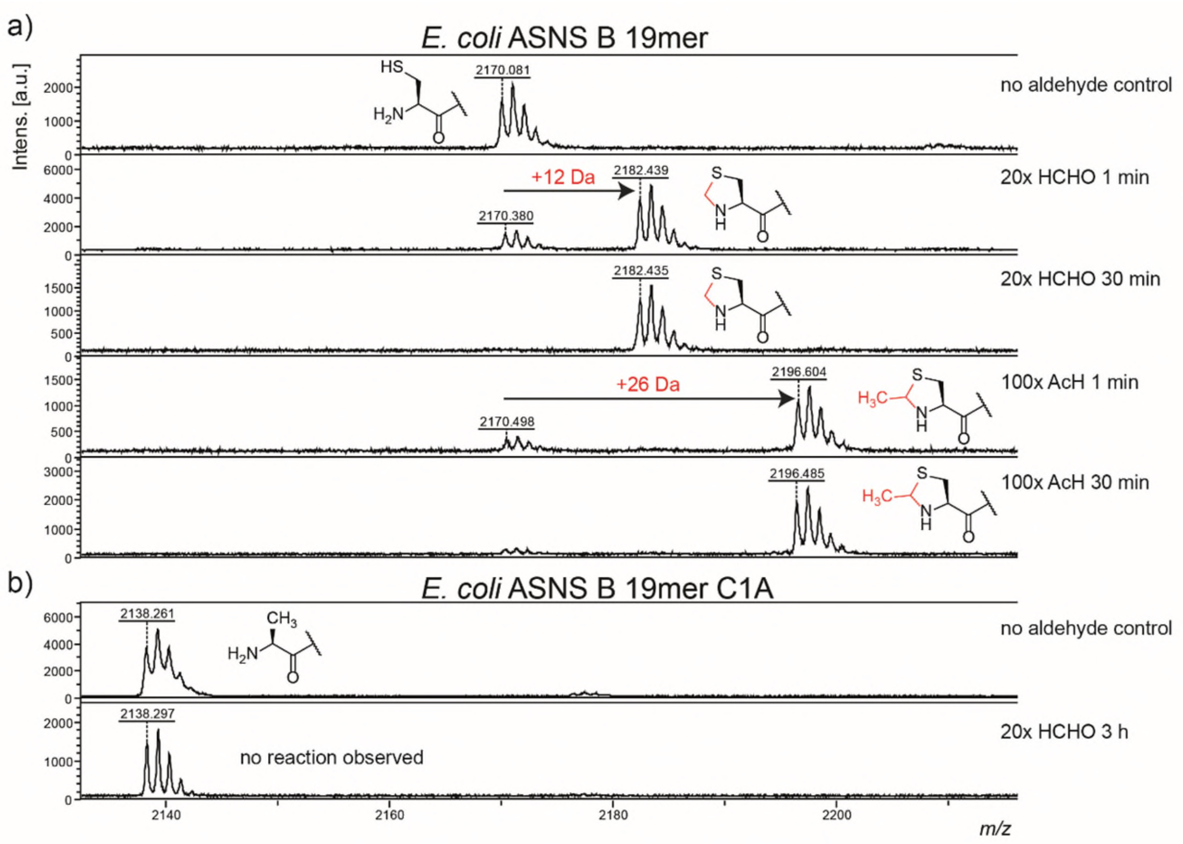


**Figure S23 | Reactions of aldehydes with an *N*-terminal ASNS B 19mer peptide.** The reaction mixture (25 µM peptide concentration, 500 µM HCHO or 2.5 mM $CH_3CHO$, 20 mM potassium phosphate at pH 7.4, ambient temperature) was analysed directly after HCHO addition (1 min) and after 30 min by MALDI MS (n=3 technical repeats, one representative result is shown). **a)** Peaks show parent peptide $[M+H]^+$: *m/z* 2170; +12 Da HCHO adduct $[M+H]^+$: *m/z* 2182; +26 Da $CH_3CHO$ (AcH) adduct $[M+H]^+$: *m/z* 2196. The 19mer peptide ($NH_2$-CSIFGVFDIKTDAVELRKK-COOH, Genscript) corresponds to the first 19 residues of *E. coli* ASNS B. **b)** Peaks show parent peptide $[M+H]^+$: *m/z* 2138. The 19mer peptide ($NH_2$-ASIFGVFDIKTDAVELRKK-COOH, Genscript) corresponds to the first 19 residues of *E. coli* ASNS B with a C1A substitution, which ablated observed reaction with HCHO.

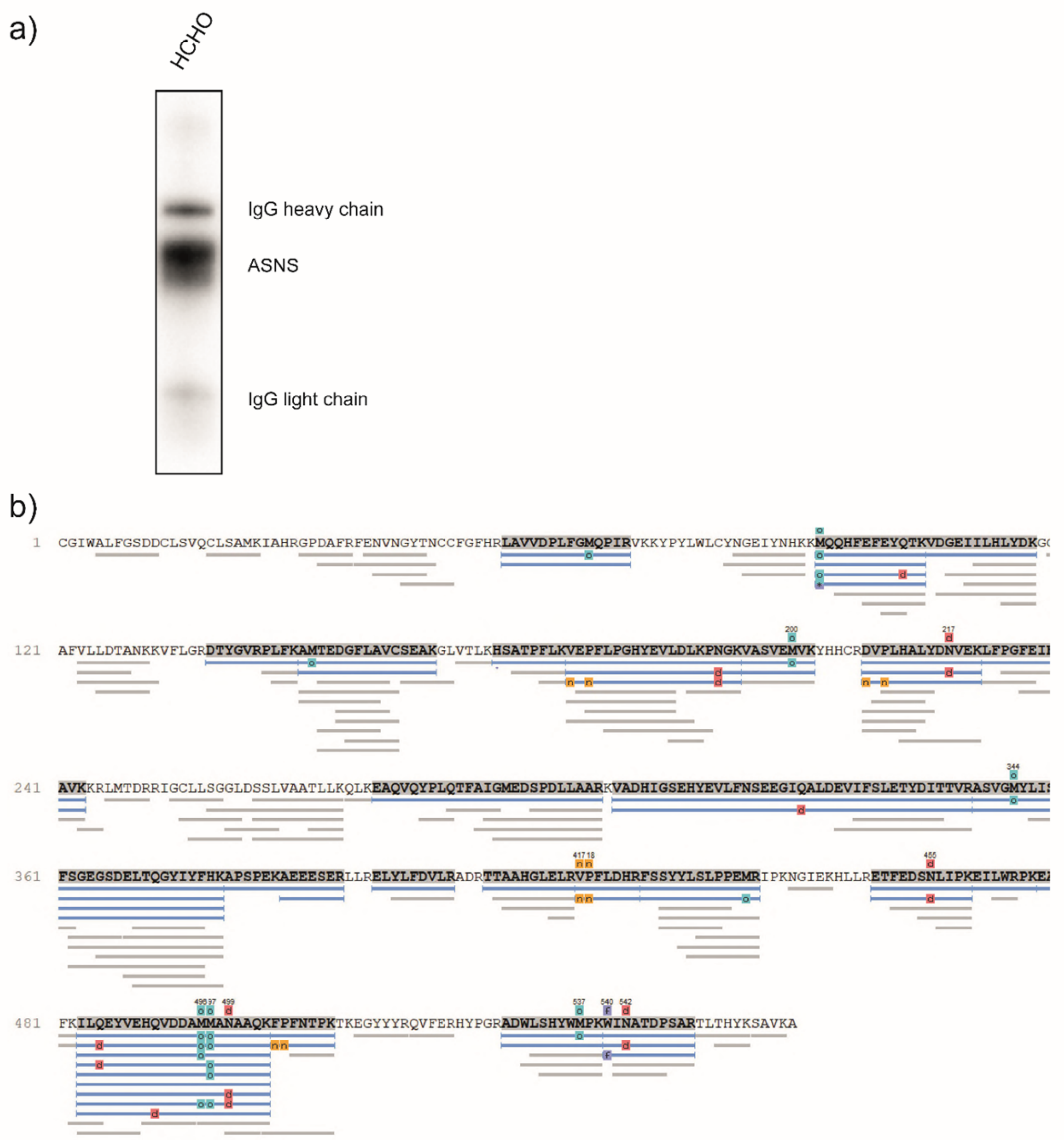


**Figure S24 | Trypsin digestion of immunoprecipitated ASNS. a.** HEK293T cells were treated with HCHO (100 µM final concentration) for 12 h, followed by a second treatment with HCHO (300 µM final concentration) for 3.5 h. After cell lysis, ASNS was immunoprecipitated (Abcam, ab226413), then digested with trypsin. **b.** LC-MS/MS results show no coverage of the immunoprecipitated *N*-terminal ASNS fragment upon trypsin digestion.

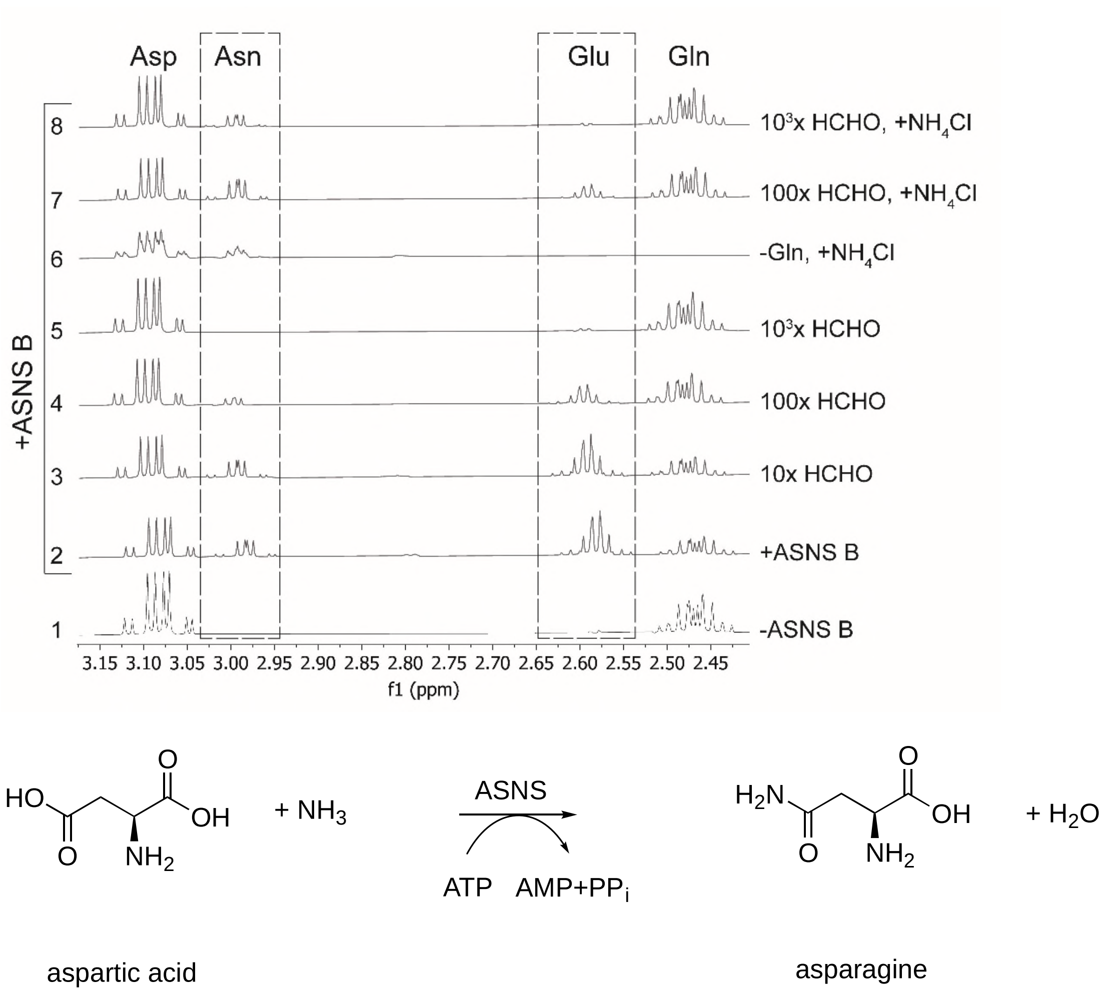


**Figure S25 | HCHO selectively inhibits the ASNS B *N*-terminal glutaminase reaction - independent replicate.** (a) $^1$H NMR (700 MHz) showing an independent replicate of the analysis in **Figure 3b**. The conversion of glutamine (Gln, $\delta_H$ 2.42-2.51) to glutamate (Glu, $\delta_H$ 2.55-2.61 ppm) and aspartate (Asp, $\delta_H$ 3.03-3.13 ppm) to asparagine (Asn, $\delta_H$ 2.97-3.00 ppm) in the presence of ASNS B (panel 2, +ASNS) is shown. The glutaminase reaction, which is dependent on the *N*-terminal cysteine of ASNS B, is inhibited by HCHO (panels 3-5). By contrast, the ASNS B catalysed reaction of Asp and ATP to give asparagine (shown below) functions in presence of $NH_4Cl$ and HCHO (panels 7+8).

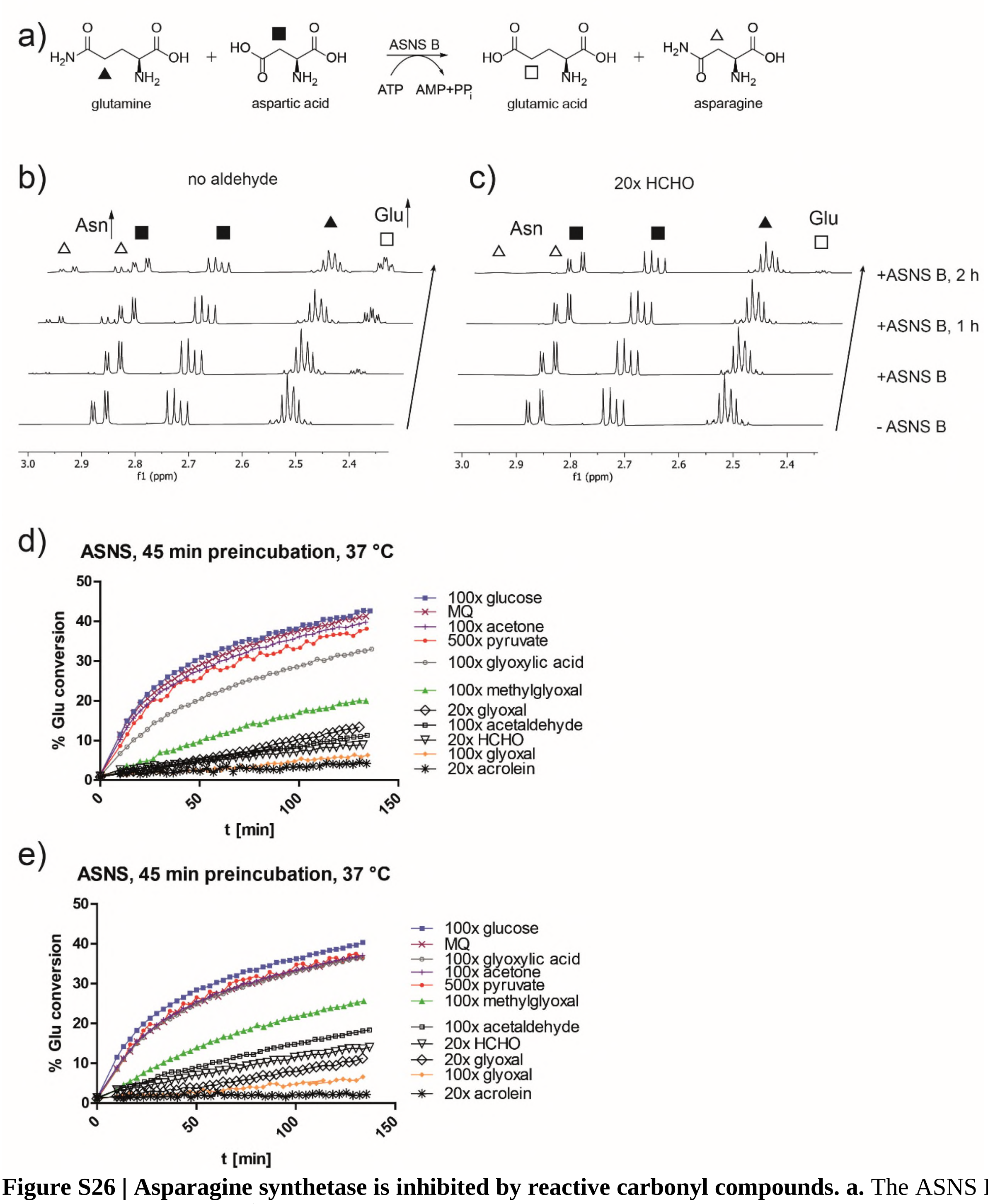


**Figure S26 | Asparagine synthetase is inhibited by reactive carbonyl compounds. a.** The ASNS B reaction. **b,c.** Exemplary $^{1}H$ NMR (700 MHz) datasets (water control, (**b**); 20-fold excess HCHO, (**c**)) showing ASNS B-catalysed conversion of glutamine (▲) and aspartic acid (■) to glutamic acid (□) and asparagine (△). The lowest spectrum shows substrates without ASNS B (-ASNS). The second acquisition was started 10 min after mixing of the assay components with ASNS B (+ASNS). Note the chemical shifts are slightly different to those in **Figure 3b**, which is likely a consequence of the $CF_3CO_2H$ used to quench ASNS B for the endpoint assay in **Figure 3b**. **d,e.** ASNS B was pre-incubated with the indicated molar excess of carbonyl compounds (or MQ water control) for 45 minutes at 37 °C; the solution was added to the assay components and the solutions were monitored by $^{1}H$ NMR (700 MHz). Independent duplicates are shown.

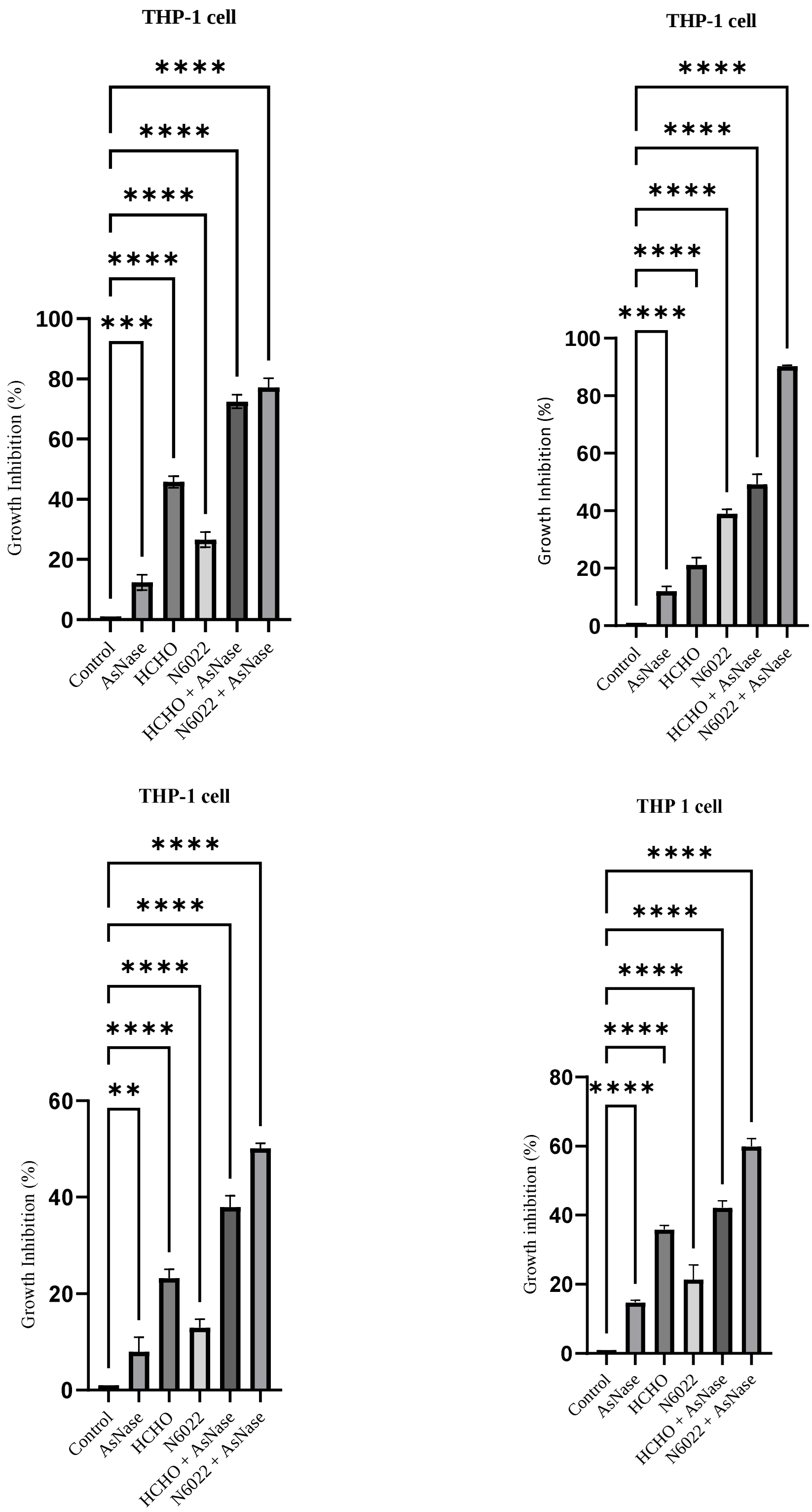


**Figure S27 | Inhibition of THP-1 cell growth by HCHO and/or asparagine**. Biological replicates are shown in **Figure 3e**. The extent of inhibition of THP-1 acute monocytic leukaemia cell growth by treatment with HCHO (80 µM), the HCHO metabolism inhibitor N6022 (10 µM), and/or asparaginase (0.05 U/ml) are shown. Growth inhibition (%) = 100-(treatment group OD/ non -treatment OD) x 100; OD = optical density. (** p value = 0.001 to 0.01, *** p value = 0.0001 to 0.001, ****p value <0.0001, n = 3 technical repeats).

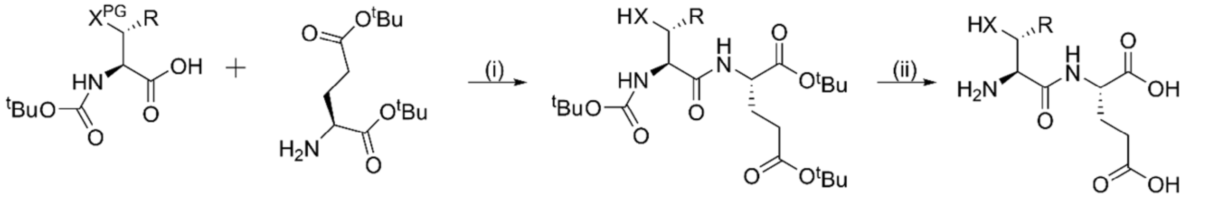


**1 (SerGlu):** R=H; X=O
**2 (ThrGlu):** R=Me; X=O
**3 (CysGlu):** R=H; X=S

**Figure S28 | Scheme for synthesis of dipeptides 1**, **2** and **3**. (i) 1-Ethyl-3-(3′-dimethylaminopropyl)carbodiimide (EDC), *N*-methylmorpholine, $CH_2Cl_2$, 0 °C to ambient temperature, 1 h ambient temperature. (ii) 50 equivalents of $CF_3CO_2H$ in $CH_2Cl_2$. PG = Side chain protecting group. PG = $^t$Bu for the synthesis of **1** and **2**. PG = trityl for synthesis of **3**.

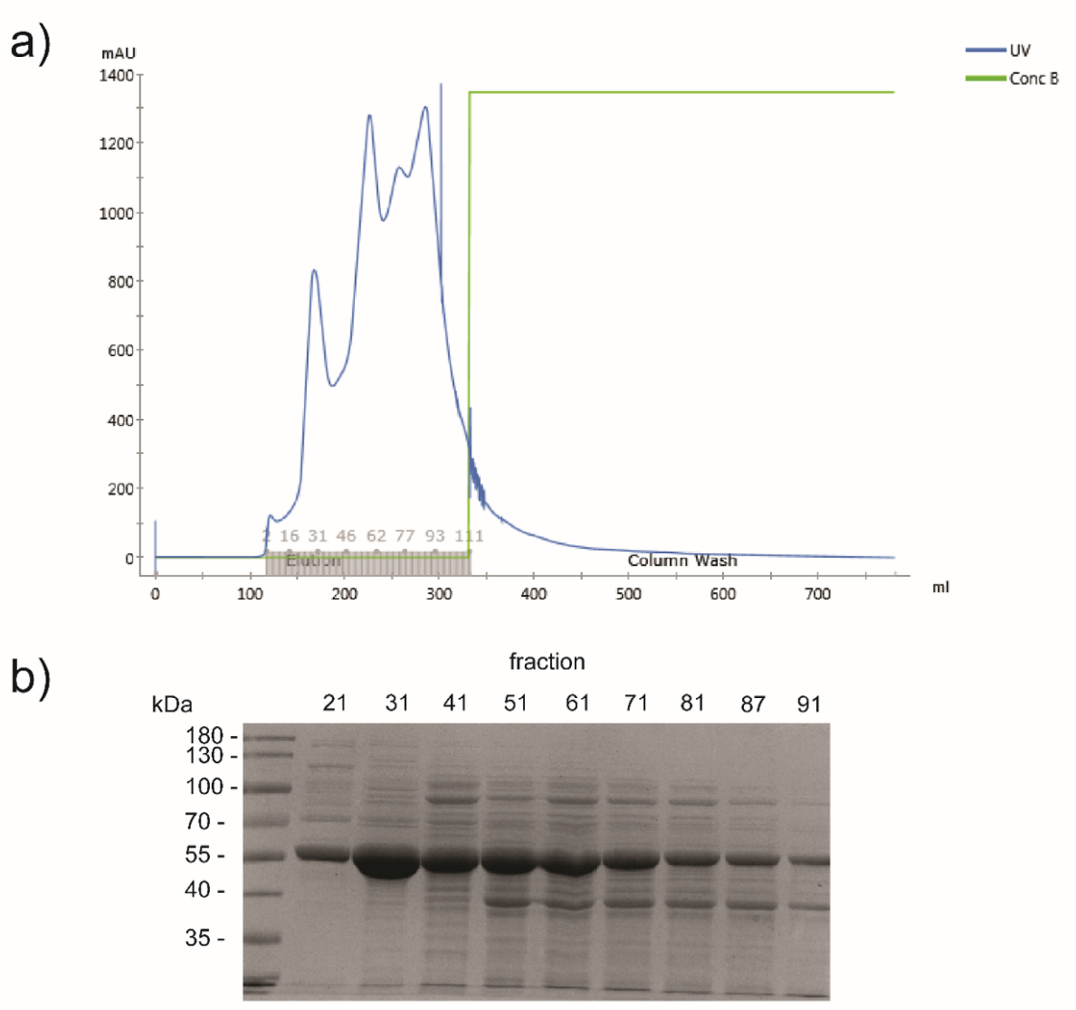


**Figure S29 | Size Exclusion Chromatography of ASNS B. a.** Elution profile showing UV trace. **b.** SDS-PAGE analysis of indicated fractions. Fractions 21-46 were pooled and concentrated for further studies. SPE-MS analysis suggests a mass of 63594.09 Da (calculated 63594.67 Da), corresponding to the mass of the protein with cleavage of the N-terminal methionine, including the C-terminal His-tag sequence (LEHHHHHH).

**a)**

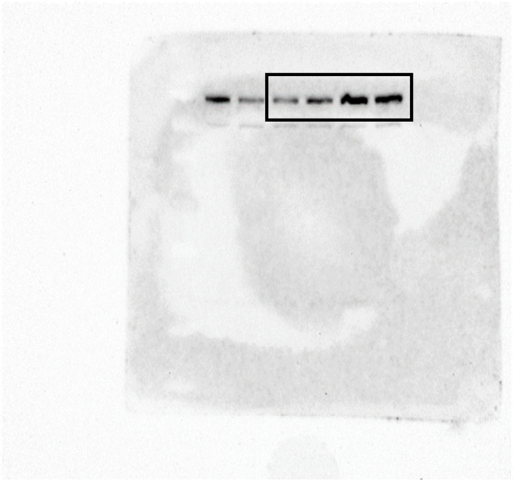

**b)**

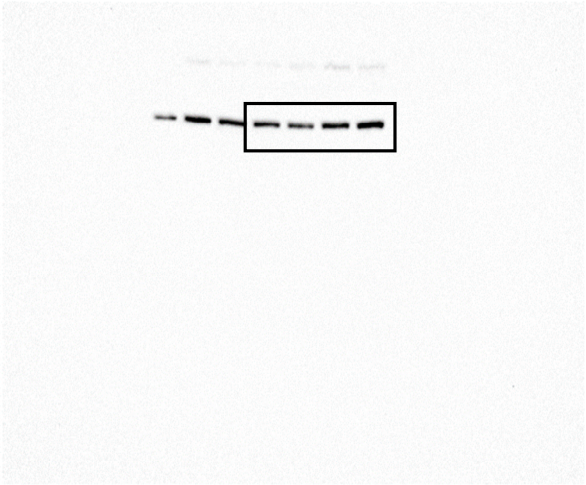

**c)**

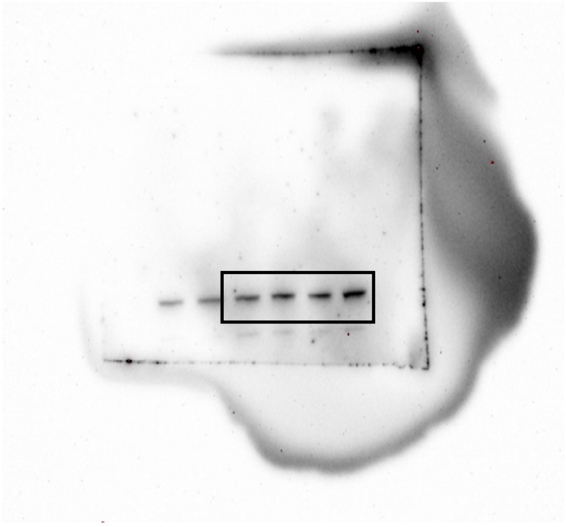

**d)**

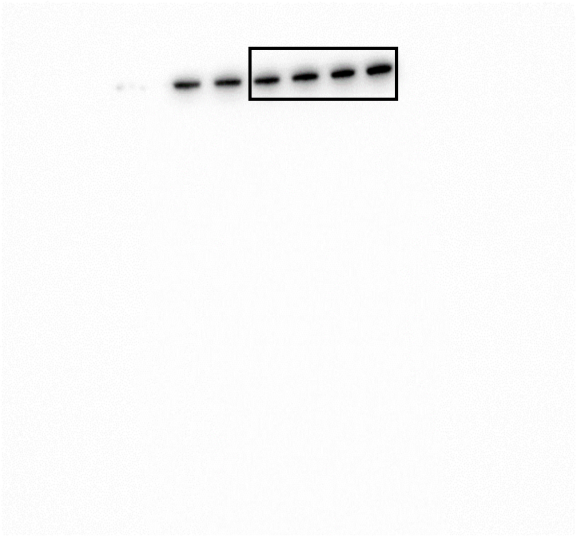

**e)**

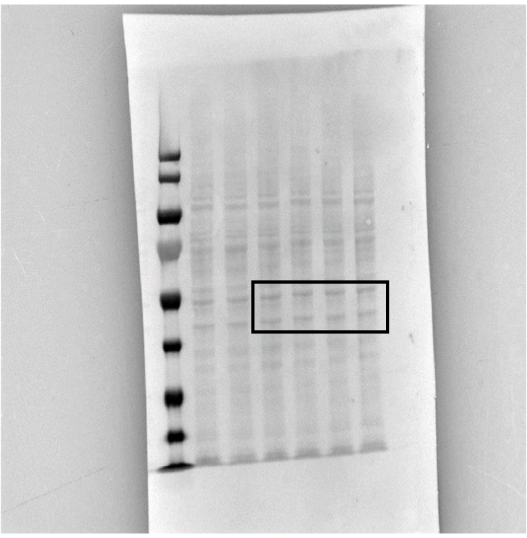

**f)**

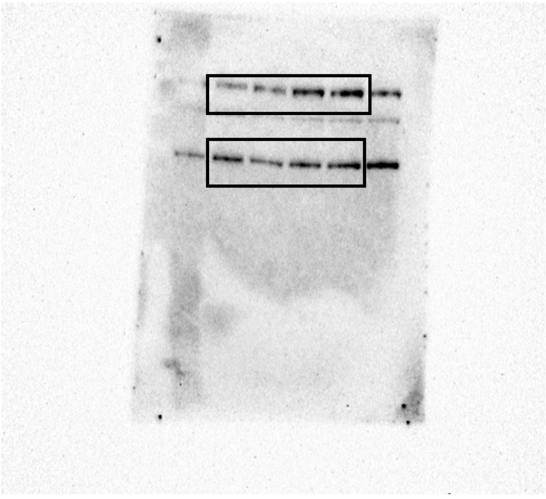

**g)**

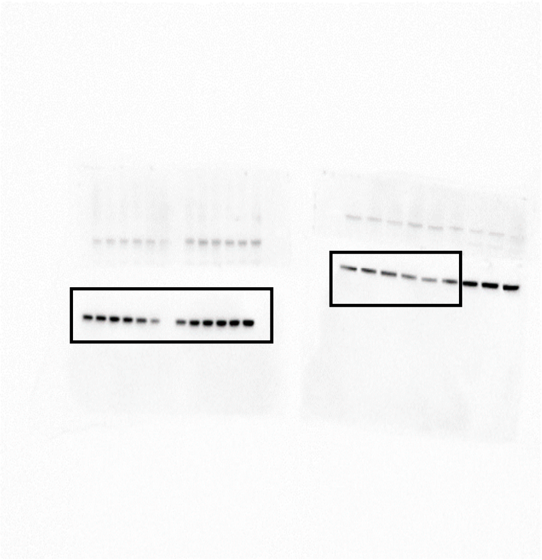

**h)**

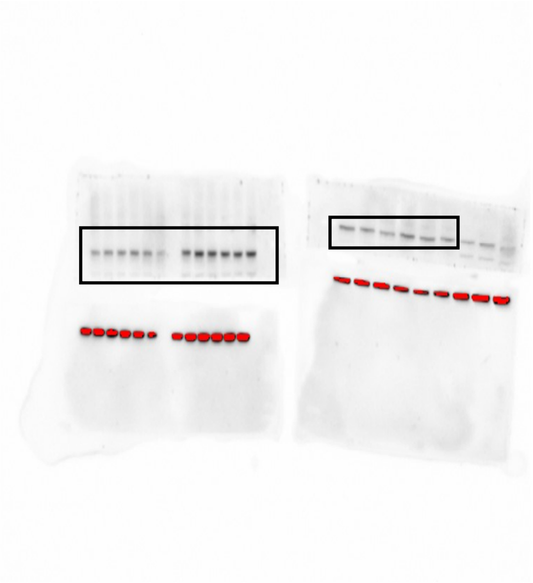

i)

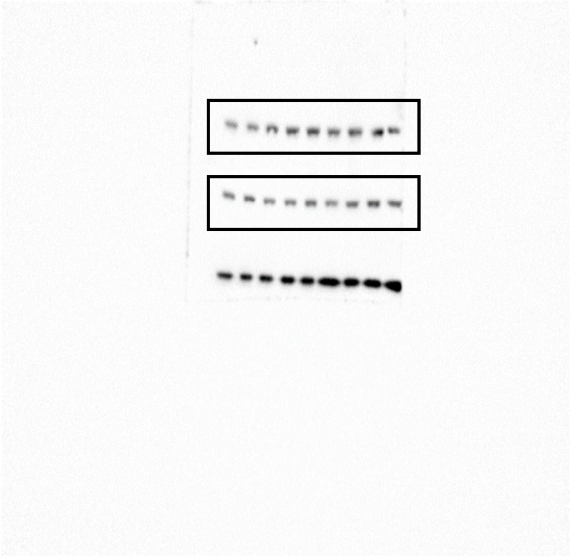

j)

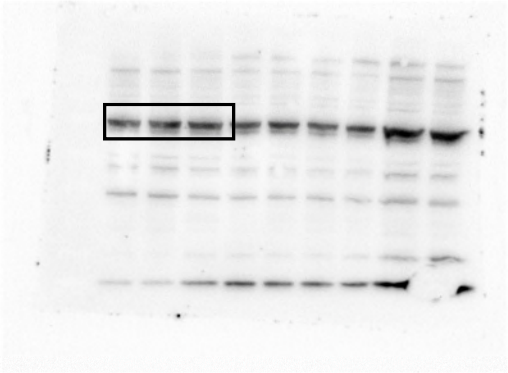

k)

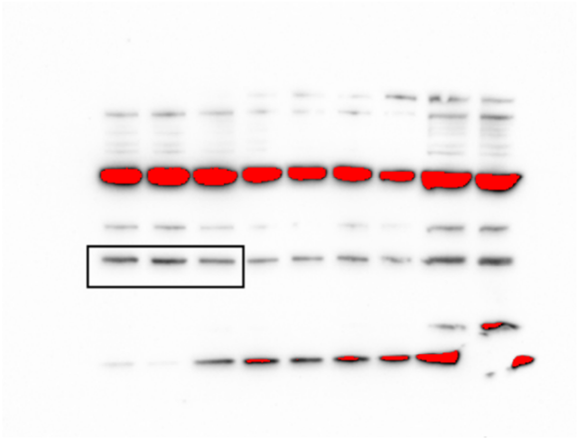

l)

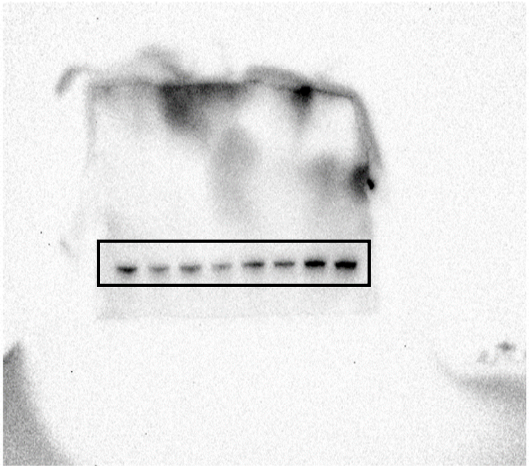

m)

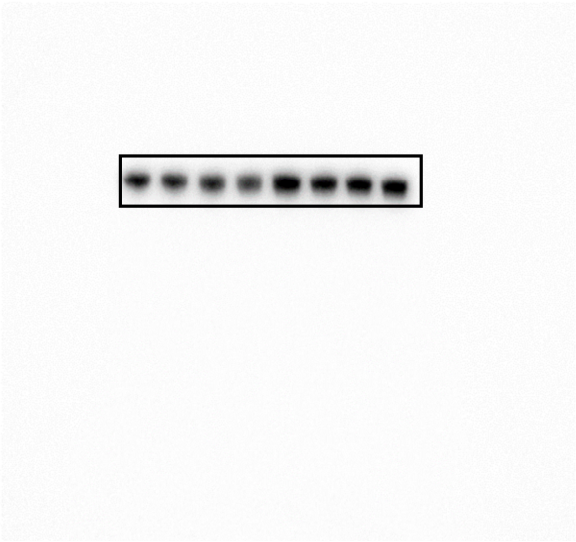

n)

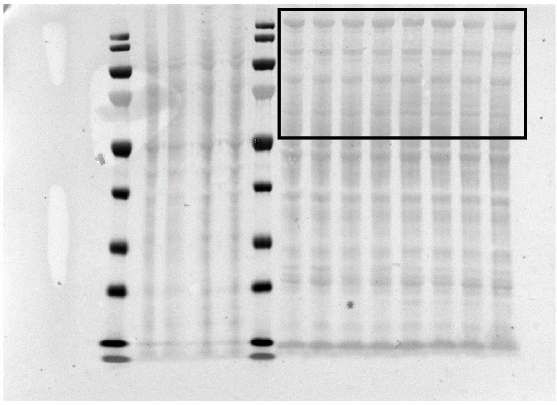

o)

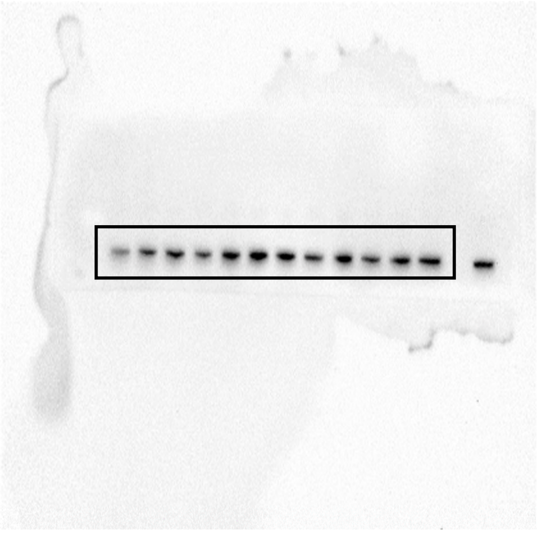

p)

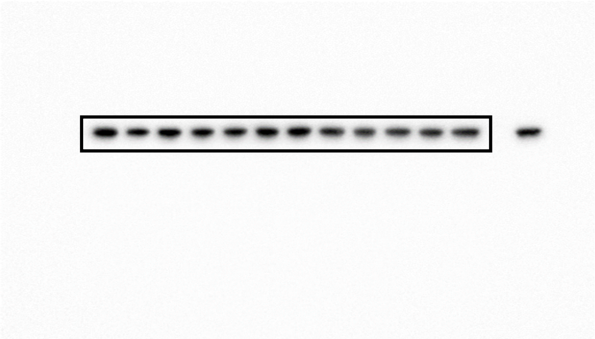

**q)**

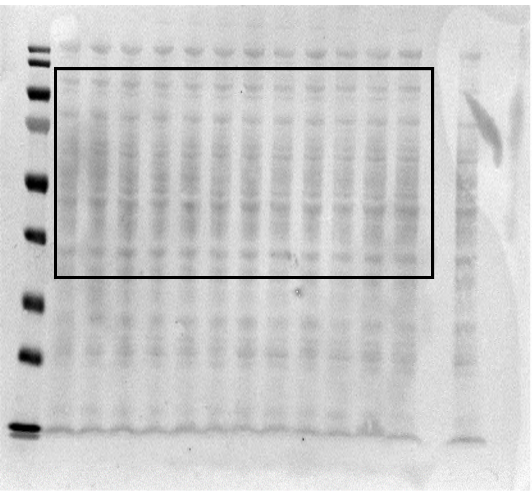

**r)**

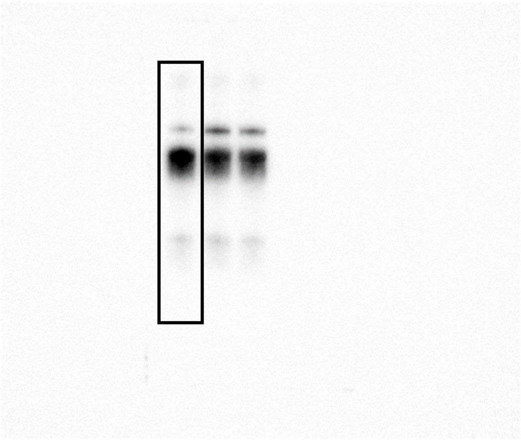

**s)**

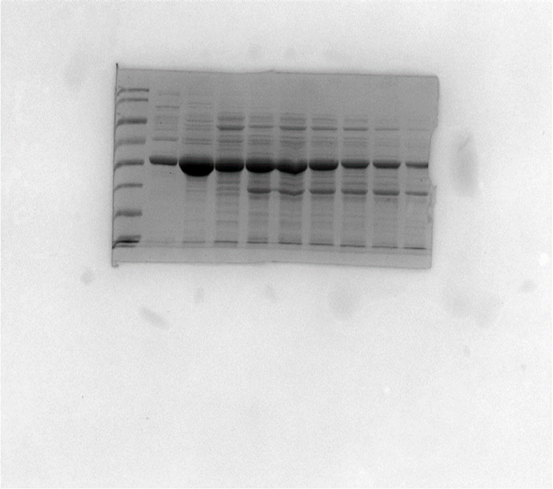

**Figure S30 | Raw images of western blots /electrophoresis (a – r).** Relevant parts of the images are highlighted by boxes. **a)** Figure 1d ASNS signals highlighted; **b)** Figure 1d GAPDH signals highlighted**;** **c)** Figure S8a ASNS signals highlighted; **d)** Figure S8a GAPDH signals highlighted; **e)** Figure S8a Ponceau stain signals highlighted**;** **f)** Figure S8b ASNS signals highlighted (top); GAPDH signals highlighted (bottom), **g)** Figure S9a GAPDH signals highlighted; **h)** Figure S9a ASNS signals highlighted; **i)** Figure S9b ASNS signals highlighted (top); GAPDH signals highlighted (bottom); **j)** Figure S9c ASNS signals highlighted; **k)** Figure S9c GAPDH signals highlighted; l**)** Figure S11b ASNS signals highlighted, **m)** Figure S11b GAPDH signals highlighted; **n)** Figure S11b Ponceau stain signals highlighted**,** **o)** Figure S11c ASNS signals highlighted; **p)** Figure S11c GAPDH signals highlighted, **q)** Figure S11c Ponceau stain signals highlighted**;** **r)** Figure S24a ASNS immunoprecipitation; **s)** Figure S29 - ASNS B SDS PAGE image.

**Table S1 | Buffers used for ASNS B purification.**

| Buffer | Components | Final Concentration |
|---|---|---|
| Anion Exchange A, pH 6.5 | BisTris | 50 mM |
| | NaCl | 0 mM |
| Anion Exchange B, pH 6.5 | BisTris | 50 mM |
| | NaCl | 1 M |
| Size Exclusion Buffer, pH 7.5 | BisTris | 50 mM |
| | NaCl | 150 mM |

**Table S2 | ASNS B NMR assay conditions.**

| Component | Stock | Volume [µl] | Final Conc. |
|---|---|---|---|
| ASNS B | 15.7 µM (1 mg/mL) | 10 | 314 nM |
| Carbonyl compound | 1 mM (example for 100-fold excess) | 15.7 | 31.4 µM |
| ATP | 50 mM | 10 | 1 mM |
| Gln (optional) | 50 mM | 10 | 1 mM |
| Asp | 50 mM | 10 | 1 mM |
| $MgCl_2$ | 100 mM | 50 | 10 mM |
| $NH_4Cl$ (optional) | 50 mM | 50 | 5 mM |
| 50 mM potassium phosphate, pD 8 | | Add to 500 µL | |

## References


1. T. R. Malla, A. Tumber, T. John, L. Brewitz, C. Strain-Damerell, C. D. Owen, P. Lukacik, H. T. H. Chan, P. Maheswaran, E. Salah, F. Duarte, H. Yang, Z. Rao, M. A. Walsh and C. J. Schofield, *Chemical Communications*, 2021, **57**, 1430-1433.
2. S. K. Bharti and R. Roy, *TrAC Trends in Analytical Chemistry*, 2012, **35**, 5-26.
3. V. Poláčková, P. Čmelová, R. Górová and R. Šebesta, *Monatshefte für Chemie - Chemical Monthly*, 2018, **149**, 729-736.
4. T. M. Larsen, S. K. Boehlein, S. M. Schuster, N. G. Richards, J. B. Thoden, H. M. Holden and I. Rayment, *Biochemistry*, 1999, **38**, 16146-16157.
5. N. Rabbani and P. J. Thornalley, *Nat. Protoc.*, 2014, **9**, 1969-1979.
